# Supplementary material for: Metal-Free Phosphination and Continued Functionalization of Pyridine: A Theoretical Study
Source: Molecules. 2022 Sep 3;27(17):5694. doi: 10.3390/molecules27175694 (PMC9457550; doi:10.3390/molecules27175694)
Supplement: Supplementary file 1 [file molecules-27-05694-s001.zip › molecules-1885281-supplementary.pdf]

Supporting Information

Metal-free Phosphination and Continued Functionalization of Pyridine: A Theoretical Study

Pan Du[1], Yuhao Yin[2], Dai Shi[2], Kexin Mao[2], Qianyuan Yu[2], Jiyang Zhao*[,2]

[1]School of Life Science and Chemistry, Jiangsu Second Normal University, Nanjing 210013, China

[2]School of Environmental Science, Nanjing Xiaozhuang University, Nanjing 211171, China

jyzhao1981@163.com

Table of Contents

Figure S1. Optimized intermediates and transition states of pyridine activation and nucleophilic addition………………………..…………………..…....………..…….s4

Figure S2. Optimized intermediates and transition states of dihydropyridine rearomatization to afford the final product (path A)………………………………...s5

Figure S3. Optimized intermediates and transition states of dihydropyridine rearomatization to afford the final product (path B)…………………...……..…….s6

Figure S4. Optimized intermediates and transition states of pyridine phosphination to afford C2-phosphonates……………………………………………..……...…s7

Figure S5. Optimized intermediates and transition states of phosphination of pyridine with $P(O\textit{i}Pr)_3$ to afford C4-phosphonates...……..……..……..……………..……….s8

Figure S6. Optimized intermediates and transition states of phosphination of pyridine with $P(O\textit{i}Pr)_3$ to afford C2-phosphonates……..……..……………..……....……….s9

Figure S7. Free energy profile of phosphination of pyridine with $P(O\textit{i}Pr)_3$ to afford C4-phosphonates……...………………………………………………………………...s10

Figure S8. Free energy profile of phosphination of pyridine with $P(O\textit{i}Pr)_3$ to afford C2-phosphonates...……………………………………………………………...…s11

Table S1. Interaction and strain energies of the **ipr-p-TS$_{7/8}$** and **ipr-o-TS$_{7/8}$** transition states (unit kcal/mol). ..……. …..……..……..…..………..…...…....…..…..………s12

Figure S9. Optimized intermediates and transition states of pyridine phosphination with PPh_3...……..…..……….……...…..…………………………......…..………s13

Figure S10. Optimized intermediates and transition states of 2-Ph-pyridine phosphination with PPh_3 to give the ortho-substituted product..…...…..…..………..s14

Figure S11. Optimized intermediates and transition states of Phosphination of 2-Ph-pyridine with diarylfluoroalkylphosphines P1–P5…..……..…………..…..………..s15

Figure S12. Optimized intermediates and transition states of The fluoroalkylation of pyridine in acidic solvent starting from a phosphonium salt.……..…........……….s16

Table S2. Corrected free energies of all species…………………….....……………..s17

Table S3. Imaginary frequencies of all transition states. ……………...……....….s20

Table S4. Cartesian coordinates of all species……………………………..…..….s21

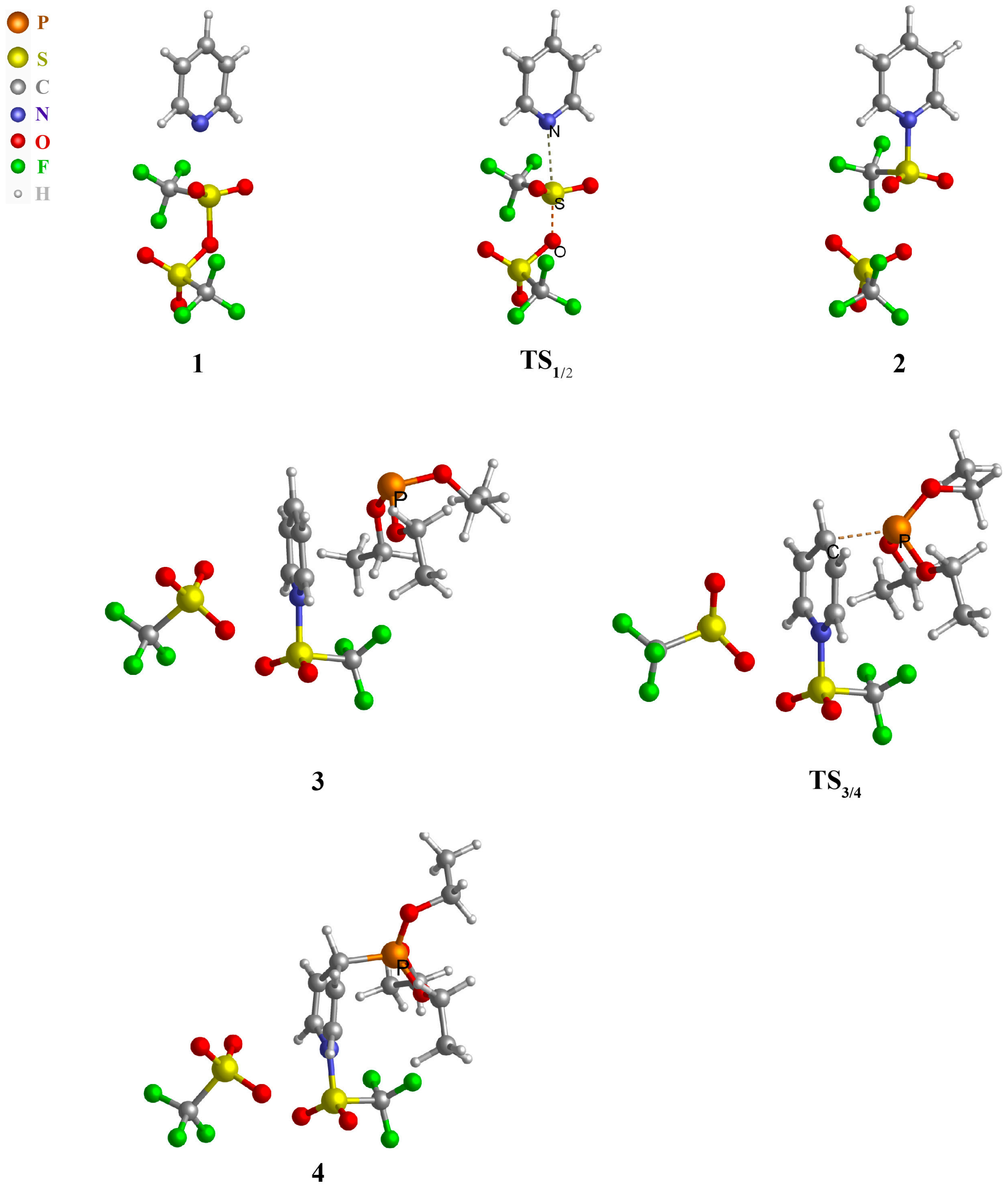

Figure S1. Optimized intermediates and transition states of pyridine activation and nucleophilic addition.

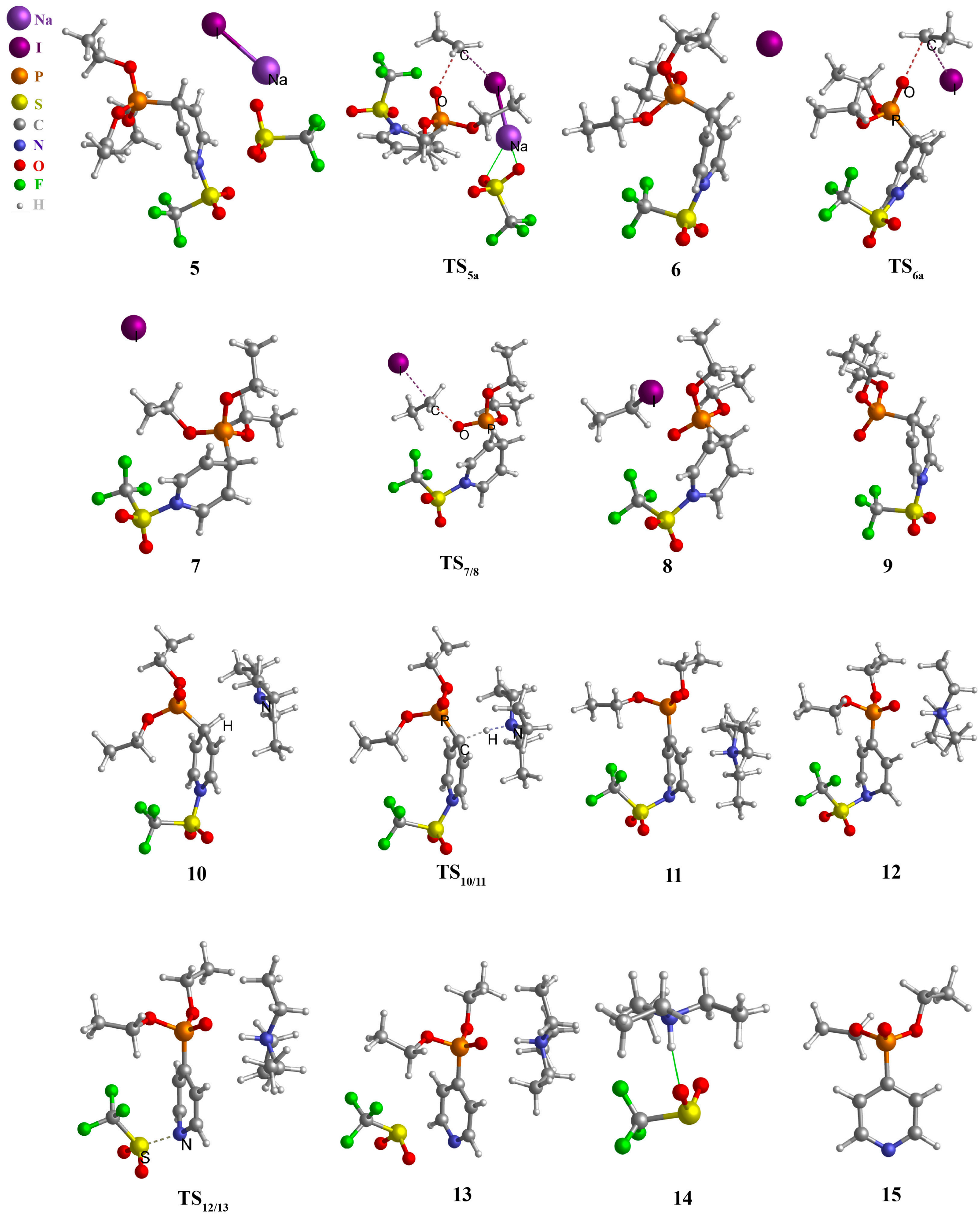

Figure S2. Optimized intermediates and transition states of dihydropyridine rearomatization to afford the final product (path A).

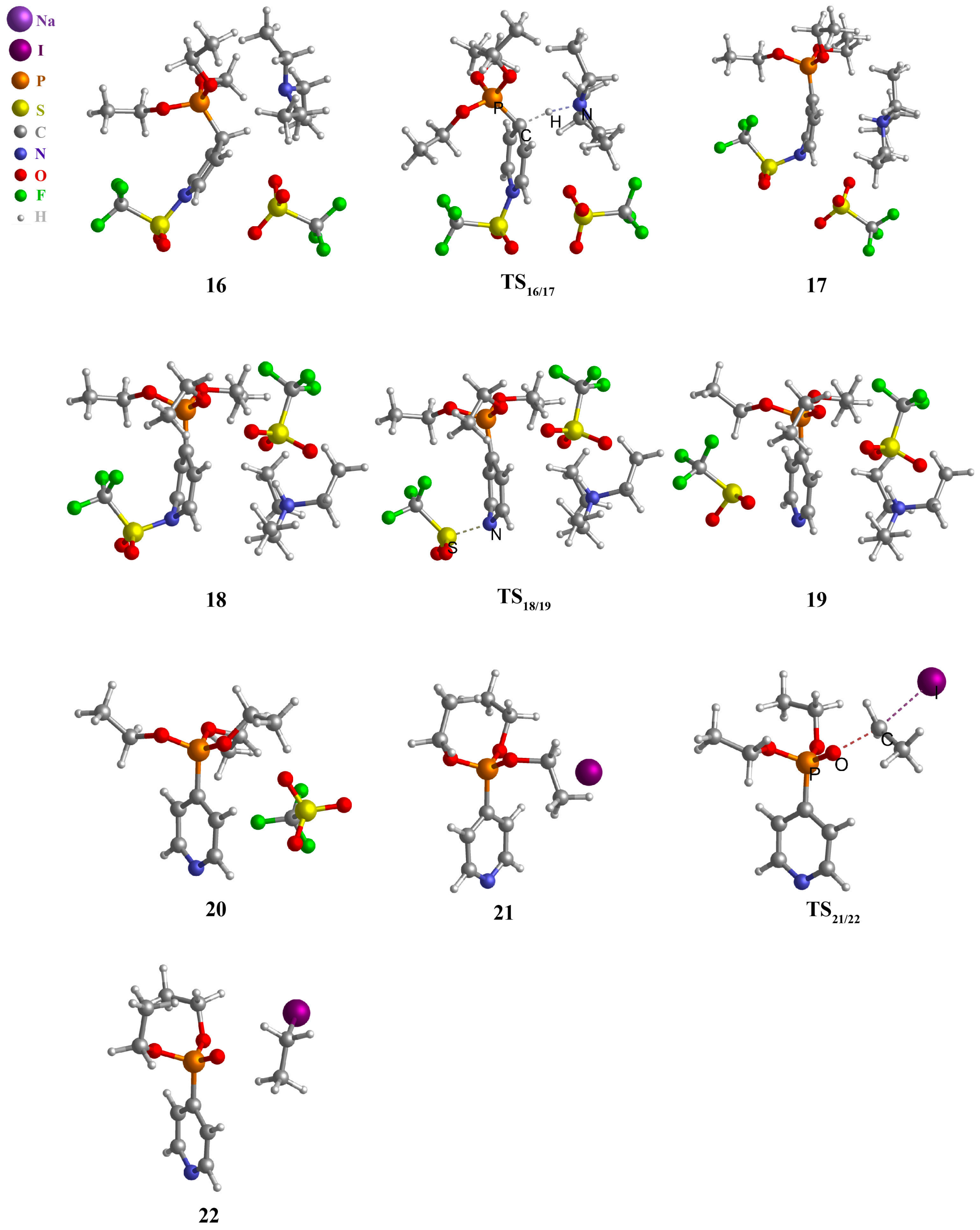

Figure S3. Optimized intermediates and transition states of dihydropyridine rearomatization to afford the final product (path B).

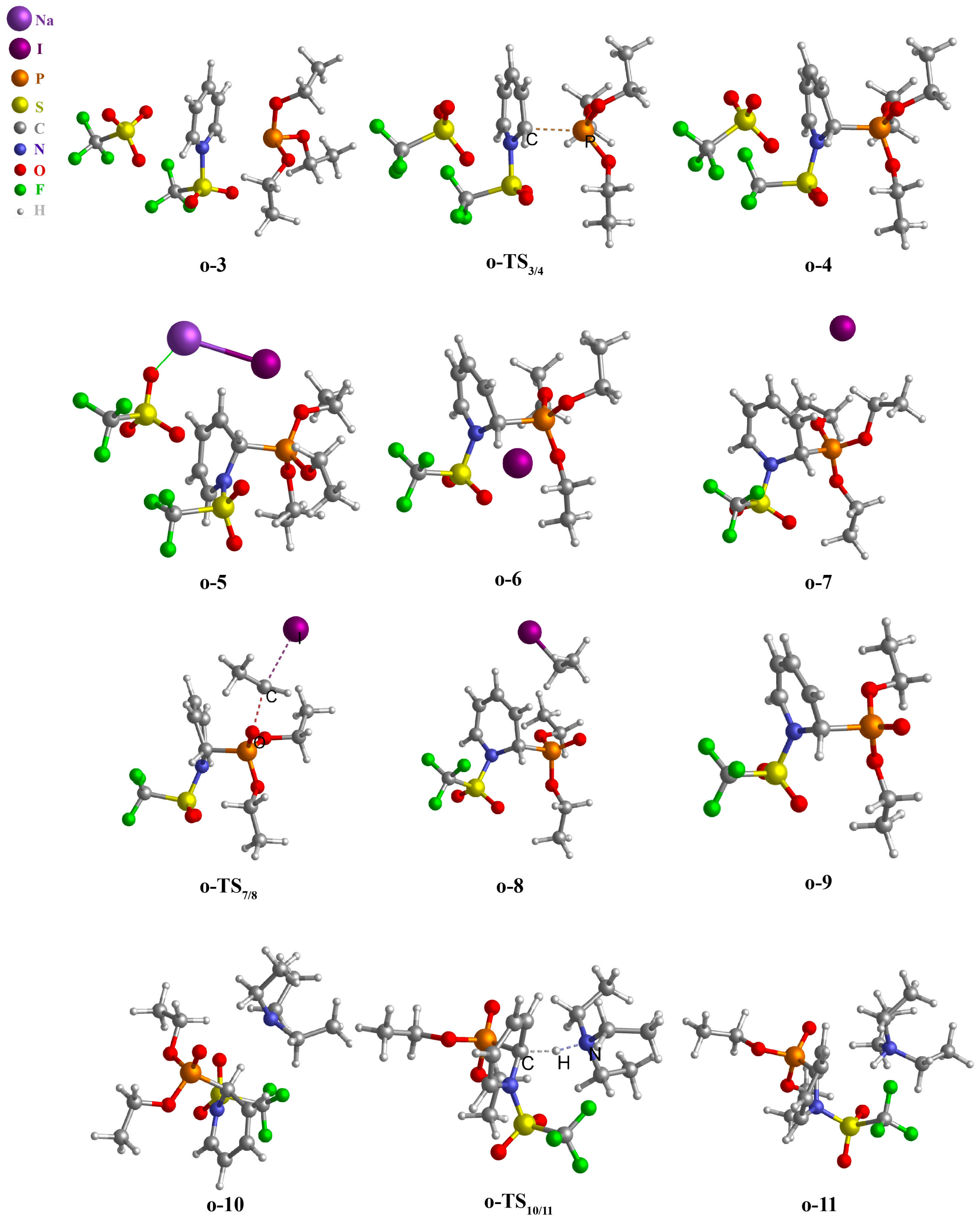

Figure S4. Optimized intermediates and transition states of pyridine phosphination to afford C2-phosphonates.

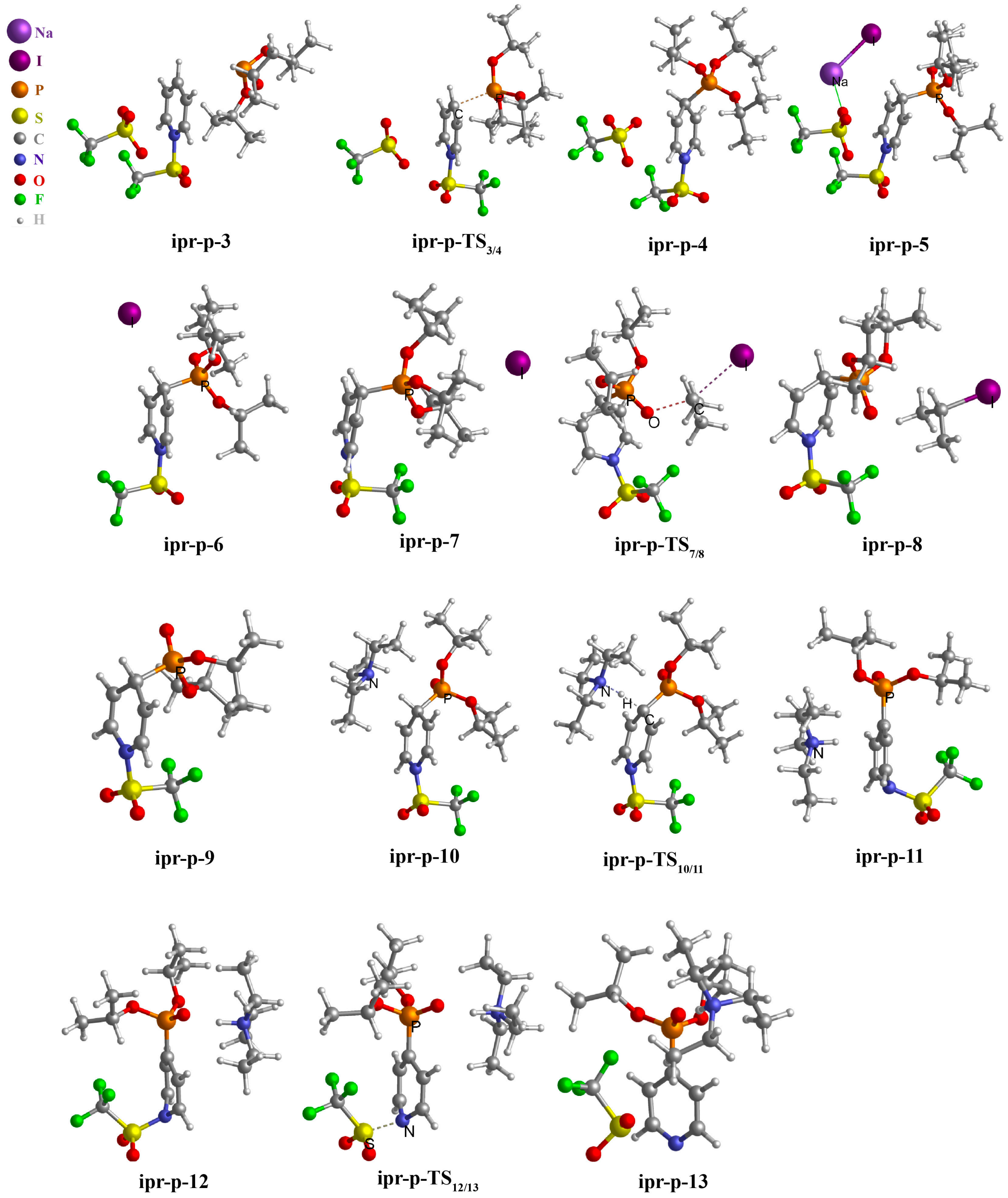

Figure S5. Optimized intermediates and transition states of phosphination of pyridine with P(O*i*Pr)$_3$ to afford C4-phosphonates.

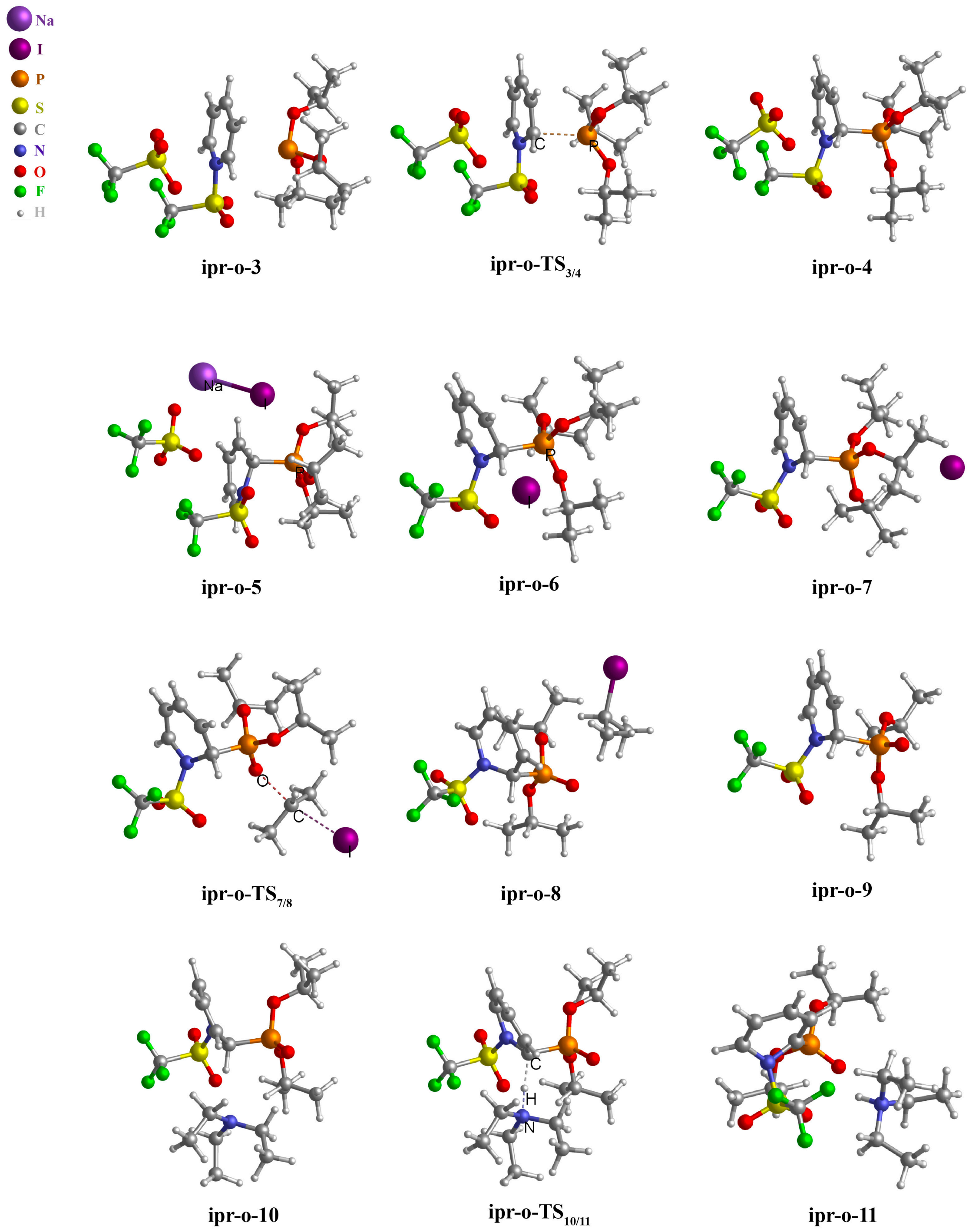

Figure S6. Optimized intermediates and transition states of phosphination of pyridine with P(O*i*Pr)3 to afford C2-phosphonates.

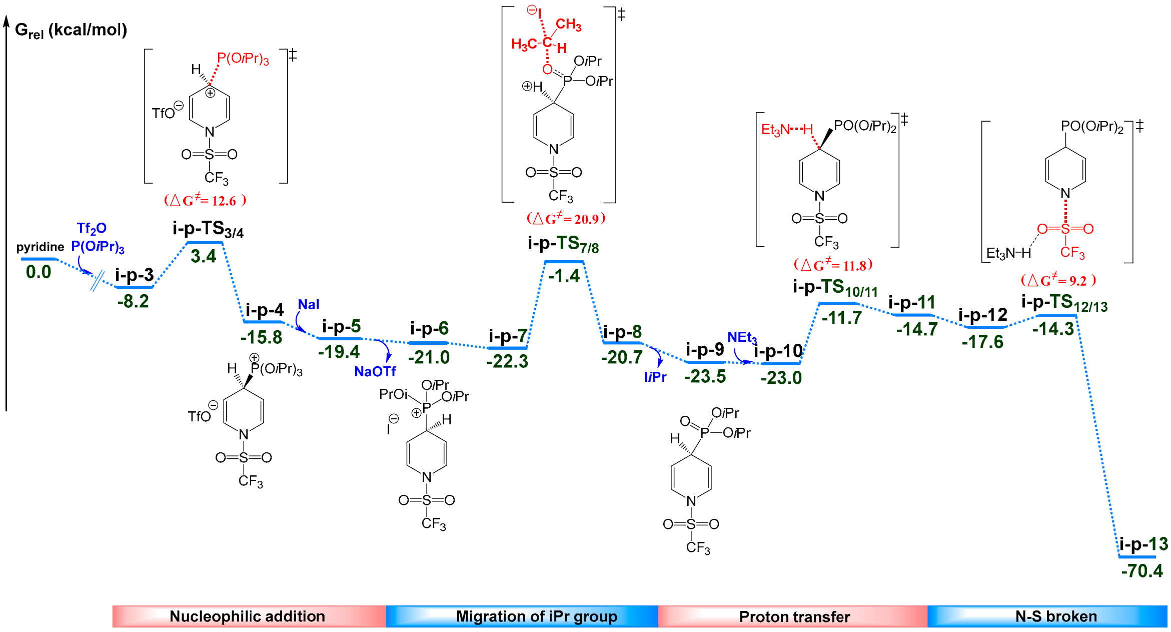

Figure S7. Free energy profile of phosphination of pyridine with P(O*i*Pr)3 to afford C4-phosphonates.

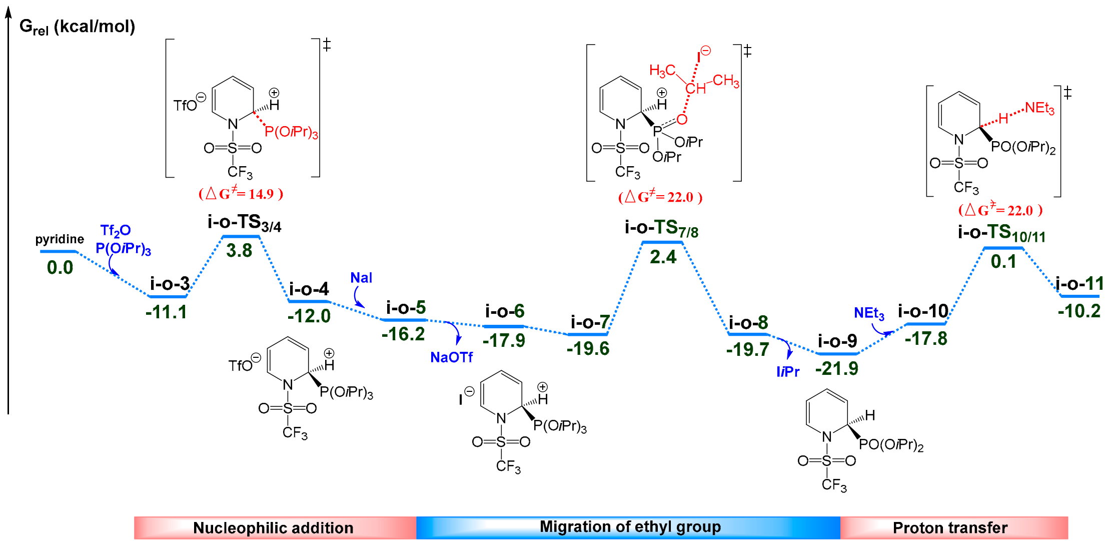

Figure S8. Free energy profile of phosphination of pyridine with P(O*i*Pr)3 to afford C2-phosphonates.

Table S1. Interaction and strain energies of the **ipr-p-TS$_{7/8}$** and **ipr-o-TS$_{7/8}$** transition states (unit kcal/mol).

	E_{inter}	E_{strain}		
		phosphorus	pyridine	sum
ipr-p-TS$_{7/8}$	-111.2	66.7	45.6	112.3
ipr-o-TS$_{7/8}$	-119.3	73.7	48.1	121.8
differ	-8.1	7.0	2.5	9.5

The phosphination of pyridine with P(O*i*Pr)$_3$ to afford C2- and C4-phosphonates were explored. Based on the experiments conducted in a previous study, the ratio of C4- and C2-phosphonates is 60:40 when the phosphate is P(O*i*Pr)$_3$. As shown in Figures S5-S8, the calculated results demonstrated that the free energy barrier of the rate-limiting step in the formation of C4-phosphonate is a little lower than that of C2-phosphonate (**ipr-p-TS$_{7/8}$: ipr-o-TS$_{7/8}$** = 20.9:22.0 kcal/mol). The energy decomposition analysis in the two transion states (**ipr-p-TS$_{7/8}$** and **ipr-o-TS$_{7/8}$**) indicates that the difference of the interaction energies in the two transion states is close to the strain energies of the phosphorus and pyridine moieties (8.1:9.5, as shown in Table S1). Thus, the yields of C2- and C4-phosphonates are close.

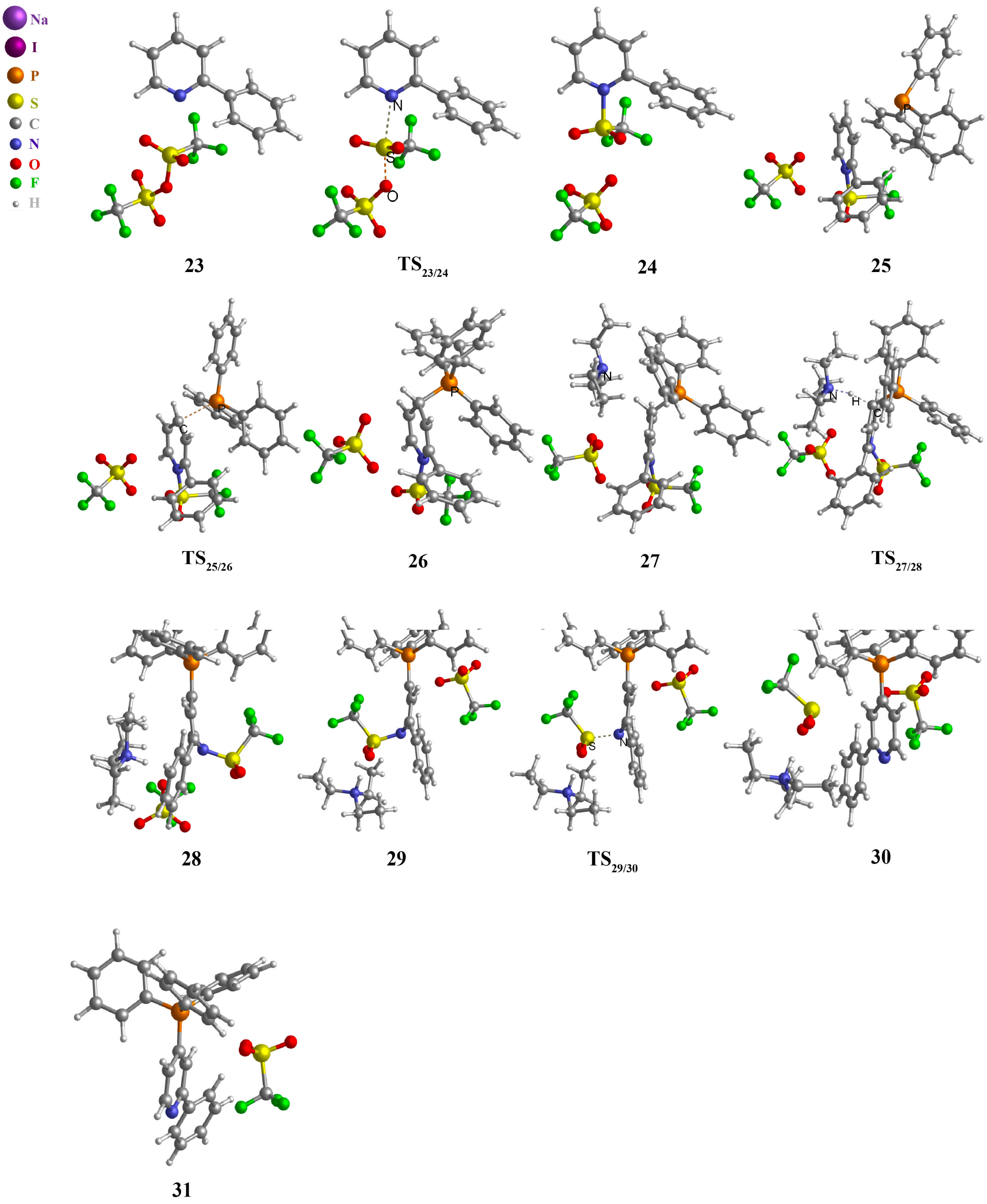

Figure S9. Optimized intermediates and transition states of pyridine phosphination with PPh_3.

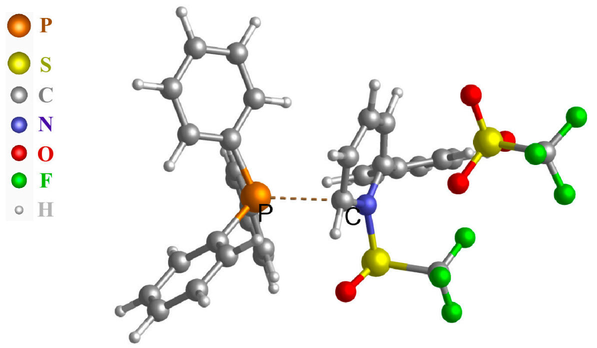

$TS_{24/32}$

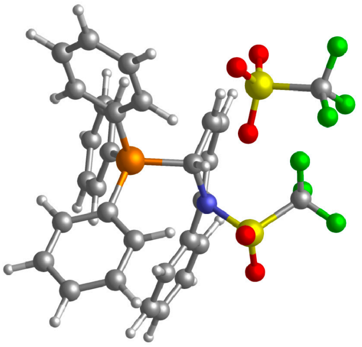

32

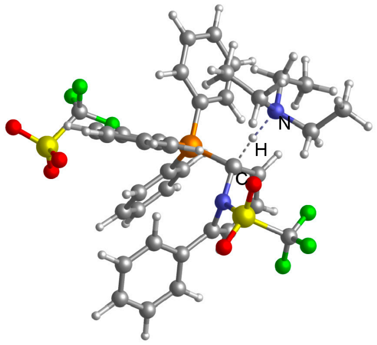

$TS_{32/33}$

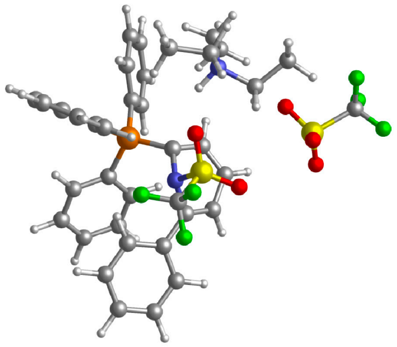

33

Figure S10. Optimized intermediates and transition states of 2-Ph-pyridine phosphination with PPh_3 to give the ortho-substituted product.

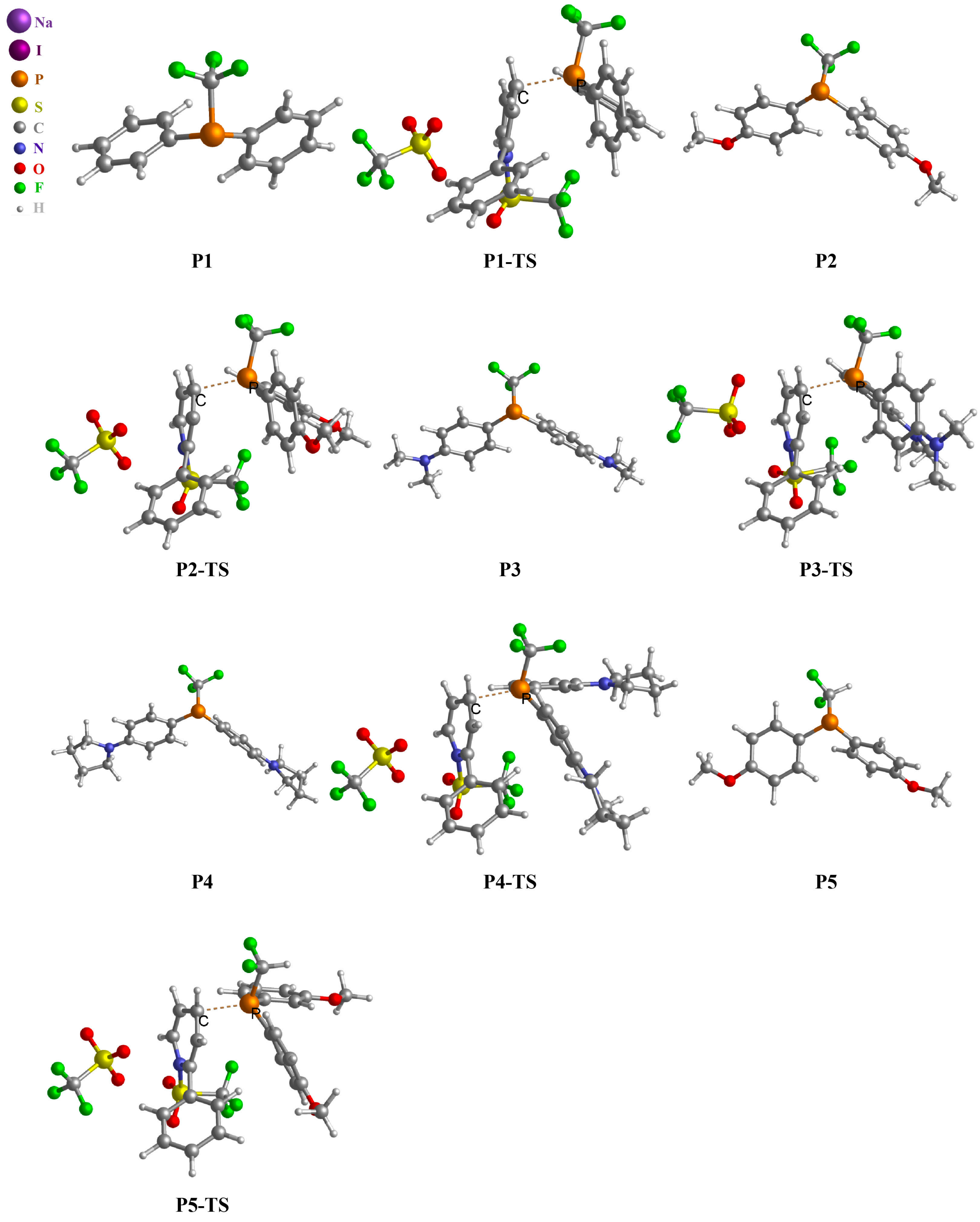

Figure S11. Optimized intermediates and transition states of Phosphination of 2-Ph-pyridine with diarylfluoroalkylphosphines P1–P5.

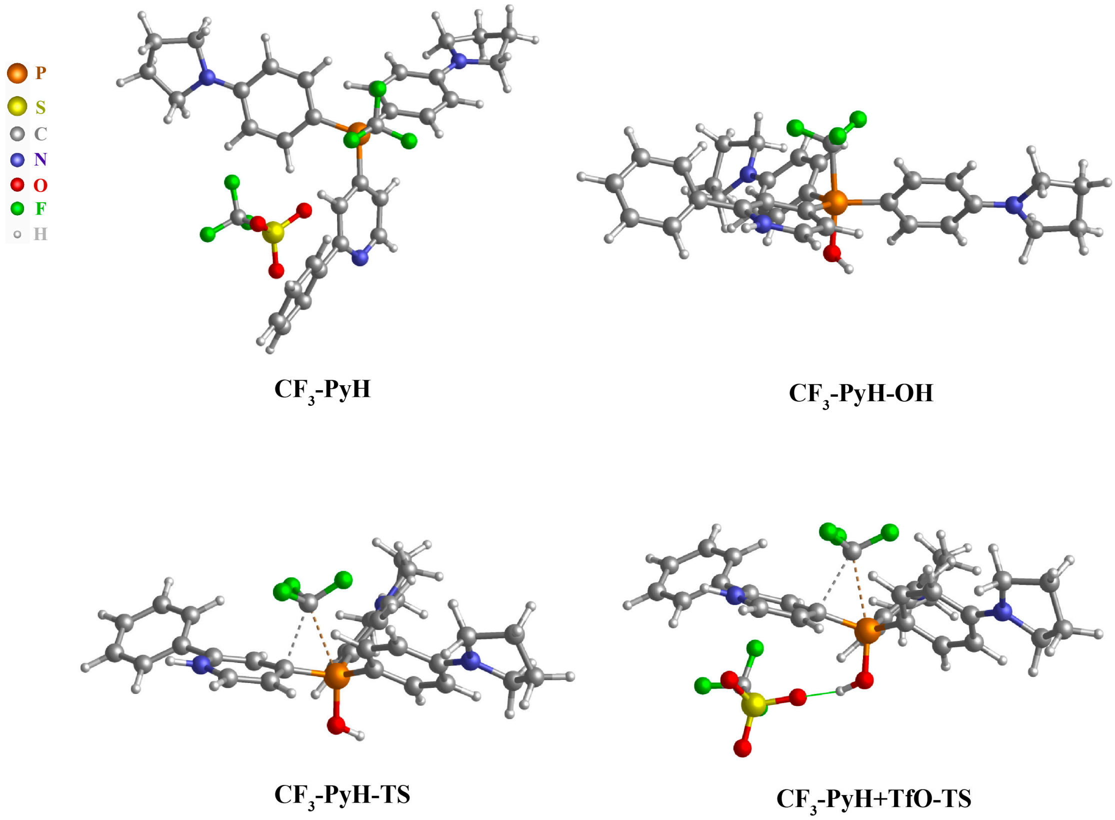

Figure S12. Optimized intermediates and transition states of The fluoroalkylation of pyridine in acidic solvent starting from a phosphonium salt.

Table S2. Free energies of all species (a.u.).

Species	Free energies	Species	Free energies
**Pyridine**	-248.1816218	**ipr-p-13**	-2230.4958989
**Tf_2O**	-1847.5470134	**ipr-o-3**	-3018.0701379
**PPh_3**	-1035.9425268	**ipr-o-$TS_{3/4}$**	-3018.0463581
**NEt_3**	-292.1696208	**ipr-o-4**	-3018.0715589
**$POEt_3$**	-804.4836550	**ipr-o-5**	-3476.1239601
**1**	-2095.7214066	**ipr-o-6**	-2352.3132415
**$TS_{1/2}$**	-2095.7197605	**ipr-o-7**	-2352.3159838
**2**	-2095.7355690	**ipr-o-$TS_{7/8}$**	-2352.2808545
**3**	-2900.2159230	**ipr-o-8**	-2352.3161462
**$TS_{3/4}$**	-2900.1983318	**ipr-o-9**	-1938.2560634
**4**	-2900.2231594	**ipr-o-10**	-2230.4120052
**5**	-3358.2763830	**ipr-o-$TS_{10/11}$**	-2230.3835649
**6**	-2234.4650645	**ipr-o-11**	-2230.3999447
**7**	-2234.4601342	**2-Ph-pyridine**	-479.1283499
**$TS_{7/8}$**	-2234.4332245	**23**	-2326.6659824
**8**	-2234.4731069	**$TS_{23/24}$**	-2326.6561552
**9**	-1859.6931745	**24**	-2326.6757330
**10**	-2151.8539668	**25**	-3362.6126939
**$TS_{10/11}$**	-2151.8349567	**$TS_{25/26}$**	-3362.6001526
**11**	-2151.8403013	**26**	-3362.6223119
**12**	-2151.8450201	**27**	-3654.7838898
**$TS_{12/13}$**	-2151.8460920	**$TS_{27/28}$**	-3654.7586402
**13**	-2151.9282372	**28**	-3654.7907389
**14**	-1178.9926980	**29**	-3654.7888391
**15**	-972.9561688	**$TS_{29/30}$**	-3654.7912684
**Iet**	-374.7905277	**30**	-3654.8788197

**NaI**	-458.0527020	**31**	-2475.8964554
**NaOTf**	-1123.8204618	**$TS_{24/32}$**	-3362.5998418
**TS_{5a}**	-3358.2121411	**32**	-3362.6269359
**TS_{6a}**	-2234.4079128	**$TS_{32/33}$**	-3654.7534324
**16**	-3192.3858293	**33**	-3654.7842838
**$TS_{16/17}$**	-3192.3716340	**DBU**	-461.7933423
**17**	-3192.3856776	**P1**	-1142.030133
**18**	-3192.3959128	**P1-TS-R**	-3468.665439
**$TS_{18/19}$**	-3192.3893474	**P1-TS**	-3468.633347
**19**	-3192.4668674	**P1-TS-P**	-3468.643278
**20**	-2013.4887665	**P2**	-1370.997395
**21**	-1347.7239461	**P2-TS-R**	-3697.6315980
**$TS_{21/22}$**	-1347.6954983	**P2-TS**	-3697.6025670
**22**	-1347.7351413	**P2-TS-P**	-3697.621953
**o-3**	-2900.2199209	**P3**	-1409.784631
**o-$TS_{3/4}$**	-2900.1980379	**P3-TS-R**	-3736.4122370
**o-4**	-2900.2214071	**P3-TS**	-3736.3920470
**o-5**	-3358.2692277	**P3-TS-P**	-3736.419580
**o-6**	-2234.4616355	**P4**	-1564.543537
**o-7**	-2234.4589242	**P4-TS-R**	-3891.1722460
**o-$TS_{7/8}$**	-2234.4267360	**P4-TS**	-3891.1545950
**o-8**	-2234.4709367	**P4-TS-P**	-3891.176461
**o-9**	-1859.6911452	**P5**	-1271.733425
**o-10**	-2151.8447953	**P5-TS-R**	-3598.3627890
**o-$TS_{10/11}$**	-2151.8186801	**P5-TS**	-3598.3447310
**o-11**	-2151.8270218	**P5-TS-P**	-3598.366964
**ipr-p-3**	-3018.0654800	**H_2O**	-76.415661
**ipr-p-$TS_{3/4}$**	-3018.0470978	**CF_3-PyH**	-3004.467773
**ipr-p-4**	-3018.0775944	**CF_3-PyH-OH**	-2119.280671
**ipr-p-5**	-3476.1290744	**CF_3-PyH-TS**	-2119.261294

**ipr-p-6**	-2352.3181470	**CF_3-PyH+TfO-TS**	-3080.848952
**ipr-p-7**	-2352.3201827		
**ipr-p-$TS_{7/8}$**	-2352.2869472		
**ipr-p-8**	-2352.3177688		
**ipr-p-9**	-1938.2587729		
**ipr-p-10**	-2230.4203756		
**ipr-p-$TS_{10/11}$**	-2230.4023220		
**ipr-p-11**	-2230.4071968		
**ipr-p-12**	-2230.4116901		
**ipr-p-$TS_{12/13}$**	-2230.4065156		

Table S3. Imaginary frequencies of all transition states.

Species	Imaginary frequency	Species	Imaginary frequency
**$TS_{1/2}$**	23.00 cm^{-1}	**ipr-o-$TS_{10/11}$**	1163.75 cm^{-1}
**$TS_{3/4}$**	232.28 cm^{-1}	**$TS_{23/24}$**	114.88 cm^{-1}
**$TS_{7/8}$**	555.18 cm^{-1}	**$TS_{25/26}$**	150.81 cm^{-1}
**$TS_{10/11}$**	1077.94 cm^{-1}	**$TS_{27/28}$**	1287.74 cm^{-1}
**$TS_{12/13}$**	391.94 cm^{-1}	**$TS_{29/30}$**	376.62 cm^{-1}
**TS_{5a}**	317.94 cm^{-1}	**$TS_{24/32}$**	176.25 cm^{-1}
**TS_{6a}**	335.58 cm^{-1}	**$TS_{32/33}$**	1419.73 cm^{-1}
**$TS_{16/17}$**	1275.11 cm^{-1}	**P1-TS**	203.32 cm^{-1}
**$TS_{18/19}$**	392.71 cm^{-1}	**P2-TS**	204.08 cm^{-1}
**$TS_{21/22}$**	563.16 cm^{-1}	**P3-TS**	154.03 cm^{-1}
**o-$TS_{3/4}$**	251.26 cm^{-1}	**P4-TS**	200.71 cm^{-1}
**o-$TS_{7/8}$**	560.58 cm^{-1}	**P5-TS**	203.59 cm^{-1}
**o-$TS_{10/11}$**	1117.43 cm^{-1}	**CF_3-PyH-TS**	259.02 cm^{-1}
**ipr-p-$TS_{3/4}$**	213.98 cm^{-1}	**CF_3-PyH+TfO-TS**	222.14 cm^{-1}
**ipr-p-$TS_{7/8}$**	381.77 cm^{-1}		
**ipr-p-$TS_{10/11}$**	1080.00 cm^{-1}		
**ipr-p-$TS_{12/13}$**	392.93 cm^{-1}		
**ipr-o-$TS_{3/4}$**	245.89 cm^{-1}		
**ipr-o-$TS_{7/8}$**	368.06 cm^{-1}		

Table S4. Cartesian coordinates of all species (angstroms).

10

Symbol	X	Y	Z
C	-1.259383	-1.211020	1.068574
C	-0.031107	-0.737699	1.238469
C	0.765918	-0.088941	0.138214
C	0.049878	-0.164286	-1.182050
C	-1.183014	-0.637016	-1.316203
N	-1.907596	-1.139442	-0.197196
H	1.742805	-0.603224	0.055002
H	-1.825737	-1.708790	1.842285
H	0.431970	-0.851760	2.210891
H	0.571798	0.161496	-2.073337
S	-3.518713	-1.425087	-0.319522
O	-3.837289	-1.710052	-1.693718
O	-3.926148	-2.264554	0.775827
C	-4.247965	0.246975	0.023944
F	-5.562114	0.186942	-0.044979
F	-3.789200	1.105191	-0.874035
F	-3.879193	0.640548	1.232977
P	1.231018	1.614979	0.566698
O	2.427298	1.879959	-0.450108
O	0.106063	2.599378	-0.020936
C	3.029590	3.193840	-0.544945
H	2.327300	3.850846	-1.060803
H	3.200058	3.576400	0.464013
C	4.326405	3.048375	-1.304326
H	5.005353	2.380721	-0.771866
H	4.142166	2.641069	-2.299480
H	4.802541	4.024577	-1.408332
C	-1.188513	2.640361	0.617834
H	-1.058488	2.936018	1.661051
H	-1.621138	1.636004	0.591564
C	-2.049343	3.620402	-0.142087
H	-1.606423	4.616578	-0.112070
H	-2.150836	3.306408	-1.181846
H	-3.042814	3.664439	0.307202
H	-1.695520	-0.719563	-2.263757
O	1.521333	1.826130	2.005513
N	3.363615	-1.931685	0.031984
C	3.045168	-3.035565	-0.876961
H	3.042731	-2.637482	-1.892025
H	3.835034	-3.807542	-0.834777
C	4.614897	-1.272478	-0.348164
H	4.806234	-0.482610	0.379484
H	5.460132	-1.982033	-0.286376
C	3.391103	-2.386009	1.424589
H	2.423806	-2.844562	1.637588
H	4.156112	-3.171983	1.560360
C	3.613476	-1.265832	2.436799
H	3.380906	-1.632691	3.438304
H	4.644392	-0.910635	2.450526
H	2.960525	-0.412874	2.228850
C	1.683381	-3.675627	-0.620369
H	1.426004	-4.325820	-1.458429
H	1.666732	-4.284931	0.283829
H	0.908175	-2.908685	-0.535258
C	4.567073	-0.629118	-1.730459
H	5.441897	0.010446	-1.864179
H	4.570779	-1.360890	-2.538806
H	3.674637	-0.003511	-1.816690

11

Symbol	X	Y	Z
C	-0.995069	-1.572143	1.124391
C	-0.227762	-0.485275	1.367644
C	0.192918	0.375455	0.303560
C	0.002965	-0.118624	-1.028106
C	-0.755411	-1.214532	-1.264753
N	-1.593115	-1.706504	-0.189627
H	1.538867	-1.563337	-0.025650
H	-1.230966	-2.345922	1.839912
H	0.158396	-0.325281	2.370071
H	0.563745	0.318823	-1.848958
S	-3.204945	-1.317134	-0.293896
O	-3.634978	-1.519406	-1.661753
O	-3.895638	-1.938885	0.816522
C	-3.460003	0.513407	-0.007526
F	-4.753554	0.782181	-0.140758
F	-2.783247	1.229020	-0.893484
F	-3.078183	0.853153	1.214518
P	1.153739	1.782414	0.622075
O	2.436817	1.646712	-0.346672
O	0.468731	3.070507	-0.081411

C	3.332495	2.767440	-0.503658
H	2.792912	3.575389	-1.000952
H	3.645937	3.110093	0.485868
C	4.514918	2.306495	-1.323763
H	5.045600	1.498820	-0.815831
H	4.183706	1.953603	-2.301767
H	5.208650	3.135837	-1.471094
C	-0.770408	3.556574	0.464548
H	-0.546990	4.186898	1.328199
H	-1.373382	2.707142	0.799411
C	-1.490958	4.329123	-0.616929
H	-0.880755	5.165624	-0.961120
H	-1.706046	3.676867	-1.464397
H	-2.433266	4.721119	-0.230066
H	-0.825153	-1.738359	-2.206601
O	1.477156	2.013993	2.058135
N	2.355033	-2.190296	0.041114
C	2.047829	-3.420613	-0.772019
H	1.748772	-3.063258	-1.755468
H	2.990657	-3.963180	-0.866753
C	3.541387	-1.444615	-0.514119
H	3.572063	-0.492006	0.010643
H	4.421075	-2.034365	-0.248655
C	2.545819	-2.515845	1.499540
H	1.573329	-2.845208	1.860855
H	3.240455	-3.357591	1.536062
C	3.050848	-1.344481	2.325707
H	2.956434	-1.614598	3.377990
H	4.099982	-1.125287	2.130515
H	2.464214	-0.438370	2.158782
C	0.956305	-4.298598	-0.187868
H	0.745779	-5.085564	-0.912937
H	1.257069	-4.776044	0.743951
H	0.036896	-3.734118	-0.025910
C	3.466066	-1.194661	-2.008352
H	4.300332	-0.545811	-2.275755
H	3.553310	-2.108035	-2.595218
H	2.544525	-0.675544	-2.275181

12

Symbol	X	Y	Z
C	1.371991	-2.057790	-0.030302
C	0.415571	-1.176935	-0.393805
C	0.183790	0.015309	0.368579
C	0.746775	0.039334	1.689628
C	1.704557	-0.840236	2.044847
N	2.303743	-1.646011	1.000134
H	-2.742553	-0.776802	-0.697095
H	1.516417	-3.042283	-0.448672
H	-0.243376	-1.424470	-1.221884
H	0.313103	0.684645	2.447351
S	3.795761	-1.162735	0.472531
O	4.590310	-0.780643	1.622051
O	4.279986	-2.134020	-0.487477
C	3.675364	0.411682	-0.530872
F	4.906063	0.784019	-0.867458
F	3.114977	1.386311	0.168470
F	2.977360	0.208623	-1.639196
P	-1.036475	1.121530	-0.122964
O	-1.841352	1.538063	1.202210
O	-0.447711	2.566445	-0.534442
C	-2.664426	2.726103	1.221134
H	-2.012146	3.588700	1.363590
H	-3.170469	2.832395	0.257461
C	-3.658317	2.582397	2.349875
H	-4.322998	1.734076	2.176234
H	-3.137597	2.432313	3.296631
H	-4.265133	3.486109	2.425473
C	0.406291	2.649782	-1.692228
H	-0.224031	2.681613	-2.583344
H	1.034477	1.755280	-1.737066
C	1.254150	3.894106	-1.561882
H	0.622984	4.780271	-1.480556
H	1.886688	3.827427	-0.675700
H	1.893021	4.000979	-2.440197
H	2.062293	-1.011052	3.048927
O	-1.932938	0.582588	-1.212112
N	-3.430053	-1.526174	-0.419768
C	-3.546979	-1.523053	1.079258
H	-3.653899	-0.477407	1.364926
H	-4.471936	-2.050241	1.322977
C	-4.718294	-1.104461	-1.065267
H	-4.506523	-0.995611	-2.127021
H	-5.427690	-1.923940	-0.930497
C	-2.929539	-2.827757	-0.964535
H	-1.973963	-3.017535	-0.478383
H	-3.634987	-3.602398	-0.655855
C	-2.739815	-2.790106	-2.472618

H -2.152375 -3.659062 -2.768911
H -3.684252 -2.824071 -3.014139
H -2.192618 -1.891830 -2.771052
C -2.358180 -2.128782 1.806444
H -2.475259 -1.907027 2.868045
H -2.312476 -3.211955 1.696163
H -1.414155 -1.690304 1.475688
C -5.247017 0.208993 -0.512731
H -6.052381 0.555442 -1.160309
H -5.648375 0.106599 0.495055
H -4.458163 0.964689 -0.517782

13

Symbol	X	Y	Z
C	0.914777	-2.528344	1.217542
C	0.132363	-1.587782	0.553543
C	0.046174	-0.309831	1.092946
C	0.746143	-0.016721	2.260522
C	1.496796	-1.031370	2.841436
N	1.579232	-2.266519	2.341811
H	-3.201343	-0.426883	-0.840911
H	1.009847	-3.533610	0.822053
H	-0.381594	-1.845529	-0.364355
H	0.711865	0.967554	2.710821
S	3.576925	-0.794942	0.214504
O	4.487040	-0.591454	1.397736
O	3.793867	-2.123537	-0.462810
C	4.463021	0.310674	-1.024710
F	5.717210	-0.079832	-1.257917
F	4.504135	1.579276	-0.588760
F	3.814504	0.322249	-2.203097
P	-0.905122	0.946431	0.236752
O	-1.710910	1.657526	1.397992
O	0.104708	2.078093	-0.238066
C	-2.590992	2.778732	1.105582
H	-1.966807	3.636987	0.853137
H	-3.208865	2.517870	0.243147
C	-3.428240	3.029760	2.335223
H	-4.036295	2.155019	2.569620
H	-2.790029	3.258008	3.189416
H	-4.090146	3.878436	2.157395
C	1.137663	1.739139	-1.204379
H	0.660554	1.310675	-2.088264
H	1.781425	0.986074	-0.744294
C	1.894924	3.001571	-1.532453
H	1.225727	3.743805	-1.968839
H	2.353626	3.413495	-0.633095
H	2.684904	2.771094	-2.247873
H	2.058759	-0.840385	3.748894
O	-1.735709	0.384733	-0.869515
N	-4.036899	-1.054648	-0.856613
C	-4.868512	-0.738242	0.352499
H	-5.120953	0.318061	0.282277
H	-5.788423	-1.319941	0.263354
C	-4.776682	-0.766236	-2.129474
H	-4.087826	-0.985757	-2.941728
H	-5.610846	-1.468915	-2.180120
C	-3.493025	-2.452987	-0.818648
H	-2.898947	-2.521534	0.091180
H	-4.345360	-3.129064	-0.726833
C	-2.625098	-2.782020	-2.022881
H	-2.062066	-3.689237	-1.802054
H	-3.209831	-2.964574	-2.923228
H	-1.912433	-1.976608	-2.218134
C	-4.157430	-1.006702	1.668352
H	-4.770156	-0.593242	2.470088
H	-4.025648	-2.069012	1.867720
H	-3.186278	-0.507654	1.706724
C	-5.232834	0.679722	-2.228520
H	-5.562443	0.864899	-3.250756
H	-6.065167	0.906778	-1.564169
H	-4.404454	1.359684	-2.015129

14

Symbol	X	Y	Z
H	0.827737	0.020185	-0.282574
S	-1.406506	1.203360	-0.638083
O	-0.330341	0.486982	-1.447075
O	-0.830305	1.697705	0.660985
C	-2.292199	-0.333144	-0.025781
F	-2.932511	-0.944890	-1.023431
F	-3.181789	-0.035798	0.921342
F	-1.414893	-1.203097	0.492084
N	1.753007	-0.221306	0.152582
C	2.357309	1.072858	0.607467
H	1.634608	1.503945	1.296616
H	3.275108	0.827339	1.146862
C	1.540854	-1.173561	1.292108

H	0.922846	-1.980009	0.904091
H	2.522391	-1.580165	1.547326
C	2.518822	-0.855048	-0.967652
H	2.527678	-0.127029	-1.775890
H	3.542292	-1.008567	-0.618585
C	1.872901	-2.142005	-1.454634
H	2.338211	-2.426059	-2.398558
H	2.002778	-2.967223	-0.755474
H	0.806990	-1.982411	-1.633880
C	2.605571	2.046096	-0.532817
H	2.822484	3.024661	-0.103925
H	3.452587	1.758061	-1.154836
H	1.713502	2.134218	-1.155824
C	0.865497	-0.538068	2.497421
H	0.535198	-1.337875	3.160973
H	1.546263	0.102916	3.057339
H	-0.002243	0.047192	2.189700

15

Symbol	X	Y	Z
C	-2.942284	-1.530951	-0.742490
C	-1.612844	-1.464342	-0.339253
C	-1.158165	-0.280030	0.233100
C	-2.047191	0.783334	0.371687
C	-3.355590	0.608767	-0.065449
N	-3.804953	-0.522010	-0.611728
H	-3.327787	-2.439227	-1.193579
H	-0.952633	-2.310704	-0.478547
H	-1.735513	1.727742	0.802198
P	0.524774	-0.118012	0.856199
O	0.726915	-0.429777	2.285224
O	1.326359	-1.026491	-0.181706
O	0.954461	1.389028	0.542997
C	1.069628	1.859260	-0.821451
H	0.208333	1.509824	-1.397891
H	1.975084	1.429948	-1.255705
C	1.127702	3.367407	-0.782359
H	1.238049	3.757548	-1.795085
H	0.212102	3.772319	-0.349170
H	1.978014	3.698851	-0.185331
C	2.747618	-1.240745	0.015651
H	2.876937	-1.905158	0.870998
H	3.221459	-0.280991	0.240473
C	3.298091	-1.842204	-1.254707
H	4.366657	-2.029027	-1.139213
H	2.800410	-2.787821	-1.472916
H	3.150338	-1.162255	-2.094713
H	-4.072059	1.418140	0.027097

16

Symbol	X	Y	Z
C	-1.416470	0.831308	-1.083690
C	-0.273188	0.161161	-1.066675
C	0.243025	-0.521834	0.174425
C	-0.497631	-0.055322	1.403584
C	-1.639447	0.618194	1.333496
N	-2.198655	0.980637	0.087369
H	1.323099	-0.363800	0.275391
H	-1.781025	1.361624	-1.951330
H	0.339525	0.157544	-1.956185
H	-0.058916	-0.213514	2.379958
S	-3.690934	1.673799	0.009285
O	-0.191104	3.653043	-0.226971
O	-4.032011	2.194861	1.305709
O	-3.790788	2.437747	-1.204829
C	-4.760859	0.179601	-0.243933
F	-6.016586	0.550353	-0.382482
F	-4.640788	-0.619898	0.804685
F	-4.355098	-0.457290	-1.329936
S	1.147998	3.065853	-0.315693
O	1.468017	2.089294	0.728405
O	1.581601	2.698233	-1.665233
C	2.267013	4.471236	0.105865
F	2.135401	5.466242	-0.770339
F	3.539615	4.072613	0.093819
F	1.993435	4.947645	1.319521
N	3.539023	-1.251495	0.001173
C	3.643206	-0.057992	-0.859629
H	3.085500	0.748064	-0.379014
H	4.696583	0.269354	-0.923352
C	4.225480	-1.001797	1.275353
H	4.096201	-1.880715	1.909841
H	5.311282	-0.896337	1.100774
C	4.124164	-2.420209	-0.663145
H	3.695404	-2.481243	-1.662178
H	5.213357	-2.282570	-0.787203
C	3.845991	-3.742691	0.040257
H	4.244156	-4.565220	-0.558104

H	4.307898	-3.807743	1.026597
H	2.768677	-3.895034	0.145934
C	3.083703	-0.254233	-2.267151
H	2.802475	0.717864	-2.674179
H	3.803010	-0.720880	-2.942308
H	2.195070	-0.889540	-2.240353
C	3.721591	0.228081	2.024778
H	4.121046	0.221088	3.040558
H	4.028469	1.158175	1.545978
H	2.630113	0.255597	2.081621
P	0.038890	-2.305082	-0.011021
O	-1.452510	-2.706400	-0.270251
O	0.818692	-2.734882	-1.292016
O	0.465812	-3.119734	1.252525
C	-2.431968	-3.039320	0.768202
H	-1.962222	-3.740725	1.457146
H	-2.676677	-2.118017	1.295898
C	-3.628754	-3.634897	0.072313
H	-3.353140	-4.554651	-0.443967
H	-4.393455	-3.864751	0.815834
H	-4.043996	-2.930635	-0.648075
C	1.352926	-2.732435	2.350676
H	1.729672	-1.726988	2.161378
H	2.188144	-3.428006	2.310357
C	0.579963	-2.847147	3.640665
H	1.234673	-2.584907	4.473192
H	-0.280968	-2.176955	3.647250
H	0.230012	-3.870183	3.780792
C	0.627072	-4.050144	-1.916708
H	-0.386012	-4.069653	-2.316259
H	0.730222	-4.810982	-1.140622
C	1.671965	-4.194780	-2.992442
H	1.493392	-5.123915	-3.535563
H	1.612242	-3.362994	-3.695414
H	2.672269	-4.233266	-2.561510
H	-2.167216	0.986770	2.201581

17

Symbol	X	Y	Z
C	0.414320	0.602212	-1.086910
C	-0.770493	-0.024593	-1.054210
C	-1.570517	-0.047568	0.155445
C	-0.922209	0.423965	1.366527
C	0.259788	1.052709	1.318608
N	0.801593	1.435288	0.032658
H	-0.067642	-2.178887	-0.406799
H	1.149280	0.525231	-1.872376
H	-1.081070	-0.608516	-1.917507
H	-1.329462	0.167842	2.340685
S	0.881085	3.053701	-0.248907
O	4.420234	1.701173	0.411052
O	1.447113	3.700829	0.911820
O	1.405974	3.271581	-1.577488
C	-0.873955	3.706310	-0.317137
F	-0.868571	4.912313	-0.864081
F	-1.380116	3.789988	0.905399
F	-1.642350	2.906630	-1.045097
S	3.888394	0.363356	0.152398
O	3.068418	-0.204171	1.225641
O	3.390258	0.141135	-1.206725
C	5.379502	-0.721840	0.193634
F	6.261502	-0.364171	-0.737933
F	5.027984	-1.991019	-0.033836
F	5.982125	-0.666995	1.380304
N	0.471872	-3.055689	-0.363815
C	1.882093	-2.713407	-0.786303
H	2.153622	-1.820817	-0.225033
H	2.505867	-3.547652	-0.458001
C	0.435137	-3.525802	1.065090
H	-0.616196	-3.617396	1.329378
H	0.877603	-4.523840	1.066807
C	-0.180676	-4.047614	-1.286985
H	-0.180110	-3.596742	-2.276577
H	0.473795	-4.921267	-1.304051
C	-1.599924	-4.402909	-0.880906
H	-2.046185	-4.977956	-1.692836
H	-1.640552	-5.014268	0.019777
H	-2.204899	-3.502925	-0.740420
C	2.038467	-2.458633	-2.272609
H	3.032146	-2.035734	-2.416455
H	1.950624	-3.366975	-2.867945
H	1.319607	-1.718795	-2.630245
C	1.148290	-2.582279	2.014565
H	0.903346	-2.881128	3.034612
H	2.229822	-2.608019	1.894642
H	0.826494	-1.549109	1.866596
P	-3.213355	-0.409860	0.135605
O	-4.268154	0.742978	-0.160467

O	-3.506965	-1.447433	-1.013703
O	-3.718209	-0.926204	1.550715
C	-4.286124	1.931106	0.676210
H	-4.879529	1.700504	1.561658
H	-3.262343	2.160404	0.981676
C	-4.879075	3.060488	-0.130087
H	-5.893062	2.814809	-0.447507
H	-4.914328	3.962411	0.483153
H	-4.267395	3.259631	-1.010394
C	-3.141495	-2.099322	2.183246
H	-2.053215	-2.003652	2.167833
H	-3.433361	-2.974348	1.597996
C	-3.673579	-2.164645	3.593555
H	-3.259427	-3.039177	4.097103
H	-3.385485	-1.271482	4.149275
H	-4.760748	-2.245994	3.587992
C	-4.853824	-1.713376	-1.510515
H	-5.289399	-0.765756	-1.824278
H	-5.437483	-2.124961	-0.685157
C	-4.729748	-2.687422	-2.655641
H	-5.723797	-2.905136	-3.048537
H	-4.124225	-2.259960	-3.455617
H	-4.273250	-3.621233	-2.326696
H	0.885004	1.264577	2.172384

18

Symbol	X	Y	Z
C	0.777870	1.464120	-0.536922
C	2.114294	1.531276	-0.470799
C	2.853245	0.643417	0.404438
C	2.092694	-0.029127	1.435355
C	0.753544	-0.086330	1.363230
N	0.119450	0.365194	0.143116
H	-3.342396	-1.214580	0.759960
H	0.116406	2.175573	-1.008714
H	2.632274	2.334623	-0.983829
H	2.585672	-0.402742	2.328395
S	-0.554329	-0.792001	-0.797956
O	-3.108196	0.667663	0.206518
O	-1.331512	-1.687030	0.040859
O	-1.112885	-0.190090	-1.986866
C	0.802515	-1.910200	-1.442099
F	0.289595	-2.729316	-2.350842
F	1.316510	-2.634345	-0.456680
F	1.767964	-1.196307	-2.003701
S	-3.335992	1.756086	-0.754248
O	-2.208858	2.668613	-0.926406
O	-4.036312	1.363693	-1.975580
C	-4.591436	2.789872	0.121184
F	-5.752057	2.136077	0.212023
F	-4.809331	3.927006	-0.534878
F	-4.187420	3.087958	1.354523
N	-4.113608	-1.889586	0.892417
C	-4.079066	-2.802515	-0.300054
H	-3.115247	-3.301990	-0.270407
H	-4.870648	-3.540453	-0.152411
C	-3.907295	-2.630534	2.184496
H	-3.771853	-1.869814	2.950265
H	-4.842312	-3.158295	2.384891
C	-5.384756	-1.085404	0.925301
H	-5.374900	-0.471835	0.027748
H	-6.204026	-1.804613	0.860378
C	-5.506159	-0.201405	2.154175
H	-6.356259	0.464365	2.007447
H	-5.680175	-0.772418	3.065362
H	-4.613505	0.416126	2.271540
C	-4.221222	-2.044714	-1.609024
H	-3.944383	-2.714888	-2.423602
H	-5.240833	-1.703712	-1.786824
H	-3.555115	-1.179762	-1.629882
C	-2.723181	-3.582026	2.186210
H	-2.578656	-3.925975	3.210919
H	-2.895268	-4.459258	1.563490
H	-1.812538	-3.081486	1.857154
P	4.520217	0.452377	0.282482
O	5.100873	-0.688945	-0.662092
O	5.171681	1.754288	-0.319251
O	5.176320	0.059261	1.674985
C	4.694199	-2.067162	-0.457740
H	5.352288	-2.494530	0.299662
H	3.666600	-2.079469	-0.085849
C	4.802275	-2.787969	-1.779506
H	5.824615	-2.745345	-2.156838
H	4.522919	-3.834404	-1.646396
H	4.132340	-2.337349	-2.512226
C	5.096364	0.949483	2.822576
H	4.111910	1.422477	2.836890
H	5.857468	1.720388	2.693030

C	5.340130	0.122276	4.061013
H	5.313093	0.767665	4.940062
H	4.572021	-0.644938	4.168061
H	6.317258	-0.359192	4.011614
C	6.531449	1.773593	-0.844435
H	6.548462	1.167087	-1.749404
H	7.194106	1.326358	-0.100693
C	6.884980	3.214797	-1.114243
H	7.896292	3.269327	-1.519762
H	6.194822	3.646481	-1.839824
H	6.845124	3.798093	-0.193870
H	0.096524	-0.419719	2.152700

19

Symbol	X	Y	Z
C	-0.259714	-0.659572	1.751263
C	-1.223802	-0.799248	0.753916
C	-2.507114	-0.346394	1.033850
C	-2.795128	0.205880	2.283334
C	-1.758636	0.275305	3.205176
N	-0.516610	-0.143256	2.951358
H	3.243359	1.225437	0.097527
H	0.761316	-0.961409	1.545824
H	-0.952261	-1.252861	-0.190196
H	-3.783600	0.567241	2.542045
S	0.777828	1.462157	-0.538875
O	2.446491	-0.874262	-0.361257
O	1.544241	1.601396	0.762367
O	1.557086	2.081102	-1.672165
C	-0.479475	2.820000	-0.222477
F	0.079993	4.019542	-0.071362
F	-1.190248	2.556544	0.886625
F	-1.355542	2.898955	-1.238029
S	2.168281	-2.221462	-0.866725
O	0.752851	-2.594642	-0.861723
O	2.913686	-2.604212	-2.063825
C	2.903510	-3.275878	0.456845
F	4.210794	-3.031320	0.566269
F	2.742262	-4.568450	0.188205
F	2.336190	-3.018602	1.634988
N	4.254881	1.452370	0.154735
C	4.764278	1.744020	-1.225383
H	4.066340	2.467194	-1.641762
H	5.744592	2.211582	-1.105381
C	4.319213	2.667045	1.036764
H	3.737648	2.417730	1.920982
H	5.366747	2.796551	1.318117
C	4.922557	0.276073	0.798098
H	4.819015	-0.551483	0.103768
H	5.979987	0.527409	0.908209
C	4.263362	-0.094293	2.117742
H	4.602056	-1.089519	2.407323
H	4.515745	0.596370	2.922164
H	3.178552	-0.123608	1.987143
C	4.824243	0.525482	-2.130138
H	4.997871	0.873592	-3.149220
H	5.636068	-0.153060	-1.867313
H	3.881679	-0.021283	-2.102952
C	3.744248	3.913530	0.384170
H	3.612758	4.671843	1.157070
H	4.403876	4.323753	-0.380576
H	2.771595	3.688259	-0.053848
P	-3.763284	-0.378554	-0.217385
O	-3.904077	0.955500	-1.009532
O	-3.351222	-1.474625	-1.234411
O	-5.163539	-0.614410	0.437513
C	-4.322463	2.220730	-0.390790
H	-5.232083	2.026543	0.178442
H	-3.518630	2.531255	0.276998
C	-4.549563	3.207390	-1.506107
H	-5.340102	2.858797	-2.170743
H	-4.851091	4.162869	-1.074442
H	-3.632934	3.354867	-2.075963
C	-5.488905	-1.798964	1.242297
H	-4.630130	-2.033164	1.874580
H	-5.662103	-2.618051	0.545508
C	-6.713207	-1.464017	2.053640
H	-6.994712	-2.334417	2.647521
H	-6.511248	-0.631482	2.728099
H	-7.544795	-1.203352	1.398974
C	-3.927593	-1.617229	-2.581563
H	-3.717736	-0.693738	-3.119195
H	-5.004241	-1.746131	-2.469362
C	-3.267642	-2.816003	-3.210162
H	-3.652757	-2.942264	-4.222852
H	-2.188237	-2.671894	-3.261413
H	-3.483929	-3.717972	-2.637876
H	-1.939168	0.687820	4.191658

1

Symbol	X	Y	Z
C	-3.173851	-0.949449	-1.048349
C	-4.472012	-1.411793	-1.222749
C	-5.405082	-1.158602	-0.224925
C	-5.007284	-0.454694	0.905948
C	-3.688464	-0.030117	0.991688
N	-2.791528	-0.269680	0.033111
H	-6.426088	-1.506084	-0.325252
H	-2.406329	-1.130866	-1.793698
H	-4.737458	-1.958045	-2.118027
H	-5.700156	-0.238677	1.708233
S	-0.270579	-0.051028	0.555593
O	1.377034	0.378262	0.856622
O	-0.193035	-1.110350	-0.400898
O	-0.729769	-0.133463	1.904234
C	-0.744813	1.607509	-0.255459
F	0.311822	2.396308	-0.296028
F	-1.673804	2.172137	0.472211
F	-1.152258	1.362583	-1.473586
S	2.552840	0.480674	-0.240084
O	1.989754	0.460849	-1.559887
O	3.451037	1.499563	0.207920
C	3.362703	-1.164270	0.072467
F	3.923583	-1.145495	1.261078
F	4.273781	-1.345608	-0.860963
F	2.454259	-2.113504	0.010299
H	-3.329248	0.518913	1.856066

20

Symbol	X	Y	Z
C	-0.559674	-1.898929	-2.202798
C	0.119610	-0.774294	-1.740416
C	1.023903	-0.949880	-0.701778
C	1.215625	-2.222921	-0.159830
C	0.489320	-3.271939	-0.706909
N	-0.379881	-3.121358	-1.709285
H	-1.295161	-1.790408	-2.990966
H	-0.086362	0.204514	-2.149882
H	1.892666	-2.408350	0.665548
O	-2.834009	0.101195	-1.738057
S	-2.712221	0.800290	-0.460318
O	-1.390601	1.389290	-0.203603
O	-3.842988	1.649584	-0.095919
C	-2.785584	-0.559593	0.786543
F	-3.922769	-1.238745	0.691241
F	-2.696747	-0.058932	2.021224
F	-1.771595	-1.413287	0.623076
P	1.914865	0.440518	-0.038531
O	3.450939	0.337688	-0.299252
O	1.430074	1.707162	-0.781254
O	1.773710	0.516044	1.513605
C	4.297827	-0.743299	0.208217
H	4.053423	-0.900596	1.259880
H	4.064048	-1.638790	-0.368774
C	5.729158	-0.310287	0.020590
H	5.932146	0.594945	0.592602
H	6.390014	-1.103795	0.371614
H	5.934874	-0.124421	-1.033566
C	0.500134	0.529441	2.258727
H	-0.310844	0.706520	1.552958
H	0.591383	1.385659	2.923735
C	0.344661	-0.764219	3.017786
H	-0.542646	-0.689999	3.648903
H	0.207362	-1.607716	2.341074
H	1.211914	-0.943208	3.654080
C	1.127527	3.008748	-0.168648
H	2.066606	3.422355	0.197242
H	0.438395	2.831442	0.655522
C	0.495082	3.852513	-1.242612
H	0.258227	4.833945	-0.828826
H	1.177528	3.983039	-2.082775
H	-0.424963	3.377041	-1.581123
H	0.610580	-4.276163	-0.316460

21

Symbol	X	Y	Z
C	1.918176	-3.778294	0.291130
C	2.205154	-2.432756	0.092436
C	1.206239	-1.510046	0.392447
C	-0.022796	-1.947909	0.876253
C	-0.194955	-3.319922	1.035049
N	0.746537	-4.219611	0.750757
H	2.667681	-4.530470	0.071947
H	3.177058	-2.127134	-0.274156
H	-0.823682	-1.254504	1.104527
P	1.500702	0.215202	0.080139

O 1.392812 0.633484 -1.421624
O 0.452218 0.958553 0.949673
O 2.973406 0.529804 0.467611
C 0.143617 0.674941 -2.199301
H -0.649767 1.058573 -1.554762
H 0.360529 1.391775 -2.986580
C -0.192575 -0.692367 -2.743020
H 0.646357 -1.099127 -3.309010
H -0.464980 -1.384626 -1.944695
H -1.052032 -0.597501 -3.409013
C 3.976867 1.236773 -0.341538
H 3.991171 0.773091 -1.326743
H 4.906763 1.023724 0.177868
C 3.686579 2.716210 -0.407668
H 4.525145 3.215566 -0.895816
H 2.789733 2.918572 -0.995290
H 3.575300 3.133362 0.594162
C 0.132248 2.391246 0.876411
H -0.921054 2.418018 0.601389
H 0.728790 2.847366 0.084280
C 0.403132 3.007585 2.225117
H -0.191865 2.512133 2.992540
H 1.459564 2.929781 2.484701
H 0.126025 4.062459 2.195632
I -3.199390 0.333522 -0.038234
H -1.136955 -3.705987 1.408880

22

Symbol	X	Y	Z
C	3.031562	-3.165385	-0.601999
C	2.511050	-1.920482	-0.944099
C	2.133565	-1.062411	0.082387
C	2.289919	-1.478597	1.402412
C	2.816403	-2.743773	1.631649
N	3.184166	-3.578330	0.656182
H	3.334719	-3.859952	-1.378406
H	2.397792	-1.631956	-1.981884
H	2.001967	-0.844421	2.231749
P	1.489893	0.569769	-0.319777
O	1.159901	0.749884	-1.751491
O	0.297278	0.708642	0.724279
O	2.551460	1.628422	0.238111
C	-1.988018	-0.365938	-1.394842
H	-1.404540	0.542616	-1.274528
H	-2.508791	-0.348195	-2.348065
C	-1.154984	-1.614469	-1.201995
H	-0.352246	-1.612203	-1.945099
H	-0.700694	-1.632949	-0.210076
H	-1.751106	-2.518091	-1.332332
C	3.373901	2.420247	-0.654030
H	3.639696	1.822665	-1.527694
H	4.276937	2.635845	-0.086148
C	2.645911	3.686304	-1.050490
H	3.302275	4.318571	-1.651279
H	1.759597	3.446531	-1.640211
H	2.345576	4.243562	-0.161062
C	-0.291461	2.011380	0.987236
H	-1.368454	1.847495	0.989109
H	-0.046392	2.694304	0.169190
C	0.202067	2.534300	2.317279
H	-0.036428	1.824972	3.111202
H	1.281010	2.688523	2.288231
H	-0.285779	3.484444	2.542379
I	-3.565260	-0.226413	0.087411
H	2.947481	-3.102749	2.647262

2

Symbol	X	Y	Z
C	-2.926496	-1.185870	-0.907730
C	-4.230406	-1.497500	-1.219772
C	-5.249192	-1.076573	-0.370370
C	-4.944823	-0.354790	0.780135
C	-3.629545	-0.057816	1.056719
N	-2.664968	-0.474372	0.209844
H	-6.279117	-1.315762	-0.601715
H	-2.076434	-1.490885	-1.503185
H	-4.435363	-2.066845	-2.114744
H	-5.713876	-0.022595	1.462248
S	-0.930927	-0.090736	0.598469
O	1.732282	0.528092	1.095804
O	-0.226464	-1.152848	-0.041080
O	-0.961421	0.225341	1.991244
C	-0.908540	1.514443	-0.425842
F	0.077395	2.287989	-0.085277
F	-2.054910	2.119183	-0.169569
F	-0.853891	1.165900	-1.686725
S	2.631927	0.556189	-0.066646
O	1.940494	0.530021	-1.355268

O 3.738066 1.504909 0.037214
C 3.467444 -1.084771 0.014212
F 4.146310 -1.205750 1.153781
F 4.323416 -1.219998 -0.997711
F 2.583430 -2.074756 -0.052285
H -3.306497 0.489482 1.931960

3

Symbol	X	Y	Z
C	0.491890	-0.646278	1.427513
C	0.066920	-1.950780	1.326721
C	-0.331211	-2.443155	0.087553
C	-0.281980	-1.626472	-1.038262
C	0.179861	-0.338901	-0.907874
N	0.521638	0.122100	0.317369
H	-0.667943	-3.467854	-0.003182
H	0.840599	-0.187568	2.342536
H	0.058920	-2.568921	2.213058
H	-0.571017	-1.986801	-2.014995
S	1.168655	1.773864	0.444007
O	2.754029	-1.556557	-0.769816
O	1.483593	1.997680	1.818970
O	2.024178	1.974449	-0.681705
C	-0.434321	2.691857	0.108957
F	-0.257002	3.938005	0.469212
F	-1.398780	2.138106	0.810916
F	-0.705701	2.613817	-1.178683
S	3.719759	-1.170941	0.261319
O	3.260438	-0.073716	1.125378
O	4.366500	-2.275463	0.964138
C	5.091401	-0.402917	-0.700861
F	5.632644	-1.296249	-1.526653
F	6.042581	0.042177	0.116836
F	4.646595	0.619544	-1.423747
P	-3.571170	-1.776990	-0.160473
O	-2.906299	-0.637968	0.848126
O	-3.303054	-0.972523	-1.554198
O	-5.157133	-1.573036	0.154579
C	-3.081580	-0.862016	2.256965
H	-4.136513	-1.067156	2.462808
H	-2.500789	-1.741055	2.557985
C	-2.634765	0.373513	3.003609
H	-3.210869	1.240999	2.677587
H	-2.786126	0.233353	4.074945
H	-1.577452	0.577944	2.830850
C	-5.825118	-0.295541	0.179661
H	-5.093168	0.495436	0.363721
H	-6.507237	-0.325969	1.030060
C	-6.583867	-0.080684	-1.115243
H	-7.091127	0.885877	-1.098259
H	-5.906508	-0.107409	-1.971078
H	-7.332130	-0.863645	-1.245496
C	-3.085266	0.440525	-1.715032
H	-2.358080	0.765334	-0.971537
H	-4.015146	0.984217	-1.532231
C	-2.597252	0.672899	-3.126938
H	-2.396198	1.734202	-3.282987
H	-1.680326	0.111344	-3.317460
H	-3.350197	0.351211	-3.847802
H	0.313461	0.336026	-1.743532

4

Symbol	X	Y	Z
C	0.162481	-0.696428	1.173237
C	-0.662321	-1.723754	0.990363
C	-1.468150	-1.831761	-0.282658
C	-0.723983	-1.192758	-1.431932
C	0.135041	-0.207252	-1.205928
N	0.384581	0.217967	0.119553
H	-1.723351	-2.869190	-0.507932
H	0.761729	-0.537479	2.057474
H	-0.761883	-2.481834	1.754625
H	-0.874344	-1.566138	-2.434621
S	1.185301	1.643440	0.380712
O	2.703265	-1.395631	-1.049943
O	1.378575	1.809220	1.796801
O	2.205580	1.857662	-0.609327
C	-0.158960	2.842337	-0.082325
F	0.291012	4.070642	0.090069
F	-1.221273	2.645503	0.678154
F	-0.495690	2.666246	-1.352900
S	3.514255	-1.419262	0.166794
O	3.111202	-0.454411	1.195078
O	3.840299	-2.752927	0.673394
C	5.145245	-0.768627	-0.395118
F	5.661446	-1.554454	-1.340450
F	6.006564	-0.721641	0.620774
F	5.024620	0.457449	-0.895353

P	-3.068120	-0.987315	-0.070370
O	-2.972615	0.200501	0.947388
O	-3.572296	-0.363249	-1.404791
O	-4.083533	-2.066323	0.417350
C	-3.071726	0.032289	2.405488
H	-4.133965	0.064494	2.646677
H	-2.668221	-0.943777	2.675422
C	-2.307165	1.154844	3.054764
H	-2.712329	2.120350	2.752498
H	-2.397535	1.059222	4.137713
H	-1.250979	1.112381	2.785715
C	-5.520424	-1.780796	0.559830
H	-5.656537	-0.700565	0.643778
H	-5.798704	-2.249934	1.500153
C	-6.259759	-2.357045	-0.622146
H	-7.330061	-2.195541	-0.485027
H	-5.947976	-1.870769	-1.547015
H	-6.075390	-3.428675	-0.698028
C	-3.589140	1.063790	-1.767281
H	-3.016940	1.615363	-1.022599
H	-4.634038	1.363195	-1.713914
C	-3.017680	1.203886	-3.154273
H	-3.068918	2.253207	-3.450050
H	-1.975126	0.886182	-3.176674
H	-3.590828	0.612353	-3.868333
H	0.727567	0.269462	-1.973643

5

Symbol	X	Y	Z
C	-0.362483	0.737922	-1.258399
C	0.527194	-0.197207	-0.941872
C	1.114081	-0.284713	0.442861
C	0.211288	0.391439	1.444690
C	-0.663777	1.317993	1.074201
N	-0.826522	1.658893	-0.289284
H	1.302621	-1.330404	0.709129
H	-0.817113	0.823372	-2.234787
H	0.817208	-0.936694	-1.676895
H	0.267015	0.101099	2.484434
S	-2.033086	2.729963	-0.696587
O	-3.108511	-0.485028	2.189813
O	-2.512479	2.436893	-2.020642
O	-2.892993	2.961188	0.433439
C	-1.016910	4.273690	-0.848166
F	-1.817121	5.293532	-1.092639
F	-0.151406	4.137155	-1.836813
F	-0.368048	4.477421	0.286166
S	-3.020493	-1.033907	0.841555
O	-3.101075	-0.064816	-0.251327
O	-1.984808	-2.066889	0.667552
C	-4.582024	-2.000653	0.660523
F	-4.654403	-2.953444	1.585957
F	-4.633961	-2.578595	-0.539711
F	-5.640539	-1.206696	0.789147
P	2.741334	0.524997	0.515810
O	2.861846	1.690952	-0.526164
O	2.988753	1.185877	1.907991
O	3.827013	-0.565111	0.281451
C	3.226145	1.484982	-1.935189
H	4.311182	1.572832	-1.983777
H	2.931970	0.478156	-2.234825
C	2.529615	2.540578	-2.751273
H	2.783224	3.535697	-2.384892
H	2.853169	2.456151	-3.789720
H	1.449210	2.407287	-2.708127
C	5.267035	-0.293542	0.416544
H	5.431072	0.782787	0.334518
H	5.717576	-0.790473	-0.438951
C	5.750955	-0.844928	1.734477
H	6.829315	-0.695633	1.807937
H	5.268435	-0.332236	2.567088
H	5.540980	-1.912748	1.797653
C	2.688993	2.588058	2.239804
H	1.866900	2.928163	1.609230
H	3.587071	3.155799	2.003365
C	2.338001	2.636733	3.703444
H	2.158005	3.673627	3.990917
H	1.436012	2.056935	3.901356
H	3.157283	2.244721	4.306423
H	-1.336388	1.807800	1.763092
I	1.687062	-3.977270	-0.840471
Na	-1.349768	-3.384183	-1.035056

6

Symbol	X	Y	Z
C	1.464617	-1.223626	-1.705894
C	0.212552	-0.800762	-1.844404
C	-0.683627	-0.613602	-0.644383

C	-0.213519	-1.436983	0.527816
C	1.042564	-1.852473	0.619593
N	1.957712	-1.622864	-0.440218
H	-1.731415	-0.848035	-0.865671
H	2.149058	-1.363361	-2.530826
H	-0.167921	-0.586250	-2.834119
H	-0.928709	-1.684573	1.300568
S	3.579960	-1.761703	-0.177764
O	3.790937	-2.605286	0.967313
O	4.237094	-1.959043	-1.441212
C	4.009619	-0.039410	0.367502
F	5.273324	0.005387	0.734282
F	3.232491	0.302677	1.379505
F	3.805500	0.788326	-0.645227
P	-0.663734	1.134341	-0.183312
O	-1.418720	2.039154	-1.209549
O	-1.267180	1.219062	1.248682
O	0.798867	1.673763	-0.146285
C	-2.875445	2.010674	-1.423679
H	-3.349633	1.566803	-0.546391
H	-3.152377	3.058979	-1.499994
C	-3.186290	1.241239	-2.682903
H	-2.649795	1.662854	-3.533411
H	-2.931296	0.185153	-2.577083
H	-4.258425	1.305901	-2.876790
C	1.426445	2.630709	-1.068901
H	0.638528	3.145198	-1.615659
H	2.022562	2.034328	-1.756723
C	2.258933	3.576288	-0.240958
H	2.788069	4.259591	-0.907132
H	1.626763	4.161413	0.427913
H	2.992962	3.028294	0.349269
C	-1.064627	2.359415	2.152542
H	-1.998595	2.430845	2.703521
H	-0.935183	3.262261	1.553828
C	0.118380	2.080555	3.048061
H	-0.039260	1.154578	3.601896
H	1.037087	1.998818	2.465760
H	0.226772	2.899525	3.760981
I	-4.316656	-1.266083	0.404140
H	1.428797	-2.438622	1.441064

7

Symbol	X	Y	Z
C	-2.255751	0.013622	-1.986741
C	-1.453651	1.051057	-2.194954
C	-1.404724	2.210409	-1.228895
C	-2.657438	2.280933	-0.389264
C	-3.438133	1.221073	-0.223530
N	-3.178434	0.012417	-0.913942
H	-1.236230	3.156404	-1.753324
H	-2.312368	-0.843114	-2.643110
H	-0.817053	1.062615	-3.068996
H	-2.916966	3.214867	0.088646
S	-3.898293	-1.392914	-0.430321
O	-5.098365	-1.070235	0.292372
O	-3.852872	-2.330593	-1.518843
C	-2.680526	-2.001469	0.831498
F	-3.097238	-3.144698	1.333709
F	-2.569126	-1.099043	1.789986
F	-1.507468	-2.167407	0.242539
P	0.026022	1.987942	-0.148594
O	1.381399	1.947307	-0.927062
O	-0.015716	3.183420	0.855753
C	1.983481	3.099515	-1.610243
H	1.536404	3.148554	-2.602595
H	1.734523	4.001527	-1.048306
C	3.469570	2.856211	-1.663702
H	3.884670	2.795374	-0.657399
H	3.684416	1.930142	-2.197011
H	3.946454	3.683281	-2.191392
C	0.841594	3.248215	2.048030
H	1.853464	2.957481	1.758957
H	0.442417	2.530335	2.763874
C	0.787991	4.664508	2.558344
H	1.175601	5.356011	1.809974
H	-0.236350	4.940509	2.808747
H	1.400898	4.741445	3.457275
H	-4.338269	1.225134	0.374475
O	-0.081746	0.624465	0.587839
C	0.967349	-0.416359	0.677196
H	0.714395	-1.155248	-0.079635
H	1.925998	0.033792	0.430890
C	0.940379	-0.974671	2.074709
H	-0.041237	-1.381767	2.313988
H	1.681227	-1.773437	2.134664
H	1.196354	-0.205344	2.804423
I	4.268964	-2.107374	-0.131392

8

Symbol	X	Y	Z
C	1.160449	0.444748	-1.628352
C	-0.021844	-0.116699	-1.847896
C	-0.245948	-1.589288	-1.613694
C	1.048521	-2.350293	-1.751616
C	2.218517	-1.761032	-1.540833
N	2.288402	-0.359086	-1.314324
H	-1.005714	-1.984110	-2.293024
H	1.365562	1.499386	-1.749190
H	-0.845415	0.505224	-2.172467
H	1.022697	-3.401323	-2.005143
S	3.594696	0.301284	-0.571010
O	4.724588	-0.570716	-0.762290
O	3.632225	1.709761	-0.869670
C	3.154373	0.180153	1.228522
F	4.122694	0.729511	1.942202
F	3.022105	-1.087592	1.560341
F	2.025668	0.832085	1.440844
P	-0.878941	-1.795104	0.085041
O	-2.193063	-0.890287	0.209395
O	-1.394010	-3.308432	0.047088
C	-3.316240	-1.032059	-0.688786
H	-3.117267	-0.425347	-1.576381
H	-3.414549	-2.080137	-0.984387
C	-4.549425	-0.548382	0.037956
H	-4.737182	-1.163051	0.919414
H	-4.417195	0.489170	0.348667
H	-5.415479	-0.608284	-0.623072
C	-1.951584	-3.898592	1.248520
H	-2.685275	-3.206587	1.670908
H	-1.143559	-4.039133	1.968081
C	-2.592504	-5.208986	0.859748
H	-3.392963	-5.042688	0.137621
H	-1.851816	-5.877946	0.419951
H	-3.013717	-5.688696	1.744475
H	3.174072	-2.261641	-1.601461
O	0.075513	-1.453988	1.160544
C	-1.072320	1.773972	1.324528
H	-0.413748	1.053602	0.847445
H	-1.865983	1.241894	1.841244
C	-0.333540	2.759553	2.203768
H	0.154209	2.204931	3.010762
H	0.437361	3.291218	1.644544
H	-1.010445	3.489107	2.649308
I	-2.084158	2.765070	-0.318843

9

Symbol	X	Y	Z
C	-1.112965	1.802689	0.629538
C	0.198556	1.919253	0.791272
C	1.040272	0.767659	1.281418
C	0.201081	-0.220354	2.050782
C	-1.110471	-0.306564	1.872958
N	-1.789771	0.622181	1.038186
H	1.869413	1.125714	1.898406
H	-1.753441	2.591811	0.262558
H	0.674952	2.859900	0.547901
H	0.683850	-0.888797	2.750609
S	-3.251760	0.240999	0.394996
O	-3.891061	-0.728332	1.246748
O	-3.897825	1.454568	-0.033672
C	-2.793760	-0.668940	-1.157369
F	-3.899776	-1.042327	-1.778245
F	-2.081823	-1.732635	-0.839968
F	-2.095444	0.132765	-1.937618
P	1.779412	-0.069378	-0.159900
O	2.719481	1.004369	-0.886957
O	2.772735	-1.094189	0.562552
C	3.836590	1.624363	-0.209408
H	3.450962	2.400802	0.456232
H	4.354610	0.871908	0.390292
C	4.744837	2.210964	-1.264266
H	5.119938	1.424769	-1.920671
H	4.205364	2.945024	-1.863732
H	5.592738	2.704579	-0.787256
C	3.554736	-2.012937	-0.240814
H	4.066434	-1.446391	-1.024248
H	2.873724	-2.725435	-0.708910
C	4.539783	-2.696707	0.676517
H	5.207007	-1.965188	1.134388
H	4.012138	-3.236381	1.463833
H	5.139436	-3.408429	0.107247
H	-1.749667	-1.010671	2.385780
O	0.824088	-0.655240	-1.121477

IEt

Symbol	X	Y	Z
C	-1.400790	0.701238	-0.000033
H	-1.464466	1.322623	-0.888971
H	-1.464945	1.322173	0.889202
C	-2.405353	-0.431907	-0.000036
H	-3.414494	-0.010238	-0.002578
H	-2.294793	-1.059808	-0.884372
H	-2.297691	-1.056406	0.887111
I	0.637231	-0.040270	0.000000

NaI

Symbol	X	Y	Z
Na	0. 0.000000	0.000000	-2.555256
I	0. 0.000000	0.000000	0.530336

NEt_3

Symbol	X	Y	Z
N	0. -0.000194	-0.000039	-0.174926
C	0. -0.668918	-1.219221	0.286164
H	-0.082329	-2.070331	-0.060388
H	-0.676116	-1.262209	1.391466
C	1.390077	0.030504	0.286096
H	1.833577	0.964370	-0.060119
H	1.430575	0.045741	1.391415
C	-0.721193	1.188567	0.287046
H	-1.752165	1.105842	-0.057713
H	-0.752855	1.216300	1.392397
C	-0.151283	2.499206	-0.247175
H	-0.865844	3.306425	-0.077376
H	0.783994	2.781601	0.236997
H	0.025808	2.417586	-1.322227
C	-2.089422	-1.380345	-0.246942
H	-2.430686	-2.403266	-0.078908
H	-2.801499	-0.712973	0.239326
H	-2.108474	-1.183791	-1.321568
C	2.240757	-1.118708	-0.246688
H	3.297012	-0.901832	-0.078357
H	2.019598	-2.069140	0.239851
H	2.080652	-1.234063	-1.321328

o-10

Symbol	X	Y	Z
C	0. -0.660992	-0.074439	-0.763016
C	0. -0.748179	0.041053	-2.263668
C	-1.689771	0.794712	-2.834513
C	-2.621409	1.571253	-2.019682
C	-2.390381	1.741309	-0.718110
N	-1.274864	1.099185	-0.109124
H	-1.759192	0.860441	-3.912988
H	0.388738	-0.168464	-0.447797
H	-0.034354	-0.528534	-2.844028
H	-3.491061	2.031299	-2.467315
S	-0.482480	1.877345	1.094549
O	-1.425369	2.679310	1.832234
O	0.424304	0.956084	1.730352
C	0.606237	3.077476	0.183628
F	-0.137716	4.015339	-0.373637
F	1.266925	2.420853	-0.761393
F	1.467295	3.623716	1.020926
P	-1.442030	-1.621026	-0.163873
O	-1.324720	-1.581462	1.428656
O	-2.987082	-1.335237	-0.385901
C	-0.086926	-2.013938	2.050355
H	0.045936	-3.077254	1.841778
H	0.737998	-1.454803	1.602922
C	-0.196902	-1.733210	3.528621
H	-1.042627	-2.271452	3.959255
H	0.715487	-2.059122	4.031316
H	-0.326976	-0.663756	3.695933
C	-3.986999	-2.269571	0.093306
H	-3.924424	-2.305896	1.182328
H	-3.762406	-3.258530	-0.311651
C	-5.332672	-1.769741	-0.374171
H	-6.116590	-2.442216	-0.022864
H	-5.524927	-0.771736	0.021770
H	-5.367000	-1.731313	-1.463573
O	-0.878138	-2.832306	-0.802844
H	-3.000450	2.333816	-0.051587
N	2.564387	-0.984279	-0.321082
C	2.617580	-2.428587	-0.099022
H	2.647402	-2.586064	0.983796
H	1.669969	-2.856479	-0.447667
C	2.669112	-0.551785	-1.710379
H	2.183372	-1.320500	-2.320953
H	2.073893	0.361946	-1.832510
C	3.251958	-0.157786	0.666315
H	3.178378	0.880085	0.325629
H	2.681229	-0.207652	1.600540

C	4.713181	-0.498014	0.985153
H	5.102567	0.220765	1.710436
H	4.792025	-1.491595	1.432347
H	5.350453	-0.470671	0.100553
C	3.767572	-3.204721	-0.752293
H	3.697630	-4.258503	-0.471425
H	3.708529	-3.150635	-1.841701
H	4.745884	-2.832728	-0.444264
C	4.066927	-0.259713	-2.269916
H	3.984211	0.011794	-3.325148
H	4.524707	0.583243	-1.747581
H	4.734540	-1.118001	-2.187465

o-11

Symbol	X	Y	Z
C	0. 0.874138	-0.617851	-0.654142
C	0. 0.585480	-0.884305	-2.014499
C	0.446351	-2.175246	-2.466416
C	0.917281	-3.226971	-1.606403
C	1.153826	-3.008746	-0.295880
N	0.717390	-1.740640	0.239585
H	0.207862	-2.383153	-3.500119
H	-1.304572	0.872616	0.046625
H	0.558259	-0.052846	-2.714975
H	1.203974	-4.180148	-2.037816
S	-0.676060	-1.784006	1.131007
O	-0.583638	-2.850311	2.104556
O	-1.012289	-0.423520	1.536454
C	-2.146140	-2.239508	0.073668
F	-2.025875	-3.448899	-0.437991
F	-2.274388	-1.351255	-0.910042
F	-3.240724	-2.189804	0.823225
P	1.982930	0.655305	-0.215748
O	2.293267	0.523492	1.357538
O	3.427189	0.258619	-0.822517
C	1.639726	1.356511	2.334283
H	1.777594	2.403454	2.053511
H	0.573297	1.123391	2.341485
C	2.267019	1.057803	3.676698
H	3.336141	1.274590	3.655015
H	1.801265	1.672012	4.449152
H	2.126688	0.007068	3.935204
C	4.532993	1.160481	-0.620583
H	4.674293	1.306823	0.453947
H	4.290036	2.123209	-1.075987
C	5.757826	0.541964	-1.255211
H	6.619006	1.199004	-1.122757
H	5.979912	-0.421366	-0.794013
H	5.596398	0.390444	-2.323487
O	1.571835	2.033080	-0.625755
H	1.658303	-3.682174	0.381609
N	-1.857522	1.698378	-0.226951
C	-1.261589	2.789788	0.630829
H	-1.653797	2.626879	1.635818
H	-0.192168	2.586620	0.626914
C	-1.589677	1.834626	-1.705121
H	-0.567976	2.211838	-1.779400
H	-1.593774	0.811557	-2.083900
C	-3.276263	1.325055	0.127868
H	-3.631417	0.679971	-0.675620
H	-3.192476	0.721222	1.032460
C	-4.229820	2.482950	0.366779
H	-5.206770	2.053628	0.592714
H	-3.929943	3.084507	1.224908
H	-4.341675	3.129540	-0.501852
C	-1.482825	4.214309	0.153605
H	-1.021330	4.873702	0.890237
H	-0.983279	4.391419	-0.799497
H	-2.531498	4.491697	0.066906
C	-2.589308	2.667676	-2.488393
H	-2.254289	2.680775	-3.526505
H	-3.584902	2.222910	-2.473413
H	-2.655352	3.698701	-2.146910

o-3

Symbol	X	Y	Z
C	0. 0.147291	-0.908176	0.935656
C	0. -0.164215	-2.228346	0.698441
C	-0.485647	-2.629229	-0.592650
C	-0.501559	-1.698281	-1.627627
C	-0.204727	-0.385270	-1.351094
N	0.129317	-0.032828	-0.090463
H	-0.733951	-3.663529	-0.792324
H	0.411811	-0.511060	1.904914
H	-0.155245	-2.923725	1.525268
H	-0.756610	-1.975981	-2.640069
S	0.324223	1.691830	0.280338
O	2.675805	-1.605382	-0.881479

O	-0.737030	2.331115	-0.444723
O	0.486525	1.782838	1.698818
C	1.940100	2.160562	-0.625871
F	1.630586	3.212620	-1.351077
F	2.308601	1.174335	-1.403609
F	2.850491	2.470203	0.250999
S	3.331966	-1.250518	0.376957
O	2.747827	-0.076003	1.045560
O	3.636886	-2.368272	1.266381
C	4.993922	-0.639921	-0.140177
F	5.639639	-1.576603	-0.830294
F	5.724617	-0.324574	0.925878
F	4.882815	0.442888	-0.905454
P	-3.250135	-0.392686	0.309371
O	-4.118192	0.191857	1.580248
O	-4.262542	0.080571	-0.895609
O	-3.641666	-1.962803	0.313520
C	-3.398703	0.850012	2.636951
H	-3.984778	0.696654	3.542356
H	-2.424354	0.370834	2.780994
C	-3.232557	2.325127	2.330206
H	-4.208283	2.794680	2.192904
H	-2.714227	2.827116	3.149554
H	-2.648426	2.464741	1.416423
C	-5.015153	-2.416410	0.326984
H	-5.664705	-1.593949	0.631901
H	-5.066320	-3.198910	1.084382
C	-5.397519	-2.945142	-1.039543
H	-6.412946	-3.346113	-1.013036
H	-5.352100	-2.143695	-1.777192
H	-4.718333	-3.744811	-1.340211
C	-4.308315	1.487632	-1.174228
H	-3.286953	1.885105	-1.217241
H	-4.836401	1.991590	-0.359405
C	-5.022186	1.684868	-2.492625
H	-5.088358	2.748226	-2.728269
H	-4.482967	1.181033	-3.296030
H	-6.032733	1.276894	-2.438898
H	-0.232388	0.398259	-2.096316

o-4

Symbol	X	Y	Z
C	0. -0.836021	-0.583651	0.075532
C	0. -0.541297	-1.834050	-0.711195
C	-0.556820	-1.800828	-2.044049
C	-0.766577	-0.546178	-2.760875
C	-0.697748	0.614720	-2.105825
N	-0.534890	0.625799	-0.697580
H	-0.344078	-2.696130	-2.614171
H	-0.261771	-0.575721	1.002764
H	-0.314197	-2.727293	-0.144391
H	-0.921983	-0.540670	-3.830181
S	0.072691	1.976564	0.042882
O	2.887341	-1.605767	-1.418595
O	-0.581552	3.119069	-0.550374
O	0.046398	1.736069	1.464435
C	1.885354	2.149346	-0.493330
F	2.021891	3.356301	-1.016836
F	2.188905	1.251085	-1.403523
F	2.661446	2.047672	0.559353
S	2.810766	-1.568766	0.039439
O	1.886063	-0.574347	0.600283
O	2.761520	-2.873183	0.699848
C	4.457097	-0.895907	0.526131
F	5.430082	-1.740346	0.185441
F	4.518616	-0.699451	1.841618
F	4.686657	0.267158	-0.080774
P	-2.610054	-0.496266	0.497136
O	-2.926312	0.765366	1.364149
O	-3.487112	-0.352638	-0.781369
O	-2.941986	-1.840051	1.197978
C	-2.564726	0.894976	2.786061
H	-3.232837	0.237413	3.340791
H	-1.530896	0.572336	2.906325
C	-2.741486	2.344777	3.150842
H	-3.771735	2.659040	2.982299
H	-2.504258	2.475465	4.207629
H	-2.067991	2.965320	2.559992
C	-4.287922	-2.436373	1.281632
H	-5.024734	-1.633156	1.253396
H	-4.306227	-2.908396	2.259916
C	-4.459601	-3.421462	0.152710
H	-5.429156	-3.911615	0.254429
H	-4.423823	-2.915262	-0.812588
H	-3.678718	-4.181317	0.191436
C	-3.976272	0.921589	-1.332074
H	-3.142444	1.625096	-1.354187
H	-4.743137	1.287707	-0.651728

C -4.508718 0.625583 -2.708764
H -4.902165 1.546463 -3.141572
H -3.713779 0.245429 -3.350895
H -5.313622 -0.107977 -2.657057
H -0.767544 1.588712 -2.572463

o-5

Symbol	X	Y	Z
C	0. 0.848506	-0.196817	-0.484176
C	0. 0.462115	0.217667	-1.878535
C	0.371828	-0.693699	-2.847068
C	0.570885	-2.113282	-2.563163
C	0.608374	-2.543462	-1.300918
N	0.552562	-1.613056	-0.232648
H	0.089798	-0.398485	-3.849768
H	0.342663	0.434477	0.249790
H	0.262759	1.271653	-2.026157
H	0.645694	-2.831238	-3.367317
S	0.087305	-2.120215	1.271942
O	-2.934075	-0.843074	-2.192236
O	0.815923	-3.335105	1.557221
O	0.140228	-0.974660	2.146816
C	-1.720034	-2.689803	1.173239
F	-1.775985	-3.888781	1.728705
F	-2.113836	-2.779221	-0.078769
F	-2.479186	-1.866218	1.855797
S	-2.864924	0.175752	-1.153531
O	-1.910409	-0.055076	-0.068319
O	-2.871828	1.561417	-1.654594
C	-4.495855	0.042244	-0.302563
F	-5.489808	0.293028	-1.150879
F	-4.571897	0.915642	0.700556
F	-4.662903	-1.180796	0.191765
P	2.654096	-0.030524	-0.237141
O	3.056623	-0.300548	1.249259
O	3.419076	-1.113460	-1.059993
O	3.066897	1.368007	-0.763360
C	2.748172	0.603453	2.370644
H	3.405353	1.467158	2.267581
H	1.709325	0.918749	2.280314
C	2.999888	-0.173106	3.635574
H	4.036784	-0.507081	3.682766
H	2.800788	0.471677	4.492895
H	2.335382	-1.035855	3.682988
C	4.461061	1.772309	-1.005428
H	5.116482	1.154812	-0.389379
H	4.503471	2.800380	-0.655345
C	4.763424	1.641562	-2.477512
H	5.775583	2.002844	-2.666138
H	4.698428	0.600038	-2.793951
H	4.064570	2.240350	-3.061946
C	3.857594	-2.417966	-0.545676
H	3.025056	-2.872088	-0.005513
H	4.676953	-2.228790	0.146032
C	4.280337	-3.237166	-1.736979
H	4.633312	-4.210021	-1.391888
H	3.439131	-3.386771	-2.414321
H	5.090448	-2.741627	-2.272738
H	0.694833	-3.582234	-1.009249
I	0.303746	3.398000	0.806314
Na	-2.512503	3.456746	-0.500890

o-6

Symbol	X	Y	Z
C	0. 0.175810	-0.217790	-0.618394
C	0. 0.307395	-0.064571	-2.110608
C	0.681668	-1.108554	-2.852149
C	0.892728	-2.422418	-2.247717
C	0.460424	-2.669011	-1.010287
N	-0.092757	-1.610642	-0.230907
H	0.785138	-1.008454	-3.925086
H	-0.610369	0.449519	-0.244634
H	0.085022	0.913547	-2.517384
H	1.352103	-3.219807	-2.814550
S	-1.328820	-1.936153	0.817592
O	-1.146672	-3.267795	1.330002
O	-1.504929	-0.779384	1.657712
C	-2.810443	-1.994006	-0.305954
F	-3.904541	-2.131654	0.413655
F	-2.678429	-3.014284	-1.132673
F	-2.859445	-0.865813	-0.993167
P	1.711979	0.267475	0.240543
O	1.526523	0.254600	1.789429
O	2.856640	-0.748335	-0.050239
O	2.102806	1.648755	-0.343776
C	0.688453	1.221219	2.524827
H	1.243888	2.158517	2.547602
H	-0.242693	1.358359	1.974281

C	0.460023	0.647837	3.897478
H	1.408822	0.486309	4.409828
H	-0.134875	1.353725	4.479138
H	-0.083869	-0.293592	3.825196
C	3.430976	2.266737	-0.180890
H	3.887743	1.871946	0.727738
H	3.215104	3.322936	-0.045220
C	4.256565	1.991269	-1.412778
H	5.213264	2.507755	-1.320662
H	4.444661	0.922898	-1.522956
H	3.744472	2.361664	-2.301084
C	3.213563	-1.894724	0.798748
H	2.296619	-2.426697	1.055503
H	3.665701	-1.491962	1.703372
C	4.163315	-2.748184	0.000836
H	4.466526	-3.602874	0.607122
H	3.678504	-3.112588	-0.905277
H	5.052656	-2.178559	-0.269876
H	0.500934	-3.629425	-0.514950
I	-1.678390	3.031959	-0.350480

o-7

Symbol	X	Y	Z
C	0. -1.067654	-0.109861	-0.762755
C	0. -0.284528	-1.194386	-1.463259
C	0.063104	-2.292128	-0.788202
C	-0.372924	-2.499469	0.589632
C	-1.341246	-1.739671	1.103550
N	-1.858507	-0.649588	0.349662
H	0.633090	-3.072719	-1.276161
H	-1.725490	0.402320	-1.469216
H	-0.037434	-1.041719	-2.505825
H	0.046172	-3.298080	1.184378
S	-3.435997	-0.199896	0.529953
O	-3.845666	-0.491887	1.876572
O	-3.596264	1.092153	-0.088450
C	-4.323274	-1.405334	-0.573844
F	-4.178898	-2.621598	-0.084872
F	-3.780574	-1.341742	-1.779344
F	-5.599597	-1.089244	-0.640201
P	0.031684	1.193250	-0.104795
O	-0.793246	2.316438	0.589123
O	0.919896	0.505195	0.956660
C	-1.480053	3.403848	-0.128329
H	-0.705540	4.039832	-0.554064
H	-2.081438	2.960241	-0.922011
C	-2.335227	4.118418	0.883594
H	-1.721028	4.512295	1.693644
H	-2.843316	4.950396	0.393777
H	-3.083541	3.437900	1.289351
C	1.838709	1.201790	1.880426
H	1.379135	2.151909	2.151619
H	2.765596	1.356629	1.328108
C	2.034717	0.291847	3.062775
H	2.730103	0.763856	3.759162
H	1.089088	0.112380	3.574765
H	2.463586	-0.655419	2.734527
O	0.830985	1.826659	-1.281326
C	2.136072	1.321948	-1.758835
H	2.895018	1.755899	-1.110254
H	2.159873	0.240153	-1.628520
C	2.275774	1.742842	-3.195812
H	1.477294	1.314940	-3.802682
H	2.251367	2.829104	-3.282093
H	3.235172	1.383809	-3.572690
I	4.705805	-0.867640	0.067956
H	-1.779974	-1.866353	2.083021

o-8

Symbol	X	Y	Z
C	0. -1.048927	0.083710	-0.867774
C	0. 0.026070	-0.785704	-1.468278
C	0.716959	-1.628810	-0.697909
C	0.401514	-1.775054	0.721597
C	-0.739965	-1.282601	1.204546
N	-1.599056	-0.531153	0.354627
H	1.502948	-2.242260	-1.122311
H	-1.858771	0.222091	-1.587683
H	0.209879	-0.681771	-2.529887
H	1.071068	-2.310451	1.380114
S	-3.222638	-0.638138	0.559306
O	-3.507269	-0.819403	1.959333
O	-3.853605	0.376670	-0.246894
C	-3.637289	-2.251085	-0.263766
F	-3.054949	-3.238511	0.392861
F	-3.184558	-2.219144	-1.507597
F	-4.944701	-2.426370	-0.270747
P	-0.436848	1.775978	-0.494564

O	-1.687521	2.565835	0.101797
O	0.436967	1.518330	0.805060
C	-2.647234	3.192114	-0.787129
H	-2.134849	3.977920	-1.343911
H	-3.023915	2.441229	-1.485661
C	-3.764123	3.737069	0.068941
H	-3.375234	4.462470	0.784818
H	-4.502628	4.232452	-0.563318
H	-4.250500	2.923765	0.608063
C	1.198982	2.589382	1.417080
H	0.497002	3.329931	1.804767
H	1.822015	3.054209	0.649256
C	2.030174	1.974658	2.517752
H	2.619313	2.749622	3.010224
H	1.386040	1.499831	3.258804
H	2.710976	1.223554	2.111598
O	0.237840	2.419101	-1.644404
C	3.293541	0.701173	-0.715622
H	3.789196	1.605571	-0.370064
H	2.318351	0.606260	-0.247718
C	3.226024	0.607906	-2.224451
H	2.821752	-0.353021	-2.545066
H	2.554062	1.394811	-2.579899
H	4.208131	0.743400	-2.678358
I	4.440348	-0.918943	0.159436
H	-1.082780	-1.391620	2.223624

o-9

Symbol	X	Y	Z
C	0. -0.197230	-0.449067	1.078481
C	0. -0.225333	-1.792802	1.760622
C	-0.115105	-2.914529	1.046955
C	0.103089	-2.866041	-0.397055
C	0.449612	-1.719185	-0.981474
N	0.522753	-0.528702	-0.206927
H	-0.152043	-3.879630	1.536465
H	0.301923	0.283593	1.716678
H	-0.351371	-1.797229	2.835459
H	0.013941	-3.760445	-0.997110
S	1.631802	0.611984	-0.599544
O	1.785833	0.648676	-2.031283
O	1.381218	1.789475	0.193471
C	3.210226	-0.104041	0.068331
F	3.494040	-1.218476	-0.582649
F	3.040601	-0.372852	1.353341
F	4.193248	0.763557	-0.078598
P	-1.883891	0.228532	0.805114
O	-1.675642	1.539977	-0.081767
O	-2.472097	-0.794926	-0.252731
C	-1.498034	2.831821	0.552620
H	-2.417347	3.079391	1.084879
H	-0.675395	2.764283	1.267507
C	-1.187217	3.827927	-0.537572
H	-2.002004	3.866130	-1.261957
H	-1.060476	4.820611	-0.102620
H	-0.265391	3.547404	-1.048042
C	-3.709002	-0.518136	-0.956949
H	-3.579118	0.406058	-1.522533
H	-4.504397	-0.380381	-0.221620
C	-3.984579	-1.695131	-1.861356
H	-4.908363	-1.523966	-2.415935
H	-3.168199	-1.825522	-2.572860
H	-4.092606	-2.608527	-1.275262
O	-2.638896	0.433622	2.060816
H	0.678231	-1.600134	-2.031092

o-$TS_{10/11}$

Symbol	X	Y	Z
C	0. -0.442803	-0.244157	-0.728812
C	0. -0.379623	-0.401058	-2.212403
C	-0.855230	0.514513	-3.068526
C	-1.578279	1.675350	-2.558629
C	-1.552608	1.968552	-1.259209
N	-0.760897	1.163838	-0.379027
H	-0.762946	0.373442	-4.137827
H	0.932990	-0.755037	-0.347779
H	0.059974	-1.318759	-2.590623
H	-2.192182	2.271475	-3.220667
S	-0.090917	1.908148	0.898766
O	-0.996684	2.918226	1.398904
O	0.521219	0.940327	1.775706
C	1.325357	2.886004	0.200325
F	0.867670	3.812932	-0.625003
F	2.135522	2.072190	-0.470105
F	2.005404	3.460918	1.177986
P	-1.708116	-1.314640	-0.044092
O	-1.839440	-0.957698	1.520257
O	-3.102568	-0.726775	-0.576359

C	-0.985824	-1.624490	2.468935
H	-1.228830	-2.689082	2.468295
H	0.053978	-1.499720	2.157624
C	-1.218029	-0.992861	3.820931
H	-2.263423	-1.099151	4.115102
H	-0.594070	-1.481545	4.571236
H	-0.961932	0.066297	3.783700
C	-4.343473	-1.308419	-0.122758
H	-4.418845	-1.166211	0.957597
H	-4.330108	-2.379568	-0.338089
C	-5.468852	-0.613040	-0.853153
H	-6.428410	-1.023276	-0.534266
H	-5.457544	0.456289	-0.637495
H	-5.368525	-0.755811	-1.929958
O	-1.504096	-2.764133	-0.308846
H	-2.105600	2.773637	-0.796175
N	2.077850	-1.307591	-0.322741
C	1.729829	-2.757000	-0.313325
H	1.458974	-3.005132	0.715942
H	0.814172	-2.861754	-0.899184
C	2.698681	-0.839648	-1.596289
H	2.282467	-1.453235	-2.395970
H	2.335130	0.176228	-1.760748
C	2.764936	-0.844306	0.915172
H	3.087950	0.181246	0.731668
H	1.996021	-0.789146	1.684178
C	3.919361	-1.691942	1.437787
H	4.312226	-1.200817	2.330087
H	3.582182	-2.685763	1.735740
H	4.736554	-1.797225	0.726346
C	2.783290	-3.723743	-0.841759
H	2.382589	-4.735710	-0.761095
H	3.000684	-3.543163	-1.895615
H	3.716736	-3.686304	-0.282476
C	4.219928	-0.847576	-1.675969
H	4.505212	-0.472968	-2.660481
H	4.667682	-0.188213	-0.931179
H	4.639864	-1.846542	-1.561504

o-TS$_{3/4}$

Symbol	X	Y	Z
C	0. 0.496811	-0.725206	-0.226363
C	0. 0.593124	-2.071443	0.257723
C	0.597649	-2.307668	1.593177
C	0.454649	-1.231187	2.522078
C	0.189107	0.007542	2.059506
N	0.083988	0.222112	0.691660
H	0.688737	-3.320361	1.964219
H	0.191786	-0.530614	-1.243131
H	0.706941	-2.859486	-0.473415
H	0.510311	-1.397212	3.587820
S	-0.163479	1.820831	0.125729
O	-2.557142	-1.533935	1.040002
O	0.608024	2.671699	0.996022
O	0.042192	1.790770	-1.295988
C	-1.985369	2.209600	0.517911
F	-1.972941	3.353751	1.172411
F	-2.487503	1.270299	1.280326
F	-2.650436	2.345288	-0.597174
S	-2.814118	-1.405567	-0.392378
O	-2.143711	-0.266380	-1.038897
O	-2.760348	-2.651375	-1.155971
C	-4.590435	-0.918015	-0.478685
F	-5.356703	-1.830466	0.115742
F	-4.983546	-0.803504	-1.745383
F	-4.787578	0.251537	0.126115
P	2.791711	-0.320161	-0.585293
O	3.568558	0.943756	-1.208047
O	3.557375	-0.459411	0.818454
O	3.256323	-1.630283	-1.353375
C	3.112118	1.547227	-2.448838
H	3.803107	1.226203	-3.228177
H	2.111341	1.178437	-2.688022
C	3.107245	3.046574	-2.272006
H	4.102642	3.400130	-1.999955
H	2.809640	3.525850	-3.206060
H	2.401041	3.333169	-1.491447
C	4.642240	-2.086533	-1.371203
H	5.296328	-1.241248	-1.149334
H	4.820364	-2.412948	-2.393846
C	4.813450	-3.211209	-0.376015
H	5.832912	-3.597241	-0.432961
H	4.627788	-2.853743	0.637205
H	4.121652	-4.023887	-0.601850
C	3.684659	0.677195	1.710707
H	2.735672	1.222146	1.745799
H	4.448141	1.339536	1.301636
C	4.061129	0.145778	3.072026

H	4.194296	0.976782	3.766263
H	3.278496	-0.511003	3.454286
H	4.995735	-0.413714	3.013947
H	0.020973	0.865833	2.695142

o-TS$_{7/8}$

Symbol	X	Y	Z
C	0. -1.190893	-0.279792	-0.578386
C	0. -0.395867	-1.527259	-0.877070
C	-0.222954	-2.449841	0.071835
C	-0.865429	-2.314437	1.376764
C	-1.830849	-1.412110	1.555218
N	-2.158295	-0.509605	0.506161
H	0.359027	-3.339730	-0.133023
H	-1.723998	0.049382	-1.473623
H	0.014530	-1.626900	-1.873503
H	-0.596015	-2.969695	2.192551
S	-3.707361	0.018587	0.343117
O	-4.315037	0.088276	1.645531
O	-3.709303	1.116309	-0.591052
C	-4.525945	-1.396834	-0.539437
F	-4.500817	-2.462572	0.239523
F	-3.852497	-1.643967	-1.652007
F	-5.771204	-1.079989	-0.831734
P	-0.078586	1.105299	-0.136605
O	-0.952397	2.389162	0.137894
O	0.485593	0.689042	1.269990
C	-1.443697	3.224443	-0.954464
H	-0.580308	3.698843	-1.420783
H	-1.948612	2.584669	-1.680466
C	-2.397530	4.224640	-0.352920
H	-1.890631	4.829406	0.399780
H	-2.771495	4.884345	-1.137262
H	-3.240902	3.707294	0.104606
C	1.376641	1.559584	2.033239
H	0.747619	2.249829	2.594292
H	1.999389	2.126345	1.336538
C	2.207707	0.675968	2.929703
H	2.854943	1.297617	3.550190
H	1.563107	0.083217	3.579644
H	2.834685	0.007293	2.336764
O	0.977265	1.309244	-1.195203
C	2.719192	0.390541	-1.066260
H	3.176924	1.351020	-0.897242
H	2.395248	-0.169449	-0.202468
C	2.758013	-0.241300	-2.418998
H	2.676228	-1.324432	-2.350016
H	1.918815	0.145269	-3.000693
H	3.677721	0.021726	-2.937260
I	5.219674	-0.573770	-0.102826
H	-2.402990	-1.285011	2.463167

POEt$_3$

Symbol	X	Y	Z
P	0. 0.000785	-0.000569	1.370982
O	0. 1.359404	-0.574695	0.652651
O	-1.176517	-0.889004	0.652768
O	-0.182084	1.463300	0.653552
C	1.916617	-0.113904	-0.589381
H	1.283861	-0.441568	-1.419798
H	1.943080	0.978575	-0.582663
C	3.308019	-0.693139	-0.716062
H	3.267712	-1.783436	-0.718743
H	3.768762	-0.359085	-1.647369
H	3.929550	-0.367461	0.119109
C	-0.859252	1.715509	-0.588878
H	-1.818370	1.191717	-0.582989
H	-0.258165	1.331753	-1.418806
C	-1.053976	3.210114	-0.715450
H	-1.573807	3.442187	-1.646648
H	-1.646868	3.585154	0.119831
H	-0.089788	3.720744	-0.718046
C	-1.058892	-1.599601	-0.590933
H	-0.124626	-2.166389	-0.589289
H	-1.031553	-0.885757	-1.419862
C	-2.254526	-2.517691	-0.714508
H	-2.197523	-3.082407	-1.646753
H	-2.278283	-3.219947	0.119812
H	-3.180125	-1.940076	-0.712996

PPh$_3$

Symbol	X	Y	Z
P	0. -0.003388	0.000219	-1.281552
C	0. 0.634871	-1.516672	-0.450870
C	0.046743	-2.106287	0.670492
C	1.792882	-2.088639	-0.988936
C	0.612550	-3.241700	1.245474
H	-0.853087	-1.680002	1.099708

C	2.364047	-3.214409	-0.406781
H	2.252196	-1.647120	-1.867897
C	1.772198	-3.794860	0.712036
H	0.146664	-3.691540	2.114359
H	3.264238	-3.643237	-0.830746
H	2.210894	-4.676893	1.163013
C	0.994017	1.308213	-0.449070
C	0.936951	2.593515	-0.998866
C	1.778976	1.091655	0.685855
C	1.634487	3.645395	-0.415722
H	0.340520	2.771881	-1.888174
C	2.486939	2.143175	1.262151
H	1.838758	0.101801	1.124124
C	2.413694	3.420642	0.716076
H	1.577581	4.636695	-0.849405
H	3.094366	1.962764	2.141261
H	2.964713	4.237118	1.167285
C	-1.635386	0.206832	-0.449727
C	-2.712010	-0.508525	-0.985473
C	-1.849808	1.016284	0.668112
C	-3.972604	-0.432985	-0.404377
H	-2.561222	-1.130879	-1.862106
C	-3.115982	1.101470	1.241742
H	-1.029340	1.581825	1.095183
C	-4.177197	0.375783	0.710530
H	-4.795947	-0.996707	-0.826665
H	-3.271040	1.734609	2.107442
H	-5.160694	0.443082	1.160016

pyridine

Symbol	X	Y	Z
C	0. -1.141255	-0.717061	0.001090
C	0. -1.192985	0.673281	-0.000409
C	0.003100	1.379879	-0.000205
C	1.196191	0.667291	0.000887
C	1.137900	-0.721645	-0.002307
N	-0.003350	-1.414926	0.000192
H	0.006476	2.463306	-0.000216
H	-2.059249	-1.296665	0.000650
H	-2.148329	1.182026	-0.001664
H	2.153672	1.172223	0.002825
H	2.053169	-1.306887	0.002732

Tf_2O

Symbol	X	Y	Z
S	0. 1.181364	0.914546	0.008669
O	0. 0.000016	0.000172	0.724322
O	0.814157	1.204048	-1.340191
O	1.513134	1.901766	0.981783
C	2.499709	-0.406140	0.001416
F	1.986859	-1.499898	-0.519334
F	2.889964	-0.620637	1.234157
F	3.495678	0.028790	-0.734823
S	-1.181365	-0.914549	0.008743
O	-0.814139	-1.204116	-1.340081
O	-1.512886	-1.901660	0.982032
C	-2.499776	0.406107	0.001477
F	-2.889827	0.620823	1.234216
F	-3.495831	-0.029008	-0.734540
F	-1.987047	1.499771	-0.519549

TfONa

Symbol	X	Y	Z
O	0. -1.010584	0.244740	-1.216350
S	0. -0.293225	0.656827	-0.000003
O	0.238813	2.010569	0.000105
O	-1.010685	0.244593	1.216243
C	1.201047	-0.422673	-0.000001
F	0.843204	-1.703505	0.000189
F	1.940126	-0.189673	1.079295
F	1.939934	-0.189940	-1.079482
Na	-2.796766	-0.838576	0.000004

$TS_{10/11}$

Symbol	X	Y	Z
C	0. -1.305893	-1.153525	1.136585
C	0. -0.105105	-0.611384	1.328820
C	0.753145	-0.090244	0.239307
C	0.079068	-0.145429	-1.080812
C	-1.122086	-0.682977	-1.279470
N	-1.890239	-1.223328	-0.183446
H	1.841948	-1.058162	0.110883
H	-1.896152	-1.618404	1.911951
H	0.284429	-0.613327	2.341668
H	0.611523	0.209198	-1.957661
S	-3.519741	-1.190012	-0.302454
O	-3.890982	-1.449223	-1.671862
O	-4.081472	-1.925343	0.803712

C	-3.999231	0.583217	-0.001522
F	-5.310201	0.719066	-0.089938
F	-3.412505	1.354045	-0.906106
F	-3.597567	0.950055	1.206940
P	1.507749	1.455621	0.613570
O	2.725818	1.552465	-0.425138
O	0.589992	2.664671	0.040466
C	3.456028	2.791271	-0.555738
H	2.810969	3.519751	-1.050365
H	3.705453	3.161643	0.441925
C	4.702167	2.510994	-1.362383
H	5.328197	1.775853	-0.853634
H	4.439421	2.126957	-2.349214
H	5.275374	3.431072	-1.487345
C	-0.717011	2.844109	0.616981
H	-0.605997	3.206662	1.642044
H	-1.229909	1.877456	0.644959
C	-1.477533	3.827585	-0.241810
H	-0.960428	4.787676	-0.271481
H	-1.572040	3.443378	-1.258655
H	-2.478049	3.980870	0.166483
H	-1.579649	-0.807390	-2.249693
O	1.881035	1.644840	2.042906
N	2.677320	-2.016603	0.013760
C	2.148319	-3.013996	-0.952813
H	1.944386	-2.476365	-1.878583
H	2.938675	-3.746980	-1.154654
C	3.976333	-1.452946	-0.438606
H	4.185710	-0.594348	0.199779
H	4.754023	-2.207373	-0.269577
C	2.774983	-2.577800	1.385565
H	1.761640	-2.843402	1.688330
H	3.367114	-3.499591	1.342084
C	3.357746	-1.601941	2.398765
H	3.217118	-2.016429	3.397943
H	4.426471	-1.444287	2.252631
H	2.852382	-0.632997	2.359529
C	0.876549	-3.714327	-0.497140
H	0.473496	-4.276161	-1.340765
H	1.056575	-4.417928	0.315686
H	0.119309	-2.994938	-0.179400
C	3.974404	-0.996105	-1.889477
H	4.900498	-0.451931	-2.079251
H	3.928815	-1.829754	-2.590046
H	3.144793	-0.312155	-2.078286

$TS_{12/13}$

Symbol	X	Y	Z
C	0. 1.245869	-2.149415	-0.068301
C	0. 0.355348	-1.189809	-0.443002
C	0.124385	-0.067465	0.398603
C	0.640245	-0.142297	1.721319
C	1.523299	-1.124267	2.045666
N	2.107189	-1.913295	1.027369
H	-2.734413	-0.726950	-0.691371
H	1.417249	-3.060011	-0.625430
H	-0.224992	-1.317785	-1.352329
H	0.260044	0.520470	2.491183
S	3.740564	-1.176936	0.457769
O	4.506682	-0.840156	1.657055
O	4.300630	-2.100845	-0.528274
C	3.690876	0.454230	-0.490135
F	4.928621	0.881437	-0.715228
F	3.043227	1.381817	0.204217
F	3.082488	0.285055	-1.659222
P	-1.009869	1.156404	-0.092043
O	-1.763504	1.602020	1.244071
O	-0.287559	2.534335	-0.491781
C	-2.530572	2.829617	1.286111
H	-1.834281	3.656425	1.430612
H	-3.041774	2.968693	0.329548
C	-3.515608	2.714657	2.425072
H	-4.207496	1.888113	2.253408
H	-2.989781	2.543926	3.365340
H	-4.091228	3.637610	2.509935
C	0.497137	2.563938	-1.704405
H	-0.186751	2.623587	-2.553028
H	1.070647	1.635756	-1.778334
C	1.416375	3.760676	-1.637729
H	0.839016	4.680560	-1.535112
H	2.093891	3.669345	-0.788251
H	2.008463	3.821137	-2.552421
H	1.886937	-1.297092	3.048781
O	-1.931537	0.685694	-1.185263
N	-3.439929	-1.468029	-0.460610
C	-3.534175	-1.592383	1.036343
H	-3.569369	-0.572034	1.417854
H	-4.490215	-2.076127	1.247618

C -4.721488 -0.943656 -1.042852
H -4.521331 -0.749285 -2.094494
H -5.458151 -1.746226 -0.966497
C -2.997358 -2.733903 -1.128082
H -2.040367 -2.998102 -0.680838
H -3.725572 -3.506608 -0.872343
C -2.835253 -2.568080 -2.630578
H -2.284842 -3.426343 -3.015566
H -3.790503 -2.521541 -3.151626
H -2.262605 -1.665420 -2.860065
C -2.385640 -2.346763 1.685221
H -2.482770 -2.224659 2.764754
H -2.417635 -3.414089 1.467685
H -1.413653 -1.947068 1.389881
C -5.192340 0.333973 -0.367893
H -6.002133 0.757152 -0.962187
H -5.571310 0.159630 0.638620
H -4.380206 1.063623 -0.332832

$TS_{1/2}$

Symbol X Y Z
C 0. -3.161674 -0.941275 -1.051178
C 0. -4.468998 -1.366001 -1.247664
C -5.404012 -1.116346 -0.250254
C -4.998394 -0.455389 0.903150
C -3.669763 -0.067160 1.011081
N -2.772415 -0.301395 0.052430
H -6.432559 -1.434603 -0.368605
H -2.392025 -1.119814 -1.794683
H -4.740902 -1.880837 -2.159457
H -5.692041 -0.244764 1.706167
S -0.275208 -0.068230 0.562245
O 1.371978 0.369048 0.854883
O -0.187045 -1.129176 -0.391530
O -0.722367 -0.148344 1.915497
C -0.758118 1.589059 -0.246200
F 0.292345 2.384857 -0.285555
F -1.691440 2.146986 0.481283
F -1.164311 1.342270 -1.465527
S 2.540639 0.487241 -0.246338
O 1.972245 0.462184 -1.563839
O 3.429106 1.516459 0.198431
C 3.374870 -1.143519 0.064711
F 3.810943 -1.170823 1.305245
F 4.384502 -1.243686 -0.775426
F 2.515072 -2.117021 -0.138715
H -3.301271 0.444788 1.893890

$TS_{16/17}$

Symbol X Y Z
C 0. 0.415127 1.005227 -1.181400
C 0. -0.669713 0.240653 -1.203017
C -1.366109 -0.248656 0.035414
C -0.716841 0.282073 1.278976
C 0.377973 1.033861 1.269172
N 0.977593 1.460467 0.047504
H -1.179809 -1.623793 0.008488
H 0.975423 1.276220 -2.063228
H -1.031494 -0.087714 -2.169873
H -1.092994 -0.027013 2.247674
S 1.968132 2.762218 0.048829
O 4.719257 0.333285 0.110639
O 2.624664 2.850137 1.327203
O 2.665497 2.828928 -1.208329
C 0.785772 4.195398 0.011682
F 1.449872 5.334736 0.006668
F -0.001490 4.143572 1.077764
F 0.037427 4.110693 -1.080388
S 3.619266 -0.624270 -0.017661
O 2.769204 -0.754121 1.167236
O 2.909886 -0.585060 -1.297225
C 4.487640 -2.251976 -0.079487
F 5.307837 -2.314115 -1.127452
F 3.610244 -3.253663 -0.183320
F 5.207702 -2.449351 1.023944
N -0.742683 -2.930241 -0.068083
C 0.611044 -2.931904 -0.694210
H 1.179990 -2.127721 -0.225439
H 1.095676 -3.881346 -0.434476
C -0.647978 -3.475972 1.310511
H -1.599533 -3.281494 1.807620
H -0.538765 -4.565993 1.238352
C -1.710411 -3.706486 -0.881742
H -1.873997 -3.145674 -1.804211
H -1.247742 -4.664782 -1.149483
C -3.047950 -3.955662 -0.200929
H -3.727826 -4.401562 -0.928563
H -2.964880 -4.641648 0.641975

H	-3.500750	-3.024429	0.139963
C	0.645426	-2.762766	-2.204491
H	1.690283	-2.631804	-2.488050
H	0.247976	-3.631239	-2.730866
H	0.107901	-1.874547	-2.533271
C	0.492052	-2.895756	2.137258
H	0.360549	-3.209391	3.173989
H	1.466022	-3.245532	1.795636
H	0.513409	-1.806409	2.099489
P	-3.064535	0.154410	-0.066118
O	-3.409307	1.575552	-0.652141
O	-3.743939	-0.854836	-1.052888
O	-3.775559	0.145007	1.341080
C	-2.843549	2.783952	-0.056885
H	-3.311921	2.925135	0.917985
H	-1.772514	2.623755	0.076586
C	-3.119848	3.926494	-0.999477
H	-4.193225	4.056584	-1.138836
H	-2.706748	4.845092	-0.580076
H	-2.649546	3.741870	-1.965898
C	-3.889876	-1.042206	2.178702
H	-2.943753	-1.587891	2.147500
H	-4.678628	-1.666963	1.756989
C	-4.217955	-0.580347	3.575840
H	-4.333369	-1.449380	4.224588
H	-3.418525	0.048449	3.968884
H	-5.150556	-0.015845	3.577310
C	-5.082013	-0.635893	-1.608446
H	-4.978601	0.098865	-2.405719
H	-5.722454	-0.230662	-0.823051
C	-5.579536	-1.967610	-2.109501
H	-6.549347	-1.830093	-2.589673
H	-4.883756	-2.382970	-2.839755
H	-5.694442	-2.670991	-1.284502
H	0.911767	1.323467	2.161703

$TS_{18/19}$

Symbol	X	Y	Z
C	0. -1.204930	1.781622	-0.727152
C	0. -0.691401	0.528680	-0.614692
C	-0.966989	-0.234434	0.553962
C	-1.552472	0.448797	1.660471
C	-2.032666	1.708681	1.489721
N	-2.154016	2.259862	0.197747
H	2.158952	1.951096	0.197003
H	-1.003099	2.428300	-1.570687
H	-0.010635	0.138557	-1.363762
H	-1.552266	0.005291	2.651560
S	-3.936262	1.971963	-0.482132
O	2.280361	0.200496	-0.103683
O	-4.852884	2.628016	0.450782
O	-3.930315	2.365823	-1.891199
C	-4.585560	0.194312	-0.532588
F	-5.700132	0.134776	-1.247991
F	-4.844093	-0.231001	0.699691
F	-3.684934	-0.615752	-1.081767
S	3.058056	-0.254555	-1.274960
O	2.263065	-0.815559	-2.362211
O	4.105362	0.686253	-1.672881
C	4.004495	-1.686688	-0.593333
F	4.699300	-1.301911	0.476052
F	4.854262	-2.147452	-1.506058
F	3.198248	-2.677851	-0.231801
N	2.329126	2.954145	0.410284
C	1.613974	3.762357	-0.635802
H	0.558809	3.742774	-0.368526
H	1.976522	4.787899	-0.549003
C	1.791174	3.210768	1.796717
H	2.492369	3.871301	2.306952
H	0.847477	3.742392	1.683130
C	3.817885	3.151496	0.319768
H	4.097801	2.909596	-0.702754
H	3.996651	4.211541	0.507134
C	4.595120	2.269425	1.279288
H	5.657195	2.394396	1.066259
H	4.429664	2.540944	2.322418
H	4.341514	1.219136	1.131527
C	1.803843	3.195704	-2.031407
H	1.199143	3.781527	-2.724884
H	2.838066	3.232417	-2.370302
H	1.458407	2.159554	-2.076380
C	1.563594	1.906218	2.540614
H	1.105760	2.122559	3.506775
H	0.881507	1.271957	1.969760
H	2.491692	1.358895	2.708437
P	-0.516812	-1.890998	0.625115
O	-1.697433	-2.922797	0.434133
O	0.496890	-2.219194	-0.508664

O	0.081487	-2.283761	2.031307
C	-2.911680	-2.849929	1.232966
H	-2.697065	-3.312175	2.196966
H	-3.166965	-1.799423	1.384300
C	-4.001130	-3.573486	0.481256
H	-3.728465	-4.616792	0.319654
H	-4.923706	-3.539240	1.062804
H	-4.177007	-3.095901	-0.482996
C	1.357339	-1.726102	2.481176
H	1.659047	-0.926659	1.799882
H	2.079283	-2.538641	2.408059
C	1.191097	-1.240388	3.900131
H	2.150811	-0.879805	4.275062
H	0.471723	-0.421322	3.949542
H	0.849034	-2.051371	4.544176
C	0.289976	-3.101515	-1.651204
H	-0.148539	-4.030636	-1.288187
H	1.296746	-3.281522	-2.018426
C	-0.573082	-2.423309	-2.688742
H	-0.713120	-3.097491	-3.535760
H	-1.556975	-2.177672	-2.281272
H	-0.083702	-1.514500	-3.038803
H	-2.426563	2.306647	2.300161

$TS_{21/22}$

Symbol	X	Y	Z
C	0. -4.603007	-1.677353	-1.212211
C	0. -3.707866	-0.638337	-0.987923
C	-2.579138	-0.907386	-0.217039
C	-2.392837	-2.184701	0.298830
C	-3.353337	-3.149984	0.009941
N	-4.436398	-2.910138	-0.728232
H	-5.493811	-1.508514	-1.807362
H	-3.893421	0.345570	-1.401353
H	-1.530939	-2.422869	0.909700
P	-1.375668	0.374470	0.119850
O	-0.393642	-0.038921	1.184687
O	-0.728848	0.694400	-1.280317
O	-2.153862	1.706115	0.480174
C	1.523990	-0.397108	0.857754
H	1.341540	-0.477390	-0.201118
H	1.757092	0.577011	1.255307
C	1.658149	-1.610799	1.716063
H	0.802983	-1.641232	2.393767
H	1.676858	-2.519955	1.118499
H	2.561358	-1.551773	2.319834
C	-2.751431	1.883560	1.795811
H	-1.959913	1.799590	2.541199
H	-3.479635	1.083586	1.950908
C	-3.406232	3.242354	1.811984
H	-3.864016	3.413145	2.787202
H	-2.666232	4.022696	1.631466
H	-4.180901	3.301316	1.046912
C	0.140987	1.855273	-1.466797
H	0.989844	1.484080	-2.038302
H	0.497057	2.198646	-0.492453
C	-0.614866	2.937608	-2.200231
H	-0.984769	2.558729	-3.153505
H	-1.456175	3.289056	-1.602354
H	0.054520	3.777132	-2.395174
I	4.178971	-0.260901	-0.140940
H	-3.242745	-4.158660	0.393054

$TS_{3/4}$

Symbol	X	Y	Z
C	0. 0.291085	-0.195536	1.371792
C	0. -0.211135	-1.448013	1.404983
C	-0.647670	-2.081826	0.192930
C	-0.205094	-1.482208	-1.033301
C	0.306056	-0.231637	-1.029689
N	0.466734	0.443644	0.162488
H	-0.826741	-3.147032	0.207610
H	0.627380	0.346254	2.244296
H	-0.306276	-1.952402	2.356106
H	-0.308881	-2.004455	-1.973081
S	1.062696	2.048126	0.139575
O	3.210568	-0.732850	-1.527488
O	1.451447	2.400485	1.472656
O	1.858529	2.230268	-1.036721
C	-0.559041	2.907424	-0.182831
F	-0.358511	4.205945	-0.153131
F	-1.426528	2.555966	0.745156
F	-1.004661	2.542158	-1.372603
S	3.513663	-0.983774	-0.118017
O	3.220337	0.139627	0.780547
O	3.103129	-2.295076	0.383401
C	5.355973	-1.065620	-0.084581
F	5.795707	-2.045506	-0.872241

F 5.794311 -1.295711 1.151600
F 5.885199 0.079802 -0.508779
P -2.939362 -1.467826 0.111290
O -3.009050 -0.135022 1.024250
O -3.102094 -0.835887 -1.343845
O -4.284867 -2.271478 0.445013
C -3.251565 -0.263317 2.447727
H -4.311909 -0.481776 2.592031
H -2.671555 -1.102366 2.842755
C -2.858783 1.036006 3.106792
H -3.421829 1.864736 2.675717
H -3.074940 0.982937 4.174666
H -1.793562 1.229073 2.975488
C -5.602006 -1.671184 0.268400
H -5.514088 -0.585218 0.363575
H -6.198545 -2.042848 1.099884
C -6.183615 -2.080066 -1.066792
H -7.188079 -1.665649 -1.169299
H -5.568282 -1.715306 -1.890888
H -6.247339 -3.166697 -1.131487
C -3.569576 0.497961 -1.685619
H -3.176969 1.201090 -0.952319
H -4.660578 0.500142 -1.631212
C -3.080609 0.798917 -3.081118
H -3.386435 1.807157 -3.365654
H -1.991883 0.740682 -3.121937
H -3.499226 0.090481 -3.796801
H 0.663532 0.273627 -1.915829

TS_{5a}

Symbol X Y Z
C 0. -2.039470 -2.912332 0.122432
C 0. -0.802669 -2.784214 0.585629
C 0.121401 -1.682996 0.118511
C -0.325894 -1.155881 -1.218746
C -1.566426 -1.312704 -1.662069
N -2.508470 -2.082915 -0.928437
H 1.157726 -2.032355 0.058269
H -2.730311 -3.677831 0.445349
H -0.455322 -3.491085 1.326997
H 0.398733 -0.659334 -1.847068
S -4.115062 -1.948089 -1.234739
O 3.776156 -1.510645 0.567342
O -4.296006 -1.494074 -2.588331
O -4.789609 -3.098437 -0.693204
C -4.600777 -0.519004 -0.155936
F -5.895834 -0.297395 -0.273413
F -3.926426 0.553790 -0.531600
F -4.309845 -0.808803 1.101740
S 3.793047 -0.535247 -0.516436
O 2.660074 -0.611979 -1.453669
O 4.094421 0.849779 -0.125846
C 5.241443 -1.026756 -1.548904
F 6.356718 -0.971999 -0.829878
F 5.362156 -0.206872 -2.587063
F 5.084626 -2.265892 -2.000273
P 0.112606 -0.394492 1.416653
O 1.420443 0.499780 1.128479
O -1.156223 0.368818 1.544385
O 0.417890 -1.156732 2.785713
C 2.120714 1.143480 2.241707
H 1.515902 1.044477 3.144333
H 3.051138 0.588338 2.369817
C 2.377893 2.596818 1.924851
H 1.448473 3.108388 1.667663
H 2.807724 3.078993 2.804826
H 3.094464 2.695960 1.109545
C 1.594355 -1.986318 2.981756
H 2.384896 -1.682638 2.291894
H 1.309774 -3.015521 2.753717
C 2.025129 -1.850195 4.423976
H 2.875715 -2.506791 4.613540
H 2.322997 -0.824124 4.644929
H 1.211227 -2.133220 5.092527
C -1.874775 2.642655 2.196336
H -1.412940 3.527350 1.776334
H -1.336631 2.099133 2.960222
C -3.254323 2.351129 1.952110
H -3.694943 2.952608 2.784783
H -3.641301 2.755361 1.021236
H -3.517893 1.314675 2.142046
Na 1.967190 1.679026 -1.043185
I -0.766920 3.089563 -0.920875
H -1.918651 -0.947004 -2.615971

TS_{6a}

Symbol X Y Z
C 0. -1.796979 -1.510831 1.265617

C	0. -0.478673	-1.360510	1.237391
C	0.278100	-1.125392	-0.044437
C	-0.549948	-1.503772	-1.242943
C	-1.865200	-1.658726	-1.178677
N	-2.551797	-1.559601	0.063092
H	1.227777	-1.670973	-0.042008
H	-2.367010	-1.677855	2.168324
H	0.069290	-1.403866	2.169609
H	-0.052407	-1.649592	-2.192233
S	-4.177792	-1.343686	0.093945
O	-4.737803	-1.855181	-1.129100
O	-4.670319	-1.704493	1.397299
C	-4.335577	0.501766	-0.012420
F	-5.610388	0.837655	-0.040643
F	-3.734927	0.926066	-1.110340
F	-3.754394	1.042852	1.046639
P	0.774609	0.620814	-0.113179
O	1.690857	1.069864	0.975229
O	1.337305	0.766357	-1.591269
O	-0.601650	1.437843	-0.123040
C	3.980412	1.433192	1.673923
H	4.815890	1.157080	1.042986
H	3.421616	2.323142	1.419338
C	3.789115	0.800774	2.947244
H	2.778182	0.901823	3.332034
H	4.193542	-0.205632	3.005611
H	4.440167	1.458288	3.570796
C	-0.947192	2.392107	0.910334
H	-0.031289	2.777031	1.360407
H	-1.513730	1.857433	1.674048
C	-1.760196	3.495959	0.274378
H	-2.081753	4.202525	1.041541
H	-1.164174	4.030860	-0.466514
H	-2.644078	3.089059	-0.216313
C	2.262211	1.827411	-1.930443
H	2.528612	1.635020	-2.967667
H	3.153748	1.705158	-1.312200
C	1.636326	3.197273	-1.762047
H	0.702976	3.264255	-2.322999
H	1.432910	3.412569	-0.709726
H	2.324591	3.957902	-2.134092
I	4.279512	-1.246028	-0.278653
H	-2.484988	-1.930571	-2.020757

$TS_{7/8}$

Symbol	X	Y	Z
C	0. -2.134679	0.145924	-1.981305
C	0. -1.376500	1.220099	-2.162798
C	-1.335896	2.328900	-1.139037
C	-2.600917	2.349885	-0.316939
C	-3.340835	1.259161	-0.167182
N	-3.030994	0.073765	-0.884126
H	-1.163932	3.299020	-1.613526
H	-2.184869	-0.685396	-2.669948
H	-0.765139	1.292237	-3.052229
H	-2.897847	3.266490	0.173284
S	-3.621840	-1.377942	-0.384887
O	-4.806133	-1.148702	0.399428
O	-3.571733	-2.300921	-1.487527
C	-2.316306	-1.940587	0.809402
F	-2.655627	-3.115586	1.304311
F	-2.208226	-1.058235	1.783969
F	-1.166121	-2.044364	0.164827
P	0.075695	2.024236	-0.040835
O	1.417334	2.061683	-0.890110
O	0.075718	3.285358	0.916823
C	1.860480	3.256368	-1.591429
H	1.277313	3.342950	-2.510280
H	1.667671	4.126931	-0.960126
C	3.332504	3.090872	-1.879472
H	3.893327	2.999584	-0.948624
H	3.502702	2.201222	-2.486474
H	3.696829	3.962158	-2.425315
C	1.015742	3.354268	2.029244
H	2.017149	3.134482	1.650005
H	0.731524	2.590426	2.754060
C	0.932943	4.745734	2.605343
H	1.210745	5.486789	1.855065
H	-0.079243	4.952731	2.953985
H	1.617583	4.829791	3.450363
H	-4.242671	1.213037	0.426173
O	0.012153	0.700823	0.670853
C	1.463248	-0.650971	0.613342
H	1.017359	-1.042800	-0.284801
H	2.205139	0.122253	0.500877
C	1.204063	-1.285213	1.940499
H	0.343075	-0.787720	2.390833
H	0.970908	-2.341930	1.826699

H	2.060675	-1.169626	2.601216
I	3.626050	-2.296120	-0.177045

ipr-o-10

Symbol	X	Y	Z
C	-0.491728	0.301039	-0.747160
C	-0.568928	0.527529	-2.235634
C	-1.330475	1.504401	-2.732970
C	-2.055574	2.411227	-1.846246
C	-1.767245	2.447403	-0.545363
N	-0.805852	1.541561	-0.011737
H	-1.400284	1.654009	-3.803065
H	0.509373	-0.054691	-0.462777
H	-0.007023	-0.146104	-2.868939
H	-2.813847	3.073864	-2.239198
S	0.210684	2.077713	1.154277
O	-0.489232	3.023224	1.986812
O	0.937068	0.953278	1.686315
C	1.469890	3.065382	0.211035
F	0.892165	4.127882	-0.320229
F	1.969148	2.306224	-0.755639
F	2.442287	3.440216	1.020617
P	-1.603333	-1.065500	-0.237582
O	-1.494376	-1.178678	1.351750
O	-3.045206	-0.427857	-0.410191
C	-0.385187	-1.872490	2.003158
H	0.533902	-1.575024	1.487156
C	-0.363972	-1.371224	3.431991
H	-1.291736	-1.651578	3.936357
H	0.472645	-1.826200	3.966104
H	-0.250395	-0.288837	3.457981
C	-4.242492	-1.229158	-0.169953
H	-3.977575	-2.037364	0.518974
C	-5.248135	-0.305120	0.482824
H	-6.172116	-0.847750	0.689831
H	-4.853134	0.089393	1.419636
H	-5.473358	0.528775	-0.185768
O	-1.325826	-2.310618	-0.992339
H	-2.218466	3.116290	0.173428
N	2.420787	-1.387034	-0.455289
C	2.120352	-2.812316	-0.333616
H	2.109032	-3.048353	0.735693
H	1.098109	-2.970445	-0.696718
C	2.620863	-0.894693	-1.813569
H	1.960361	-1.477802	-2.464383
H	2.264266	0.142035	-1.860080
C	3.291898	-0.824614	0.571700
H	3.464496	0.224182	0.308735
H	2.731013	-0.806953	1.512903
C	4.630916	-1.526610	0.831254
H	5.186203	-0.976947	1.595409
H	4.471257	-2.540056	1.206647
H	5.250945	-1.588647	-0.063845
C	3.046602	-3.797566	-1.057019
H	2.721942	-4.819647	-0.846527
H	3.001443	-3.655245	-2.139180
H	4.086360	-3.696819	-0.742386
C	4.045266	-0.910426	-2.382192
H	4.026497	-0.554450	-3.415170
H	4.696099	-0.241588	-1.814719
H	4.485004	-1.908316	-2.370563
C	-0.594651	-3.374045	1.921777
H	-1.538607	-3.638528	2.405105
H	-0.615521	-3.714994	0.887513
H	0.217153	-3.882199	2.447031
C	-4.710917	-1.798262	-1.494693
H	-4.939315	-0.983781	-2.185785
H	-3.938025	-2.430530	-1.933129
H	-5.612893	-2.394746	-1.345232

ipr-o-11

Symbol	X	Y	Z
C	0.282494	-1.238206	-0.936367
C	-0.063356	-1.025582	-2.277125
C	-0.973267	-1.863614	-2.896314
C	-1.295037	-3.083843	-2.220387
C	-1.005974	-3.254199	-0.907876
N	-0.588679	-2.086319	-0.177746
H	-1.244589	-1.722838	-3.933078
H	0.248509	1.824595	0.728338
H	0.453248	-0.256487	-2.845862
H	-1.672205	-3.928314	-2.787945
S	-1.790292	-1.325384	0.726012
O	-2.410947	-2.326212	1.575335
O	-1.204056	-0.117309	1.290742
C	-3.233893	-0.667344	-0.273343
F	-3.865770	-1.645989	-0.896398
F	-2.822932	0.229560	-1.161057

F	-4.080565	-0.068426	0.560106
P	1.669209	-0.582459	-0.107845
O	2.062042	-1.648502	1.023262
O	2.857516	-0.687879	-1.178777
C	1.721450	-1.441545	2.421653
H	0.971982	-0.649026	2.475818
C	1.136564	-2.744418	2.929282
H	1.873176	-3.546393	2.839707
H	0.856472	-2.643548	3.979827
H	0.250054	-3.011487	2.351435
C	4.091321	0.050848	-0.962616
H	4.202716	0.227490	0.111547
C	5.216822	-0.833099	-1.459958
H	6.175943	-0.330543	-1.321923
H	5.231937	-1.776648	-0.913048
H	5.082803	-1.044128	-2.523284
O	1.613567	0.803213	0.476505
H	-1.077752	-4.179358	-0.354429
N	-0.250327	2.698113	0.447283
C	0.800616	3.753282	0.662530
H	0.793039	3.976657	1.730307
H	1.741221	3.251505	0.433603
C	-0.546441	2.400087	-0.998450
H	0.398237	2.522790	-1.530243
H	-0.791781	1.338978	-1.018089
C	-1.459676	2.820296	1.331535
H	-2.189370	2.108520	0.949602
H	-1.142973	2.458231	2.310258
C	-2.049812	4.214299	1.459929
H	-2.922787	4.140762	2.109915
H	-1.351999	4.909778	1.927162
H	-2.377116	4.626991	0.506930
C	0.664890	5.016102	-0.171781
H	1.485555	5.680152	0.103192
H	0.758861	4.805557	-1.237620
H	-0.268712	5.547883	0.003343
C	-1.664330	3.201960	-1.640115
H	-1.758617	2.859408	-2.671986
H	-2.621835	3.021663	-1.150011
H	-1.469007	4.273285	-1.658211
C	2.979476	-1.016548	3.153852
H	3.749019	-1.785514	3.051335
H	3.357411	-0.079312	2.741477
H	2.768704	-0.871820	4.215488
C	3.999984	1.375701	-1.696324
H	3.855694	1.197428	-2.764697
H	3.161045	1.959041	-1.314456
H	4.920131	1.947316	-1.558806

ipr-o-3

Symbol	X	Y	Z
C	0.516171	-1.018050	0.624838
C	0.222664	-2.279458	0.156111
C	-0.029348	-2.459346	-1.199247
C	0.001481	-1.369570	-2.064345
C	0.276028	-0.121769	-1.558139
N	0.542969	0.015258	-0.241166
H	-0.257939	-3.446054	-1.581020
H	0.738530	-0.790685	1.657343
H	0.202298	-3.104593	0.853835
H	-0.201326	-1.474371	-3.120139
S	0.702463	1.656481	0.418267
O	3.131860	-1.416622	-1.122117
O	-0.313465	2.407027	-0.263537
O	0.775036	1.511433	1.839386
C	2.370919	2.262249	-0.290388
F	2.102560	3.423450	-0.845861
F	2.794936	1.420358	-1.198846
F	3.222175	2.414837	0.681966
S	3.712603	-1.279001	0.213653
O	3.093706	-0.220402	1.028716
O	3.958581	-2.530057	0.926477
C	5.404335	-0.614573	-0.099120
F	6.089836	-1.439387	-0.886783
F	6.067944	-0.477902	1.045854
F	5.342024	0.575556	-0.690702
P	-2.887368	-0.123878	-0.140993
O	-3.706529	-0.393591	1.265828
O	-3.792412	1.140494	-0.660217
O	-3.476809	-1.258408	-1.137169
C	-3.000729	-1.028751	2.349592
H	-1.966530	-0.660335	2.349440
C	-3.685283	-0.598863	3.631723
H	-4.725518	-0.932573	3.625520
H	-3.182386	-1.037800	4.495311
H	-3.666185	0.487812	3.726854
C	-4.902935	-1.486274	-1.304649
H	-5.427024	-0.960810	-0.502101

C	-5.321334	-0.928703	-2.652386
H	-6.387845	-1.099354	-2.814856
H	-5.122727	0.142115	-2.693732
H	-4.764183	-1.426972	-3.449489
C	-3.712981	2.372567	0.092845
H	-2.856481	2.314378	0.777278
C	-3.484055	3.498761	-0.896354
H	-3.422957	4.455049	-0.372947
H	-2.555993	3.339845	-1.446410
H	-4.314343	3.541878	-1.605522
H	0.275512	0.776099	-2.161621
C	-4.994525	2.534671	0.891279
H	-5.848420	2.569710	0.209985
H	-5.121614	1.694995	1.574747
H	-4.968621	3.463934	1.464624
C	-3.007774	-2.535599	2.151894
H	-2.554170	-2.806829	1.195103
H	-2.454388	-3.030485	2.952616
H	-4.037632	-2.901572	2.157979
C	-5.132215	-2.979998	-1.183443
H	-4.807463	-3.340310	-0.206395
H	-6.192546	-3.209231	-1.307685
H	-4.568894	-3.507132	-1.957237

ipr-o-4

Symbol	X	Y	Z
C	0.517777	0.438147	-0.220807
C	0.215136	1.499155	-1.246227
C	0.163299	1.177262	-2.539316
C	0.310390	-0.208858	-2.972200
C	0.246826	-1.197857	-2.077814
N	0.137621	-0.894102	-0.696883
H	-0.054634	1.934372	-3.281701
H	-0.006810	0.655145	0.710972
H	0.040046	2.502127	-0.879928
H	0.422740	-0.449706	-4.019824
S	-0.547698	-1.999919	0.324819
O	-3.239963	1.677688	-1.560215
O	-0.005258	-3.294749	-0.012082
O	-0.462641	-1.466212	1.661224
C	-2.383240	-2.125341	-0.142235
F	-2.626229	-3.389546	-0.444783
F	-2.648897	-1.375971	-1.190412
F	-3.117574	-1.781792	0.890044
S	-3.123133	1.695952	-0.104314
O	-2.159926	0.744083	0.463814
O	-3.082284	3.024784	0.506264
C	-4.747394	1.016686	0.451844
F	-5.728360	1.880336	0.191002
F	-4.738415	0.779699	1.761820
F	-5.025694	-0.123828	-0.176991
P	2.308176	0.392860	0.138727
O	2.636196	-0.690179	1.211210
O	3.132444	-0.002802	-1.121826
O	2.674910	1.848970	0.544817
C	2.327395	-0.678289	2.665302
H	1.311896	-0.295270	2.770832
C	2.396843	-2.126768	3.086558
H	3.406397	-2.512812	2.931348
H	2.151233	-2.201729	4.147011
H	1.686033	-2.726385	2.519154
C	3.971639	2.524856	0.260421
H	4.747166	1.758625	0.328327
C	3.891885	3.108327	-1.133421
H	4.824659	3.630296	-1.353418
H	3.743555	2.330210	-1.882102
H	3.069220	3.824276	-1.187388
C	3.732372	-1.331508	-1.410639
H	3.022189	-2.080181	-1.051449
C	3.874852	-1.389372	-2.913576
H	4.302607	-2.353907	-3.191643
H	2.908574	-1.277085	-3.402638
H	4.545538	-0.598316	-3.254690
H	0.279442	-2.252043	-2.320864
C	5.054612	-1.430174	-0.680427
H	5.720566	-0.631245	-1.013814
H	4.921357	-1.367353	0.399311
H	5.518921	-2.389035	-0.917300
C	3.335178	0.210313	3.359650
H	3.228555	1.248365	3.046923
H	3.168401	0.160480	4.436856
H	4.349591	-0.132371	3.145603
C	4.140574	3.566905	1.340599
H	4.197601	3.113530	2.329266
H	5.065905	4.115576	1.158511
H	3.306415	4.270075	1.313816

ipr-o-5

Symbol	X	Y	Z
C	0.489830	-0.337476	-0.554629
C	0.062095	0.023871	-1.951707
C	-0.150748	-0.930649	-2.857554
C	-0.038515	-2.342677	-2.498879
C	0.048365	-2.703247	-1.217423
N	0.117580	-1.713333	-0.205499
H	-0.463950	-0.671558	-3.861006
H	0.061583	0.363253	0.166897
H	-0.066160	1.078907	-2.157623
H	-0.060617	-3.107749	-3.261962
S	-0.338673	-2.094514	1.337367
O	-3.457600	-0.897342	-2.076511
O	0.315279	-3.336061	1.683068
O	-0.188431	-0.905015	2.139222
C	-2.182701	-2.543098	1.320379
F	-2.302652	-3.698614	1.954096
F	-2.622502	-2.683197	0.088572
F	-2.864398	-1.627832	1.967264
S	-3.208060	0.232718	-1.191728
O	-2.251055	0.018544	-0.105108
O	-3.047574	1.525321	-1.881791
C	-4.801240	0.450779	-0.290613
F	-5.795527	0.689483	-1.142034
F	-4.719339	1.475948	0.555533
F	-5.093670	-0.645851	0.402688
P	2.311710	-0.249854	-0.393665
O	2.766691	-0.487450	1.078371
O	2.987935	-1.400036	-1.204366
O	2.719516	1.110710	-1.018530
C	2.592157	0.373431	2.278115
H	1.574809	0.762877	2.241578
C	2.780799	-0.558529	3.450983
H	3.791988	-0.971171	3.446771
H	2.634248	0.000912	4.376385
H	2.055710	-1.370813	3.413905
C	4.094047	1.562730	-1.347082
H	4.747574	1.188523	-0.554606
C	4.482961	0.995170	-2.695927
H	5.467538	1.379829	-2.967553
H	4.524627	-0.093021	-2.678610
H	3.762873	1.315605	-3.451519
C	3.507120	-2.680254	-0.666335
H	2.809162	-3.002948	0.111029
C	3.503600	-3.637845	-1.836142
H	3.855195	-4.613816	-1.497790
H	2.502742	-3.746355	-2.251032
H	4.177047	-3.274773	-2.615255
H	0.086160	-3.727384	-0.868329
C	4.892339	-2.444591	-0.100194
H	5.548928	-2.056319	-0.882198
H	4.870770	-1.745923	0.735414
H	5.297568	-3.395135	0.251320
C	3.608079	1.490374	2.208302
H	3.404005	2.148696	1.363325
H	3.542886	2.090366	3.117703
H	4.617991	1.080537	2.131573
C	4.030152	3.072931	-1.330310
H	3.673196	3.445237	-0.370207
H	5.026559	3.474920	-1.519929
H	3.354540	3.422108	-2.113728
Na	-2.057543	3.373370	-1.052565
I	0.252824	3.265730	0.971153

ipr-o-6

Symbol	X	Y	Z
C	-0.369128	-0.236288	-0.722677
C	-0.341923	-0.107500	-2.222904
C	-0.752477	-1.124240	-2.982432
C	-1.287001	-2.343018	-2.378373
C	-1.628187	-2.360337	-1.089440
N	-1.363751	-1.220890	-0.272123
H	-0.735486	-1.043602	-4.061817
H	-0.565830	0.744448	-0.273501
H	0.017380	0.833117	-2.620204
H	-1.448976	-3.226869	-2.979408
S	-2.484380	-0.780534	0.860427
O	-3.095136	-1.975451	1.378510
O	-1.892153	0.232416	1.695851
C	-3.782051	0.084070	-0.153508
F	-4.319546	-0.781725	-0.992283
F	-3.203490	1.061572	-0.830784
F	-4.707551	0.576602	0.643871
P	1.246066	-0.767117	-0.050461
O	1.276021	-0.652531	1.501903
O	1.519211	-2.269531	-0.358722
O	2.288873	0.100471	-0.802938
C	1.117176	0.566513	2.342383

H 0.342112 1.175782 1.876099
C 0.669826 0.053099 3.689396
H 1.438978 -0.589602 4.122819
H 0.505815 0.901625 4.355762
H -0.262195 -0.502679 3.594561
C 3.766407 -0.031213 -0.716245
H 3.998575 -0.291501 0.320057
C 4.213118 -1.118223 -1.670884
H 5.302843 -1.173557 -1.653030
H 3.809326 -2.090825 -1.392945
H 3.892879 -0.872316 -2.685152
C 1.306193 -3.424099 0.550286
H 0.390032 -3.220035 1.109910
C 1.138898 -4.620609 -0.357852
H 0.958524 -5.507444 0.251673
H 0.300111 -4.482886 -1.038478
H 2.050212 -4.774947 -0.939006
H -2.093286 -3.191508 -0.577632
C 2.500804 -3.539015 1.473619
H 3.410990 -3.676049 0.885732
H 2.604619 -2.657055 2.104561
H 2.368409 -4.412159 2.114939
C 2.438125 1.299623 2.365470
H 2.697192 1.664653 1.370997
H 2.349173 2.166471 3.022753
H 3.228993 0.649167 2.745826
C 4.307650 1.334845 -1.069986
H 3.923344 2.103078 -0.399343
H 5.396134 1.316718 -0.998343
H 4.026800 1.588995 -2.093873
I 0.230469 3.458827 -0.307796

ipr-o-7

Symbol	X	Y	Z
C	-2.275316	2.936056	-0.179655
C	-1.951324	2.199344	0.885224
C	-1.695439	0.724402	0.714536
C	-2.583706	0.998406	-1.599168
H	-1.973187	0.165581	1.611386
H	-2.447224	3.999995	-0.074753
H	-1.852333	2.612148	1.880057
S	-3.388854	-1.145803	-0.258492
O	-3.652275	-1.697293	-1.560733
O	-2.881883	-1.921147	0.843426
C	-4.984065	-0.392500	0.331940
F	-5.839166	-1.349015	0.628961
F	-5.476484	0.378097	-0.619011
F	-4.729670	0.334276	1.408277
P	0.079874	0.367547	0.471201
O	0.824260	1.010130	1.674816
O	0.260049	-1.176050	0.458952
O	0.528169	0.964610	-0.893121
C	2.017945	0.422924	2.355408
H	2.501020	-0.240551	1.634406
C	1.519222	-0.342420	3.558379
H	0.859632	-1.156957	3.255057
H	2.373725	-0.768763	4.086895
H	0.984133	0.324624	4.236882
C	1.668527	1.895915	-1.124749
H	2.482781	1.547456	-0.486391
C	2.034775	1.707863	-2.576214
H	2.890005	2.343435	-2.812427
H	2.312412	0.670289	-2.765598
H	1.196559	1.988175	-3.217379
C	0.892639	-1.986780	-0.618532
H	1.696984	-1.377363	-1.034900
C	-0.166726	-2.292645	-1.651689
H	-0.987722	-2.852646	-1.198687
H	-0.554068	-1.375568	-2.101444
H	0.272616	-2.900103	-2.444841
I	4.639052	-0.600839	-0.433422
H	-2.797956	0.463824	-2.513869
C	1.459663	-3.197744	0.080090
H	1.965906	-3.827082	-0.653981
H	2.186733	-2.893828	0.834441
H	0.660711	-3.774307	0.550353
C	1.229714	3.296399	-0.765610
H	2.061721	3.980835	-0.941423
H	0.386241	3.601740	-1.388749
H	0.943045	3.367405	0.285409
C	2.920361	1.588334	2.676888
H	3.221892	2.108442	1.766239
H	2.415870	2.288063	3.345931
H	3.818944	1.213058	3.169829
N	-2.411291	0.175937	-0.446717
C	-2.461852	2.321892	-1.491848
H	-2.550837	2.935727	-2.376990

ipr-o-8

Symbol	X	Y	Z
C	-1.261310	-0.348060	-0.873383
C	-0.342368	-1.468832	-1.291624
C	0.043536	-2.393065	-0.409834
C	-0.481716	-2.394677	0.953625
C	-1.524426	-1.626633	1.269481
N	-2.065961	-0.733662	0.302730
H	0.721207	-3.183655	-0.707631
H	-1.933179	-0.084791	-1.692558
H	-0.003937	-1.468654	-2.319535
H	-0.053518	-3.040621	1.707019
S	-3.677652	-0.435751	0.310933
O	-4.145110	-0.404798	1.673214
O	-3.945765	0.619134	-0.634920
C	-4.393261	-1.975313	-0.446446
F	-4.163439	-3.002898	0.351527
F	-3.805625	-2.182935	-1.614492
F	-5.691910	-1.825136	-0.621418
P	-0.295153	1.170661	-0.502930
O	-1.306508	2.241642	0.101631
O	0.538292	0.735363	0.774621
C	-2.086971	3.170613	-0.715912
H	-2.510233	2.605728	-1.552229
C	-3.196207	3.669443	0.185743
H	-2.767204	4.206544	1.034768
H	-3.840330	4.353662	-0.369503
H	-3.797145	2.837852	0.550876
C	0.702547	1.501337	2.007831
H	0.665421	2.561972	1.749555
C	2.068524	1.131459	2.545879
H	2.247512	1.652105	3.488347
H	2.116560	0.054568	2.725248
H	2.853767	1.405503	1.840574
O	0.485564	1.591095	-1.692090
C	3.421389	0.173232	-0.861791
H	2.600352	-0.000266	-0.169886
C	3.127525	-0.445340	-2.214177
H	2.874484	-1.502457	-2.135622
H	2.265019	0.092420	-2.622295
H	3.975272	-0.329503	-2.892458
I	5.061888	-0.916541	0.121472
H	-2.012819	-1.608360	2.233836
C	-1.190923	4.291573	-1.211496
H	-0.388410	3.908170	-1.840369
H	-1.786254	5.001868	-1.788432
H	-0.755762	4.816371	-0.357490
C	-0.419428	1.145814	2.965015
H	-1.393358	1.328841	2.509710
H	-0.345374	0.092721	3.244689
H	-0.336077	1.752101	3.869069
C	3.774240	1.645887	-0.957728
H	4.001146	2.084155	0.014474
H	4.633543	1.795812	-1.614781
H	2.910801	2.166951	-1.384766

ipr-o-9

Symbol	X	Y	Z
C	-0.103468	-0.450032	-1.222185
C	-0.282853	-1.717738	-2.016261
C	-0.546877	-2.872035	-1.401809
C	-0.740518	-2.923959	0.045757
C	-0.898054	-1.797068	0.739797
N	-0.791190	-0.540473	0.081228
H	-0.657154	-3.784104	-1.974736
H	-0.509262	0.399949	-1.774852
H	-0.171655	-1.644153	-3.090199
H	-0.787666	-3.873146	0.560591
S	-1.735714	0.698474	0.590704
O	-1.810479	0.675459	2.029474
O	-1.379147	1.879736	-0.156020
C	-3.425569	0.219980	-0.020099
F	-3.840206	-0.854406	0.627438
F	-3.345692	-0.045734	-1.314199
F	-4.268014	1.215716	0.178571
P	1.673168	-0.054377	-0.953027
O	1.693675	1.162864	0.075576
O	2.171797	-1.259546	-0.051612
C	1.703030	2.568395	-0.322770
H	0.945165	2.697704	-1.100905
C	1.313991	3.344667	0.917944
H	2.059872	3.186829	1.700436
H	1.273673	4.410313	0.685372
H	0.337700	3.027026	1.281377
C	2.972687	-1.121821	1.162581
H	3.563690	-0.208081	1.072147
C	3.878004	-2.333887	1.211357
H	4.504487	-2.290215	2.104041

H	3.277256	-3.245181	1.250006
H	4.520921	-2.370970	0.331544
O	2.378947	0.150038	-2.237325
H	-1.098884	-1.743914	1.800889
C	3.079491	2.947805	-0.837634
H	3.337805	2.376349	-1.727812
H	3.093708	4.011715	-1.082136
H	3.825532	2.759881	-0.061513
C	2.036392	-1.026837	2.351372
H	1.342354	-0.193147	2.230895
H	1.469044	-1.954907	2.450769
H	2.614969	-0.871986	3.264298

ipr-o-TS$_{10/11}$

Symbol	X	Y	Z
C	-0.226724	0.050383	-0.827643
C	-0.093672	0.094690	-2.315071
C	-0.393281	1.177636	-3.046689
C	-0.989206	2.343343	-2.402612
C	-1.003459	2.446590	-1.074429
N	-0.375463	1.426142	-0.289492
H	-0.256584	1.175667	-4.120544
H	1.032758	-0.712779	-0.471192
H	0.241449	-0.813625	-2.805109
H	-1.482020	3.102715	-2.995257
S	0.376725	1.907412	1.065670
O	-0.372407	2.982440	1.676988
O	0.808834	0.760792	1.826291
C	1.946373	2.718477	0.492532
F	1.664001	3.813810	-0.192809
F	2.613680	1.877086	-0.291807
F	2.702771	3.029445	1.531888
P	-1.672611	-0.915664	-0.380948
O	-1.855144	-0.844775	1.216189
O	-2.932467	-0.031889	-0.826307
C	-1.100731	-1.679045	2.130044
H	-0.052092	-1.647049	1.818588
C	-1.244861	-1.037439	3.495477
H	-2.295163	-1.049302	3.797276
H	-0.665735	-1.597562	4.232208
H	-0.887949	-0.008842	3.472106
C	-4.276119	-0.419597	-0.428642
H	-4.234329	-1.422561	0.008156
C	-4.760332	0.575244	0.608538
H	-5.773084	0.322935	0.929520
H	-4.099776	0.565573	1.476138
H	-4.769542	1.580100	0.179414
O	-1.661814	-2.295511	-0.940164
H	-1.475069	3.246289	-0.520916
N	2.078637	-1.448934	-0.475455
C	1.505779	-2.816386	-0.630347
H	1.145808	-3.117437	0.357382
H	0.618416	-2.712583	-1.258378
C	2.823875	-0.967962	-1.675401
H	2.353952	-1.428402	-2.545091
H	2.633718	0.104234	-1.747572
C	2.777632	-1.230627	0.821774
H	3.266850	-0.257585	0.756113
H	1.995574	-1.129646	1.572142
C	3.759621	-2.301805	1.283790
H	4.186222	-1.970278	2.232421
H	3.255564	-3.251511	1.468819
H	4.580077	-2.469505	0.588302
C	2.417952	-3.886948	-1.218858
H	1.855733	-4.821507	-1.261114
H	2.717196	-3.641502	-2.238999
H	3.314171	-4.060499	-0.625590
C	4.326990	-1.213653	-1.710534
H	4.712124	-0.794433	-2.641637
H	4.838538	-0.712151	-0.887980
H	4.579118	-2.273514	-1.689283
C	-1.620856	-3.106738	2.106352
H	-2.682728	-3.113142	2.365842
H	-1.495795	-3.555877	1.122020
H	-1.079998	-3.702638	2.844688
C	-5.124126	-0.442176	-1.684423
H	-5.127137	0.547506	-2.146964
H	-4.728169	-1.163805	-2.400319
H	-6.151973	-0.716820	-1.439629

ipr-o-TS$_{3/4}$

Symbol	X	Y	Z
C	0.181956	0.675543	-0.078939
C	0.253986	1.909730	-0.806454
C	0.143531	1.901880	-2.157655
C	-0.088563	0.678719	-2.859568
C	-0.324208	-0.449912	-2.159585
N	-0.318318	-0.409695	-0.771974

H	0.210436	2.828478	-2.713119
H	-0.047503	0.681442	0.976190
H	0.438815	2.812827	-0.242309
H	-0.120685	0.647844	-3.938811
S	-0.586711	-1.862899	0.095722
O	-2.983773	1.392339	-1.257218
O	0.104456	-2.895220	-0.634564
O	-0.305434	-1.581594	1.476073
C	-2.440630	-2.230660	-0.130343
F	-2.508893	-3.470404	-0.573480
F	-2.955058	-1.416592	-1.018356
F	-3.042957	-2.139840	1.024461
S	-3.118395	1.512459	0.192439
O	-2.402713	0.485611	0.965684
O	-2.993468	2.867751	0.726827
C	-4.883839	1.082513	0.507441
F	-5.693082	1.900682	-0.162554
F	-5.166295	1.185316	1.804309
F	-5.140774	-0.166065	0.123090
P	2.506622	0.309979	0.218877
O	3.264435	-0.728522	1.185374
O	3.287168	0.067264	-1.160356
O	2.967904	1.782276	0.603550
C	2.740682	-1.052736	2.511349
H	1.710854	-0.689021	2.572829
C	2.753892	-2.561918	2.623137
H	3.775634	-2.934434	2.519737
H	2.366191	-2.865831	3.597001
H	2.131812	-3.005997	1.844522
C	4.328724	2.296469	0.397002
H	5.004236	1.437902	0.356689
C	4.345594	3.053620	-0.916020
H	5.340231	3.470005	-1.086954
H	4.092134	2.393836	-1.745632
H	3.626139	3.874795	-0.876372
C	3.235548	-1.211374	-1.867304
H	2.286639	-1.702631	-1.619367
C	3.278452	-0.874883	-3.342779
H	3.223059	-1.790908	-3.933233
H	2.446572	-0.226392	-3.616901
H	4.215734	-0.364470	-3.575800
H	-0.549608	-1.404880	-2.614121
C	4.400777	-2.076798	-1.426550
H	5.339478	-1.555696	-1.627807
H	4.341426	-2.305680	-0.363384
H	4.392482	-3.010300	-1.992490
C	3.607211	-0.352138	3.537508
H	3.555174	0.729692	3.407802
H	3.263819	-0.597712	4.543978
H	4.645391	-0.673833	3.431128
C	4.647627	3.169257	1.591331
H	4.626775	2.593182	2.516266
H	5.643574	3.599642	1.472302
H	3.922269	3.982372	1.662306

ipr-o-TS$_{7/8}$

Symbol	X	Y	Z
C	1.582995	0.171800	-0.866361
C	2.331425	0.916719	-1.938716
C	3.455356	1.565364	-1.624011
C	4.005222	1.490113	-0.273374
C	3.570036	0.557679	0.577868
N	2.496733	-0.292713	0.189453
H	3.996519	2.126077	-2.375532
H	1.051551	-0.687708	-1.280129
H	1.913692	0.916510	-2.936764
H	4.800182	2.155547	0.033366
S	2.523066	-1.875622	0.630444
O	3.148993	-1.981199	1.922818
O	1.242023	-2.456912	0.322787
C	3.714911	-2.621483	-0.583683
F	4.905060	-2.072067	-0.418806
F	3.279217	-2.375319	-1.808940
F	3.790457	-3.923802	-0.390668
P	0.279110	1.195095	-0.074648
O	0.900049	2.607483	0.277216
O	-0.678251	1.456496	-1.303584
C	1.139357	3.071600	1.647959
H	1.510908	2.218629	2.222530
C	2.199549	4.145070	1.538960
H	1.831806	4.969532	0.924329
H	2.433562	4.528053	2.533592
H	3.109005	3.745634	1.091361
C	-1.576553	2.619791	-1.377853
H	-1.748042	2.974251	-0.356781
C	-2.869878	2.125256	-1.986694
H	-3.569445	2.957948	-2.078016
H	-2.680090	1.717640	-2.981794

H	-3.332114	1.351185	-1.370918
O	-0.346053	0.516049	1.107105
C	-2.244182	-0.470312	1.088389
H	-2.251320	-0.207427	0.043781
C	-1.727836	-1.790646	1.513828
H	-1.335522	-2.368598	0.681943
H	-0.932829	-1.617268	2.243475
H	-2.523956	-2.341095	2.018152
I	-4.821839	-1.440320	-0.212701
H	3.971882	0.376653	1.564936
C	-0.164568	3.582423	2.227878
H	-0.910334	2.787984	2.273080
H	0.005794	3.955639	3.239348
H	-0.545473	4.402868	1.614559
C	-0.886573	3.688106	-2.200081
H	0.051951	3.987770	-1.733298
H	-0.684048	3.307004	-3.203393
H	-1.535059	4.562186	-2.282119
C	-2.860231	0.436133	2.086312
H	-3.182608	1.377980	1.647041
H	-3.715062	-0.065153	2.543327
H	-2.127184	0.617314	2.875995

ipr-p-10

Symbol	X	Y	Z
C	-1.510747	-1.161320	1.193656
C	-0.262559	-0.737235	1.345266
C	0.605552	-0.288593	0.201944
C	-0.124212	-0.364409	-1.109912
C	-1.374225	-0.796060	-1.226336
N	-2.120126	-1.228446	-0.091942
H	1.503201	-0.935572	0.150994
H	-2.125096	-1.521403	2.005857
H	0.162940	-0.743271	2.341081
H	0.404048	-0.087042	-2.014063
S	-3.745689	-1.419453	-0.216704
O	-4.058230	-1.867978	-1.547983
O	-4.219769	-2.083020	0.969549
C	-4.389886	0.322921	-0.110380
F	-5.695295	0.303558	0.070887
F	-4.102178	0.959244	-1.232404
F	-3.807497	0.938991	0.907584
P	1.326687	1.351889	0.510290
O	1.630295	1.617859	1.938671
O	2.563049	1.335953	-0.488953
O	0.385048	2.452227	-0.179439
C	-0.902746	2.785434	0.415013
H	-1.290257	1.889936	0.914782
C	-1.812052	3.161708	-0.735902
H	-2.813214	3.386837	-0.363912
H	-1.419294	4.046148	-1.242607
H	-1.877946	2.341847	-1.452104
C	3.483258	2.464700	-0.555379
H	3.060487	3.289076	0.026944
C	3.592255	2.862048	-2.012599
H	3.972720	2.021093	-2.597249
H	2.616134	3.152684	-2.402936
H	4.280048	3.702780	-2.119872
N	3.017160	-2.373714	0.048602
C	2.520054	-3.511283	-0.728545
H	2.463994	-3.199739	-1.771892
H	3.235728	-4.352060	-0.681061
C	4.260137	-1.840772	-0.513351
H	4.584580	-1.022348	0.130004
H	5.055283	-2.608261	-0.489381
C	3.174784	-2.727825	1.461561
H	2.215001	-3.110709	1.812635
H	3.908546	-3.546352	1.574819
C	3.565723	-1.552657	2.353598
H	3.440028	-1.836067	3.400288
H	4.605101	-1.250146	2.220295
H	2.928395	-0.684750	2.160322
C	1.132155	-3.987158	-0.306466
H	0.746034	-4.685119	-1.051574
H	1.135829	-4.503431	0.654005
H	0.440467	-3.142210	-0.242569
C	4.105791	-1.286588	-1.926107
H	5.001407	-0.721384	-2.194980
H	3.977706	-2.067028	-2.676903
H	3.251942	-0.606002	-1.964363
H	-1.887368	-0.893478	-2.172565
C	4.798706	2.019808	0.051239
H	5.213985	1.195102	-0.532954
H	5.512465	2.845773	0.046713
H	4.651157	1.686457	1.080100
C	-0.717717	3.904003	1.422528
H	-0.041744	3.594651	2.219615
H	-0.302665	4.782361	0.922675

H -1.682187 4.172872 1.857830

ipr-p-11

Symbol	X	Y	Z
C	-1.364931	-1.626748	1.130390
C	-0.450810	-0.658888	1.367842
C	0.077938	0.137211	0.301681
C	-0.189822	-0.323737	-1.028979
C	-1.098871	-1.298981	-1.261259
N	-1.986858	-1.673407	-0.178633
H	1.207627	-1.931630	-0.036978
H	-1.700857	-2.361677	1.846853
H	-0.041794	-0.555012	2.368720
H	0.416788	0.032820	-1.856925
S	-3.529278	-1.062154	-0.266194
O	-4.001513	-1.208558	-1.627448
O	-4.287162	-1.574783	0.856261
C	-3.519922	0.787150	0.009363
F	-4.769363	1.229728	-0.075478
F	-2.789520	1.394400	-0.913897
F	-3.044614	1.082322	1.209290
P	1.256757	1.370510	0.610463
O	1.527758	1.631999	2.053059
O	2.560492	0.945087	-0.238289
O	0.862270	2.699756	-0.224632
C	-0.330196	3.434346	0.142196
H	-1.039222	2.729619	0.591561
C	-0.913647	3.982105	-1.144858
H	-1.839554	4.522354	-0.937826
H	-0.204377	4.669315	-1.612602
H	-1.129423	3.169257	-1.839167
C	3.659810	1.879775	-0.406717
H	3.473624	2.751629	0.227572
C	3.695496	2.300111	-1.862927
H	3.843349	1.422897	-2.497859
H	2.756158	2.781969	-2.135781
H	4.517163	2.998121	-2.036120
N	1.904662	-2.689514	0.029157
C	1.413953	-3.837420	-0.814741
H	1.179253	-3.413353	-1.789146
H	2.263736	-4.514749	-0.921401
C	3.209732	-2.150852	-0.495604
H	3.427548	-1.262794	0.091177
H	3.958311	-2.918242	-0.287815
C	2.012328	-3.062494	1.484011
H	0.993804	-3.247927	1.819787
H	2.573423	-3.998400	1.520500
C	2.664995	-1.986537	2.337005
H	2.486656	-2.234547	3.383715
H	3.742427	-1.935593	2.183789
H	2.235418	-0.999709	2.148413
C	0.199873	-4.556085	-0.255117
H	-0.124510	-5.280894	-1.002742
H	0.423367	-5.100793	0.661546
H	-0.624676	-3.865301	-0.074478
C	3.167938	-1.780163	-1.965678
H	4.108577	-1.284152	-2.209977
H	3.069957	-2.644210	-2.621540
H	2.362263	-1.072176	-2.164373
H	-1.251258	-1.803272	-2.203969
C	4.924914	1.179583	0.050665
H	5.130960	0.315172	-0.585933
H	5.775953	1.860314	-0.011913
H	4.821074	0.841749	1.083686
C	0.032810	4.519691	1.140530
H	0.474507	4.080628	2.035509
H	0.752393	5.208547	0.691135
H	-0.859012	5.082829	1.423398

ipr-p-12

Symbol	X	Y	Z
C	1.600890	-2.154174	-0.155216
C	0.587305	-1.307581	-0.433721
C	0.317677	-0.174474	0.402542
C	0.916899	-0.204773	1.707180
C	1.923367	-1.058594	1.986974
N	2.535651	-1.766399	0.880676
H	-2.657995	-1.096211	-0.721431
H	1.780099	-3.103167	-0.637435
H	-0.083472	-1.541366	-1.255289
H	0.472984	0.370738	2.513813
S	3.996519	-1.183023	0.368442
O	4.829578	-0.947397	1.530163
O	4.459502	-1.999235	-0.735486
C	3.807557	0.519469	-0.382127
F	4.995550	0.917343	-0.825783
F	3.383121	1.385682	0.525949
F	2.961212	0.500140	-1.400891

P	-0.993919	0.874763	0.016966
O	-1.911702	0.301832	-1.039116
O	-1.717093	1.204992	1.407691
O	-0.523125	2.358221	-0.406910
C	0.205725	2.522932	-1.650865
H	0.642265	1.555746	-1.924502
C	1.319142	3.514929	-1.383176
H	1.913177	3.663397	-2.287116
H	0.897783	4.476084	-1.079322
H	1.971332	3.148675	-0.590133
C	-2.432376	2.438609	1.685895
H	-1.732599	3.260900	1.524110
C	-2.828415	2.360338	3.145775
H	-3.504224	1.516563	3.305563
H	-1.947091	2.230100	3.774976
H	-3.339058	3.278176	3.442278
N	-3.284441	-1.905192	-0.445197
C	-3.584761	-1.716406	1.015896
H	-3.819622	-0.658671	1.134683
H	-4.479612	-2.302909	1.235578
C	-4.523536	-1.760468	-1.276941
H	-4.207471	-1.819902	-2.316262
H	-5.160530	-2.620082	-1.056216
C	-2.558993	-3.183103	-0.729860
H	-1.637900	-3.148898	-0.149870
H	-3.176503	-4.000904	-0.351533
C	-2.229275	-3.354712	-2.204274
H	-1.486516	-4.146675	-2.302104
H	-3.098575	-3.635302	-2.797549
H	-1.802278	-2.438025	-2.619703
C	-2.439521	-2.089368	1.942984
H	-2.694493	-1.735063	2.942581
H	-2.287089	-3.167059	1.997497
H	-1.504605	-1.607526	1.647741
C	-5.235696	-0.439373	-1.038141
H	-6.008109	-0.322682	-1.798402
H	-5.718474	-0.395969	-0.061988
H	-4.531665	0.390303	-1.131056
H	2.312727	-1.272532	2.970860
C	-3.627041	2.586108	0.760198
H	-3.316669	2.610099	-0.286224
H	-4.321122	1.754068	0.904300
H	-4.151946	3.516168	0.987180
C	-0.763628	2.980899	-2.724826
H	-1.210609	3.935362	-2.436109
H	-0.238542	3.113943	-3.672942
H	-1.555732	2.243354	-2.857919

ipr-p-13

Symbol	X	Y	Z
C	1.804768	-0.057947	-3.147865
C	0.710711	-0.031564	-2.288228
C	0.633338	-1.004513	-1.295670
C	1.657784	-1.938162	-1.185734
C	2.709923	-1.865682	-2.093186
N	2.780240	-0.962151	-3.070276
H	-1.866221	1.804687	0.067442
H	1.890292	0.679945	-3.938332
H	-0.051315	0.731439	-2.398181
H	1.644132	-2.698447	-0.415273
S	3.482444	0.883317	-0.346784
O	4.952790	0.583951	-0.203332
O	3.150870	2.322885	-0.049369
C	2.892820	0.126748	1.275249
F	3.414268	0.731347	2.344138
F	3.212128	-1.174769	1.351936
F	1.550123	0.208210	1.388633
P	-0.811732	-1.075067	-0.232086
O	-1.303496	0.255871	0.224386
O	-1.821398	-1.883119	-1.155678
O	-0.465735	-2.057164	0.964671
C	-0.593151	-1.665517	2.372854
H	-0.392709	-0.595428	2.432625
C	0.463736	-2.445533	3.121355
H	0.426041	-2.187051	4.181070
H	0.287210	-3.518173	3.016862
H	1.453568	-2.206956	2.734079
C	-3.059764	-2.469170	-0.648222
H	-2.827312	-2.926339	0.317405
C	-3.453230	-3.538770	-1.643868
H	-3.640884	-3.088201	-2.620697
H	-2.661497	-4.281784	-1.741127
H	-4.364093	-4.036172	-1.306928
N	-2.272186	2.764037	-0.000504
C	-3.369212	2.705687	-1.023408
H	-4.240965	2.275851	-0.532309
H	-3.604473	3.737196	-1.292115
C	-2.804702	3.123151	1.357200

H	-1.959072	3.463903	1.951207
H	-3.484741	3.964942	1.214302
C	-1.165209	3.690177	-0.417109
H	-1.067311	3.607616	-1.498161
H	-1.500453	4.702234	-0.182692
C	0.151523	3.341821	0.254329
H	0.928277	4.005773	-0.123627
H	0.112504	3.461855	1.337878
H	0.460540	2.320149	0.028224
C	-2.982335	1.868285	-2.231138
H	-3.843291	1.797447	-2.896301
H	-2.161866	2.307359	-2.799065
H	-2.704414	0.857813	-1.925470
C	-3.479236	1.944749	2.038389
H	-3.783150	2.249634	3.039866
H	-4.372254	1.610821	1.508524
H	-2.785007	1.107062	2.125881
H	3.527723	-2.575875	-2.030555
C	-4.112117	-1.387033	-0.488719
H	-3.778364	-0.609181	0.200647
H	-4.326375	-0.930677	-1.458155
H	-5.032414	-1.826255	-0.099649
C	-2.005781	-1.961488	2.834873
H	-2.223156	-3.027798	2.737975
H	-2.115381	-1.678227	3.883302
H	-2.735193	-1.393346	2.252252

ipr-p-3

Symbol	X	Y	Z
C	0.571001	-0.510375	0.634187
C	-0.066486	-1.514238	-0.061384
C	-0.361563	-1.328354	-1.407784
C	-0.023767	-0.132268	-2.037809
C	0.596615	0.854549	-1.310512
N	0.875258	0.639772	-0.003533
H	-0.855119	-2.112803	-1.967361
H	0.851183	-0.574307	1.676029
H	-0.301097	-2.435910	0.453018
H	-0.242003	0.042899	-3.081415
S	1.607864	1.962597	0.944372
O	2.884980	-1.312511	-1.402698
O	0.869879	3.124155	0.539836
O	1.688165	1.488845	2.290233
C	3.352389	2.149495	0.178225
F	3.451303	3.422580	-0.130754
F	3.439652	1.419506	-0.903970
F	4.243253	1.829136	1.070288
S	3.509187	-1.643300	-0.121375
O	3.264220	-0.646322	0.934929
O	3.369509	-3.031896	0.308806
C	5.316809	-1.464044	-0.441874
F	5.693239	-2.263164	-1.436583
F	6.014290	-1.785982	0.643921
F	5.615237	-0.210737	-0.774153
P	-3.969061	-0.467646	-0.846724
O	-3.829855	-0.903015	0.740159
O	-3.877572	1.152521	-0.584784
O	-5.562195	-0.558423	-1.125933
C	-3.430228	-2.250156	1.053306
H	-2.739349	-2.596414	0.273386
C	-2.713909	-2.194630	2.388223
H	-3.407397	-1.871712	3.168043
H	-2.322019	-3.178367	2.653671
H	-1.887712	-1.482026	2.348195
C	-6.550708	0.061197	-0.257641
H	-6.064795	0.298657	0.692386
C	-7.049978	1.331852	-0.921157
H	-7.807045	1.808245	-0.294250
H	-6.225482	2.027625	-1.073401
H	-7.500245	1.093056	-1.887753
C	-2.632617	1.686469	-0.094672
H	-1.888853	0.877758	-0.075080
C	-2.186701	2.774347	-1.053544
H	-1.254100	3.228710	-0.712709
H	-2.042950	2.367134	-2.056683
H	-2.952624	3.551809	-1.103058
H	0.863727	1.818231	-1.724333
C	-4.649880	-3.156116	1.073096
H	-5.366435	-2.789098	1.812387
H	-5.136331	-3.173200	0.095433
H	-4.363789	-4.176050	1.337288
C	-7.652466	-0.957553	-0.039771
H	-8.430162	-0.536482	0.600702
H	-8.101146	-1.230280	-0.997937
H	-7.259942	-1.858550	0.432687
C	-2.839292	2.182535	1.324203
H	-3.599526	2.967344	1.334187
H	-3.168016	1.361069	1.961611

H -1.907589 2.595094 1.718918

ipr-p-4
Symbol X Y Z
C 0.525541 -0.763408 -1.208300
C -0.487215 0.076413 -1.029979
C -1.135251 0.292233 0.317195
C -0.643907 -0.705629 1.336386
C 0.348882 -1.547967 1.085354
N 1.000115 -1.580432 -0.166676
H -0.952331 1.315643 0.674419
H 1.036287 -0.881398 -2.151492
H -0.802377 0.674211 -1.876305
H -1.103447 -0.721102 2.315972
S 2.337312 -2.535585 -0.366691
O 1.546996 1.537258 0.843083
O 2.085929 -3.776116 0.325885
O 2.717570 -2.493577 -1.754120
C 3.717498 -1.720368 0.644517
F 4.229375 -2.658927 1.426042
F 3.227912 -0.758968 1.397355
F 4.656290 -1.271953 -0.153499
S 2.352939 1.793205 -0.351121
O 2.920932 0.603325 -0.994477
O 1.794017 2.783019 -1.274526
C 3.850061 2.631618 0.325897
F 3.514816 3.745673 0.976026
F 4.689944 2.960267 -0.654962
F 4.494029 1.832793 1.174342
P -2.919605 0.107881 0.136480
O -3.465007 0.985702 -1.040043
O -3.341325 -1.350058 -0.210167
O -3.549185 0.451000 1.517742
C -3.163644 2.419087 -1.257479
H -2.086320 2.545661 -1.117071
C -3.542892 2.687128 -2.694837
H -4.613568 2.527034 -2.834052
H -3.306303 3.723051 -2.941161
H -2.990936 2.030977 -3.368221
C -4.955087 0.139362 1.887606
H -5.527246 0.087900 0.957658
C -4.955786 -1.193420 2.603515
H -5.976092 -1.435500 2.905716
H -4.584461 -1.987436 1.956044
H -4.331459 -1.133332 3.497138
C -3.207454 -1.991587 -1.540765
H -2.726799 -1.270413 -2.204994
C -2.333077 -3.208714 -1.343496
H -2.202019 -3.717345 -2.300021
H -1.350892 -2.925356 -0.961336
H -2.804280 -3.898075 -0.640438
H 0.705118 -2.261806 1.815035
C -3.937954 3.245406 -0.253146
H -5.004884 3.026609 -0.337340
H -3.609615 3.051292 0.769011
H -3.779191 4.303557 -0.465491
C -5.423804 1.292280 2.743928
H -6.449651 1.103442 3.063981
H -4.791501 1.379468 3.629401
H -5.399037 2.230839 2.191151
C -4.610399 -2.298020 -2.011681
H -5.102489 -2.970270 -1.306136
H -5.194683 -1.381561 -2.102341
H -4.566366 -2.784064 -2.987678

ipr-p-5
Symbol X Y Z
C 0.940177 1.497593 0.992086
C -0.137429 0.758570 1.230676
C -0.996392 0.194342 0.126375
C -0.419343 0.474028 -1.237098
C 0.650214 1.239382 -1.411532
N 1.349815 1.803019 -0.320605
H -1.167837 -0.883287 0.250133
H 1.574305 1.890423 1.771505
H -0.385263 0.532246 2.260212
H -0.902429 0.036350 -2.100617
S 2.689945 2.733639 -0.604207
O 1.956404 -1.440216 0.086091
O 2.386823 3.587550 -1.727201
O 3.150670 3.255402 0.655542
C 4.019463 1.559484 -1.277151
F 4.450234 2.075859 -2.416977
F 3.503370 0.371321 -1.515959
F 5.021946 1.481697 -0.436104
S 2.848423 -1.155921 1.220373
O 3.372349 0.209015 1.252688
O 2.393999 -1.690130 2.501304

C 4.339005 -2.167230 0.819689
F 4.000551 -3.446605 0.654873
F 5.232671 -2.093134 1.801225
F 4.908703 -1.738230 -0.302442
P -2.622219 0.972253 0.236328
O -3.262088 0.763449 1.650219
O -2.539188 2.518454 0.056970
O -3.478217 0.409114 -0.934040
C -3.386672 -0.528011 2.363316
H -2.434506 -1.053659 2.244506
C -3.611179 -0.156544 3.810169
H -4.547352 0.395712 3.911972
H -3.672351 -1.065920 4.409339
H -2.790653 0.456482 4.183900
C -4.820074 0.918474 -1.310290
H -5.282577 1.307331 -0.398666
C -4.638322 2.013524 -2.339345
H -5.620249 2.376144 -2.648022
H -4.068188 2.848299 -1.932994
H -4.121990 1.615098 -3.214930
C -2.276591 3.503275 1.131102
H -1.861325 2.957303 1.980316
C -1.260500 4.467512 0.564357
H -1.002015 5.209220 1.321572
H -0.353788 3.939122 0.265330
H -1.674656 4.981230 -0.305242
H 1.053484 1.456891 -2.390891
C -4.514313 -1.324665 1.744673
H -5.437420 -0.740542 1.762357
H -4.273418 -1.614589 0.720491
H -4.665152 -2.237711 2.323208
C -5.580534 -0.279352 -1.831574
H -6.579336 0.037006 -2.136383
H -5.065358 -0.696129 -2.699218
H -5.675851 -1.051473 -1.068800
C -3.603142 4.132514 1.493109
H -4.038162 4.616397 0.616218
H -4.296293 3.379548 1.870668
H -3.447863 4.884944 2.268194
Na 0.963725 -3.206063 -0.885697
I -2.126859 -3.383675 -0.986535

ipr-p-6
Symbol X Y Z

C -2.111259 -0.036175 -1.223476
C -0.821252 -0.241314 -1.465102
C 0.158364 -0.510638 -0.350223
C -0.547704 -0.973791 0.898730
C -1.841902 -0.754908 1.091203
N -2.635715 -0.135637 0.089939
H 0.926618 -1.236333 -0.641296
H -2.830029 0.188931 -1.999226
H -0.469156 -0.203596 -2.487425
H 0.029034 -1.476810 1.662774
S -4.260401 0.061572 0.335587
O -4.487586 0.192933 1.749357
O -4.744842 0.998092 -0.642248
C -4.948149 -1.597745 -0.147055
F -6.251798 -1.600958 0.040174
F -4.381088 -2.526257 0.602253
F -4.674045 -1.821448 -1.419189
P 1.035863 1.027378 0.016158
O 1.881422 1.539988 -1.193123
O 0.017191 2.175770 0.304966
O 1.865764 0.756981 1.302453
C 2.912369 0.786356 -1.948600
H 2.623780 -0.267627 -1.933370
C 2.866898 1.346157 -3.350793
H 3.128235 2.406156 -3.340357
H 3.588184 0.813295 -3.972056
H 1.874016 1.226119 -3.784763
C 2.736550 1.743378 1.985887
H 3.170989 2.375094 1.206195
C 1.889583 2.557249 2.941390
H 2.533263 3.260145 3.472929
H 1.118813 3.119029 2.414899
H 1.416539 1.896363 3.670211
C -0.427403 3.203211 -0.666808
H -0.344941 2.761439 -1.661836
C -1.869485 3.494191 -0.323814
H -2.251475 4.260179 -1.000484
H -2.485130 2.600756 -0.427825
H -1.943856 3.862434 0.701140
H -2.367436 -1.043176 1.990291
C 4.237859 0.974482 -1.247462
H 4.478984 2.037126 -1.173934
H 4.215058 0.521080 -0.255687
H 5.019249 0.473636 -1.821961

C 3.804718 0.926667 2.675696
H 4.503129 1.600602 3.174221
H 3.347259 0.277206 3.424622
H 4.353687 0.308937 1.964876
C 0.487923 4.400465 -0.530557
H 0.424336 4.808317 0.480331
H 1.521651 4.130141 -0.749188
H 0.175256 5.172029 -1.236391
I 3.352199 -2.698028 0.016368

ipr-p-7

Symbol	X	Y	Z
C	-3.131174	1.536815	-1.130731
C	-2.054603	2.313710	-1.119159
C	-1.222783	2.486874	0.127345
C	-1.998315	2.112592	1.366569
C	-3.078770	1.345067	1.304259
N	-3.602768	0.927688	0.057753
H	-0.833897	3.507031	0.204166
H	-3.747606	1.377022	-2.003928
H	-1.763478	2.830008	-2.023551
H	-1.662569	2.481051	2.325978
S	-4.679957	-0.321342	-0.008056
O	-5.342581	-0.424481	1.263700
O	-5.376169	-0.261608	-1.264173
C	-3.534886	-1.779445	-0.110213
F	-4.233582	-2.894886	-0.083557
F	-2.700757	-1.744271	0.914472
F	-2.850788	-1.702547	-1.239885
P	0.249903	1.444154	0.028207
O	1.176285	1.979464	-1.108076
O	0.997514	1.502796	1.397491
O	-0.217602	-0.014113	-0.287534
C	2.663760	2.015803	-1.040696
H	2.977493	1.248156	-0.328479
C	3.066407	3.392448	-0.565765
H	2.661168	3.594783	0.427031
H	4.155178	3.447073	-0.512190
H	2.710030	4.152065	-1.264034
C	0.598341	-0.961068	-1.100167
H	1.645077	-0.659086	-0.999264
C	0.411562	-2.324372	-0.480256
H	1.048400	-3.037846	-1.005918
H	0.704020	-2.317340	0.571196
H	-0.625988	-2.647010	-0.565817
C	1.507607	0.326011	2.152035
H	1.862962	-0.398070	1.414207
C	0.371970	-0.235537	2.977265
H	0.007508	0.517916	3.678771
H	-0.453392	-0.564199	2.342337
H	0.734819	-1.095148	3.543787
I	4.387044	-1.472530	0.004710
H	-3.656486	1.050378	2.168835
C	2.674349	0.842127	2.957703
H	3.110790	0.014717	3.519762
H	3.441913	1.252751	2.299955
H	2.341979	1.611061	3.657710
C	0.142663	-0.852603	-2.537498
H	0.738287	-1.532173	-3.150409
H	-0.908093	-1.131456	-2.625037
H	0.277422	0.162092	-2.915923
C	3.149710	1.659768	-2.424860
H	2.821906	0.657684	-2.706862
H	2.779966	2.382284	-3.154982
H	4.240927	1.675399	-2.430991

ipr-p-8

Symbol	X	Y	Z
C	-2.452556	-0.138030	-2.034937
C	-1.562580	0.819889	-2.260473
C	-1.454579	2.016385	-1.347528
C	-2.769213	2.277255	-0.657131
C	-3.642683	1.300228	-0.450356
N	-3.423471	0.011014	-1.008275
H	-1.116713	2.901345	-1.893809
H	-2.556808	-1.029184	-2.637268
H	-0.885144	0.723063	-3.098515
H	-2.992138	3.272808	-0.298340
S	-4.160196	-1.309017	-0.369737
O	-5.346936	-0.882354	0.326133
O	-4.166398	-2.357480	-1.357473
C	-2.969446	-1.867291	0.942107
F	-3.449615	-2.961204	1.511091
F	-2.837126	-0.920022	1.847585
F	-1.806393	-2.136920	0.380303
P	-0.195456	1.642268	-0.083935
O	1.182857	1.429623	-0.862845
O	-0.069475	3.041096	0.676980

C 2.004587 2.492583 -1.418893
H 1.550217 3.453260 -1.160310
C 3.368864 2.367019 -0.769191
H 3.292380 2.471428 0.315242
H 3.793645 1.384936 -0.993753
H 4.041420 3.138345 -1.148623
C 0.627854 3.112919 1.959014
H 1.350920 2.291005 1.999216
C 1.350733 4.442797 1.981665
H 2.059854 4.515792 1.155286
H 0.629327 5.258539 1.899543
H 1.896430 4.551313 2.920530
H -4.585198 1.420820 0.063816
O -0.498218 0.466300 0.761235
C 2.078972 -1.389803 0.298618
H 2.139742 -0.317650 0.461749
C 1.496883 -2.101016 1.502703
H 0.467509 -1.747723 1.613631
H 1.487034 -3.183261 1.355542
H 2.045989 -1.873176 2.416567
I 4.203219 -1.928165 0.068737
C -0.394221 2.959121 3.067811
H -1.131029 3.762982 3.003535
H -0.904270 1.998905 2.982140
H 0.099435 3.012448 4.039907
C 2.043268 2.313238 -2.923448
H 2.449237 1.329505 -3.169462
H 1.045172 2.401144 -3.356435
H 2.680402 3.077266 -3.372321
C 1.344524 -1.692487 -0.991630
H 1.758900 -1.134570 -1.831437
H 1.369760 -2.761121 -1.217160
H 0.303735 -1.383295 -0.852641

ipr-p-9

Symbol X Y Z
C 1.313180 -1.299793 -1.617633
C 0.073829 -1.605697 -1.980472
C -1.004022 -1.930475 -0.979692
C -0.417769 -2.247023 0.369298
C 0.829871 -1.940902 0.696505
N 1.715338 -1.367910 -0.256284
H -1.614100 -2.767542 -1.334970
H 2.102742 -1.053837 -2.313030
H -0.170428 -1.608475 -3.034777
H -1.042992 -2.735703 1.104098
S 3.082151 -0.615016 0.248886
O 3.487675 -1.184054 1.507561
O 3.973151 -0.468070 -0.871704
C 2.476276 1.093145 0.649609
F 3.454657 1.795475 1.186920
F 1.467152 1.008335 1.499446
F 2.067724 1.676371 -0.465266
P -2.171209 -0.530488 -0.873872
O -2.979825 -0.297954 -2.094338
O -3.012851 -0.884716 0.436558
O -1.247933 0.707112 -0.456362
C -1.487810 2.041685 -0.999456
H -2.560583 2.140033 -1.185575
C -1.056569 3.023211 0.069997
H -1.256002 4.042538 -0.265136
H -1.603320 2.846947 0.998261
H 0.011230 2.923414 0.267275
C -3.367839 0.102930 1.448511
H -3.442239 1.080143 0.961488
C -2.282049 0.128878 2.507113
H -2.201867 -0.853865 2.978036
H -1.319415 0.391510 2.065924
H -2.531705 0.864572 3.274565
H 1.272498 -2.156275 1.658268
C -4.723246 -0.302929 1.988486
H -5.047434 0.410010 2.748456
H -5.462880 -0.327062 1.187847
H -4.660276 -1.293783 2.443267
C -0.730687 2.186147 -2.304828
H -0.889927 3.184766 -2.716534
H 0.337503 2.040988 -2.136650
H -1.080990 1.449332 -3.029170

ipr-P

Symbol X Y Z
P 0.074305 -0.007871 -1.078439
O -0.538671 -0.993209 0.096269
O 1.449796 0.397793 -0.274698
O -0.731218 1.381382 -0.848875
C -1.491103 -2.006941 -0.279051
H -1.227229 -2.378433 -1.276682
C -1.356507 -3.128797 0.731826

H	-1.594474	-2.757731	1.731604
H	-2.042004	-3.942852	0.489123
H	-0.336468	-3.516153	0.736024
C	-0.862317	2.003910	0.456714
H	-0.576870	1.269345	1.214258
C	0.068822	3.201074	0.516062
H	-0.023937	3.701536	1.482557
H	1.101528	2.881206	0.379224
H	-0.192205	3.913631	-0.270219
C	2.424105	-0.639965	-0.037243
H	2.158302	-1.522197	-0.634516
C	3.773532	-0.114413	-0.489550
H	4.548672	-0.865708	-0.326392
H	3.748951	0.141472	-1.549822
H	4.030489	0.781335	0.080948
C	-2.886951	-1.406773	-0.312396
H	-3.136657	-1.006261	0.673518
H	-2.945097	-0.594894	-1.040981
H	-3.624084	-2.166676	-0.579415
C	-2.320996	2.383137	0.624861
H	-2.475896	2.863348	1.593277
H	-2.616683	3.081914	-0.161394
H	-2.957821	1.499580	0.565520
C	2.392608	-1.005017	1.436619
H	2.628890	-0.122904	2.037152
H	1.401271	-1.365491	1.711767
H	3.130328	-1.781002	1.652418

ipr-p-TS$_{10/11}$

Symbol	X	Y	Z
C	-1.524091	-1.118429	1.214310
C	-0.279901	-0.668759	1.364317
C	0.607478	-0.294046	0.239962
C	-0.099494	-0.327618	-1.061720
C	-1.340652	-0.782936	-1.221571
N	-2.119984	-1.243446	-0.096383
H	1.593058	-1.381333	0.132964
H	-2.145782	-1.471685	2.022779
H	0.115900	-0.630441	2.373937
H	0.433470	-0.027628	-1.958621
S	-3.746586	-1.142198	-0.217826
O	-4.142496	-1.565878	-1.538628
O	-4.335182	-1.694293	0.978067
C	-4.136679	0.680743	-0.161248
F	-5.431201	0.859008	0.034550
F	-3.781810	1.243064	-1.304996
F	-3.460288	1.243352	0.830874
P	1.555418	1.161422	0.535215
O	1.966258	1.362292	1.953965
O	2.760045	1.041727	-0.512668
O	0.800480	2.452714	-0.089193
C	-0.459578	2.868167	0.496257
H	-0.928540	1.994376	0.964530
C	-1.331029	3.350409	-0.645900
H	-2.309424	3.656406	-0.270238
H	-0.860265	4.205958	-1.136088
H	-1.470162	2.552562	-1.376907
C	3.731120	2.113274	-0.648323
H	3.372910	2.980187	-0.085355
C	3.814993	2.457131	-2.121813
H	4.128681	1.578186	-2.690233
H	2.843390	2.788304	-2.490830
H	4.543448	3.254419	-2.281244
N	2.325752	-2.417010	0.066510
C	1.683684	-3.392757	-0.852829
H	1.523359	-2.874147	-1.797936
H	2.391775	-4.210707	-1.032167
C	3.668975	-2.009556	-0.423174
H	3.978342	-1.158448	0.182971
H	4.365234	-2.836666	-0.239493
C	2.386923	-2.930840	1.459286
H	1.356319	-3.073314	1.785617
H	2.876180	-3.911911	1.445560
C	3.087993	-1.984108	2.424100
H	2.919704	-2.341171	3.441073
H	4.165172	-1.949423	2.259245
H	2.689424	-0.968418	2.350866
C	0.353742	-3.938217	-0.353707
H	-0.121102	-4.485376	-1.169154
H	0.474038	-4.626154	0.483229
H	-0.318257	-3.132127	-0.053363
C	3.688043	-1.602739	-1.889170
H	4.651630	-1.137963	-2.106072
H	3.571846	-2.454197	-2.559739
H	2.911222	-0.864743	-2.095707
H	-1.825307	-0.897568	-2.180091
C	5.045363	1.626440	-0.068262
H	5.402838	0.759493	-0.629690

H	5.799260	2.413699	-0.129794
H	4.918014	1.342671	0.977971
C	-0.197582	3.936183	1.542641
H	0.453002	3.548104	2.327133
H	0.284302	4.798704	1.075788
H	-1.138920	4.262023	1.990035

ipr-p-TS$_{12/13}$

Symbol	X	Y	Z
C	-1.522970	-2.334281	0.240657
C	-0.561130	-1.415000	0.526342
C	-0.268875	-0.374488	-0.399130
C	-0.811559	-0.516984	-1.704992
C	-1.760308	-1.462639	-1.946923
N	-2.381901	-2.129890	-0.864802
H	2.911582	-1.006222	0.522878
H	-1.737315	-3.193216	0.861047
H	0.043154	-1.530787	1.421272
H	-0.403340	0.062759	-2.526126
S	-3.963159	-1.281237	-0.351925
O	-4.758397	-1.087452	-1.563646
O	-4.515395	-2.043247	0.767576
C	-3.835050	0.462836	0.349147
F	-5.046401	0.936109	0.622804
F	-3.254563	1.270817	-0.530191
F	-3.121332	0.457345	1.469729
P	0.937812	0.826255	-0.011206
O	1.987337	0.306583	0.935700
O	1.521895	1.332940	-1.406197
O	0.273480	2.180476	0.544100
C	-0.374582	2.192158	1.844730
H	-0.795925	1.196674	2.023735
C	-1.494193	3.208836	1.757445
H	-2.047528	3.234282	2.698073
H	-1.079547	4.201687	1.568099
H	-2.181366	2.953549	0.951498
C	1.737907	2.725892	-1.774823
H	0.794501	3.250060	-1.611159
C	2.084263	2.706403	-3.248752
H	3.011245	2.150478	-3.407645
H	1.286239	2.235799	-3.824277
H	2.222427	3.727044	-3.609584
N	3.669606	-1.716898	0.349244
C	3.429366	-2.359178	-0.985562
H	3.377828	-1.546910	-1.708168
H	4.309534	-2.966254	-1.208683
C	4.946414	-0.929271	0.365891
H	4.957257	-0.392125	1.311962
H	5.769777	-1.647019	0.360467
C	3.604367	-2.702754	1.475450
H	2.622332	-3.168191	1.419133
H	4.362223	-3.464801	1.280605
C	3.782508	-2.048424	2.835944
H	3.499034	-2.768338	3.603594
H	4.812530	-1.748089	3.023932
H	3.131246	-1.176226	2.928176
C	2.142873	-3.166102	-1.038212
H	1.912733	-3.381825	-2.081723
H	2.219348	-4.114151	-0.507115
H	1.318321	-2.582864	-0.623268
C	5.035006	0.058252	-0.786344
H	5.825085	0.776971	-0.568185
H	5.272778	-0.425985	-1.732878
H	4.094737	0.604785	-0.891322
H	-2.144466	-1.690672	-2.931263
C	2.828735	3.347318	-0.920694
H	2.571059	3.306696	0.138633
H	3.779244	2.834241	-1.080745
H	2.953008	4.394870	-1.202813
C	0.651632	2.532113	2.910830
H	1.073868	3.520303	2.712008
H	0.174263	2.548906	3.892679
H	1.456430	1.797070	2.917571

ipr-p-TS$_{3/4}$

Symbol	X	Y	Z
C	0.588962	0.333470	1.495138
C	-0.156444	-0.785779	1.625735
C	-0.569646	-1.510124	0.465052
C	0.053095	-1.164278	-0.772287
C	0.793954	-0.034596	-0.864866
N	0.978639	0.759678	0.243177
H	-0.939142	-2.518797	0.592639
H	0.935784	0.925960	2.329981
H	-0.439949	-1.116107	2.615145
H	-0.060438	-1.790890	-1.645605
S	2.008996	2.133049	0.121565
O	3.071180	-2.772179	0.521412

O	2.544653	2.410682	1.420145
O	2.767090	2.022998	-1.087739
C	0.673962	3.396890	-0.186009
F	1.223025	4.587805	-0.236065
F	-0.205124	3.337581	0.795837
F	0.082420	3.114839	-1.332952
S	3.650279	-1.551065	-0.038156
O	3.429305	-1.353046	-1.471434
O	3.459170	-0.347755	0.781380
C	5.468028	-1.839858	0.075222
F	5.834728	-2.038809	1.339657
F	6.139263	-0.790882	-0.394392
F	5.817470	-2.910981	-0.634766
P	-2.941283	-0.933194	-0.013366
O	-3.945558	-0.025488	0.872299
O	-3.009552	-0.193295	-1.446782
O	-3.730779	-2.288318	-0.305492
C	-3.730216	0.136099	2.306193
H	-3.087199	-0.679660	2.654139
C	-3.051000	1.471819	2.535878
H	-3.696523	2.282688	2.191302
H	-2.849362	1.611799	3.599376
H	-2.103013	1.526516	1.996013
C	-5.186670	-2.417030	-0.414078
H	-5.610312	-1.938359	0.471710
C	-5.692715	-1.732358	-1.669537
H	-6.766049	-1.908791	-1.763614
H	-5.517625	-0.656972	-1.635144
H	-5.191051	-2.142875	-2.548584
C	-2.442702	1.135573	-1.614283
H	-1.661750	1.275427	-0.855317
C	-1.835317	1.168883	-3.002329
H	-1.382147	2.142370	-3.193969
H	-1.075616	0.394997	-3.117000
H	-2.619262	0.999416	-3.743421
H	1.309381	0.272719	-1.764369
C	-5.462636	-3.904969	-0.393278
H	-6.539853	-4.078519	-0.413771
H	-5.014617	-4.381892	-1.267555
H	-5.051085	-4.359138	0.508468
C	-5.088449	0.034609	2.967793
H	-5.748060	0.812232	2.576905
H	-5.539513	-0.940353	2.780095
H	-4.987110	0.171136	4.045707
C	-3.525934	2.180813	-1.422224
H	-4.309674	2.039466	-2.170041
H	-3.966720	2.109498	-0.428634
H	-3.096454	3.176803	-1.549664

ipr-p-$TS_{7/8}$

Symbol	X	Y	Z
C	-2.587422	0.204767	-1.990067
C	-1.637323	1.117104	-2.155159
C	-1.367703	2.170404	-1.109590
C	-2.578925	2.390411	-0.238759
C	-3.516385	1.461495	-0.106635
N	-3.469300	0.271544	-0.880197
H	-1.047737	3.109955	-1.570752
H	-2.804053	-0.580449	-2.700394
H	-1.031952	1.088296	-3.051546
H	-2.674253	3.320559	0.303947
S	-4.327676	-1.050842	-0.414652
O	-5.433613	-0.619984	0.398821
O	-4.474469	-1.928591	-1.545126
C	-3.150569	-1.900426	0.743505
F	-3.738561	-2.962329	1.259664
F	-2.804103	-1.066688	1.705756
F	-2.075493	-2.272107	0.066571
P	0.014035	1.604002	-0.072831
O	1.338658	1.603490	-0.953307
O	0.147415	2.760299	1.002455
C	2.055296	2.809610	-1.367290
H	1.567103	3.671837	-0.903878
C	3.470535	2.669522	-0.845982
H	3.475912	2.573712	0.241547
H	3.937155	1.781015	-1.277084
H	4.057892	3.546645	-1.122437
C	0.904544	2.550363	2.242491
H	1.676423	1.799912	2.042127
C	1.543267	3.880174	2.576841
H	2.196283	4.213116	1.768576
H	0.769545	4.633349	2.738774
H	2.135500	3.784212	3.488215
H	-4.391382	1.566632	0.518418
O	-0.164773	0.226733	0.489348
C	1.410820	-1.217909	0.194666
H	2.056684	-0.361865	0.282815
C	1.057750	-2.010027	1.395246

H	-0.031175	-2.028992	1.472145
H	1.394907	-3.039382	1.263946
H	1.487453	-1.590905	2.301904
I	4.307282	-2.074458	-0.047830
C	-0.053194	2.046458	3.302652
H	-0.841357	2.784264	3.467828
H	-0.504392	1.102482	2.993807
H	0.483981	1.891041	4.239933
C	1.974858	2.897505	-2.877211
H	2.418496	2.005825	-3.324764
H	0.940199	2.984405	-3.213667
H	2.523453	3.774502	-3.224559
C	0.969515	-1.656329	-1.150772
H	1.352948	-1.003251	-1.931744
H	1.294510	-2.683113	-1.325690
H	-0.123408	-1.649069	-1.163279

23

Symbol	X	Y	Z
C	-1.845293	2.833054	-0.307071
C	-2.801085	3.742303	0.131146
C	-4.067120	3.264582	0.445461
C	-4.326155	1.909010	0.300387
C	-3.306368	1.070619	-0.155186
N	-2.081940	1.528912	-0.449653
H	-4.843029	3.937278	0.790344
H	-0.838517	3.161612	-0.543404
H	-2.552583	4.791133	0.225281
H	-5.305960	1.501308	0.515518
C	-3.533415	-0.384646	-0.342466
C	-3.005639	-1.026530	-1.465217
C	-4.253166	-1.126364	0.596284
C	-3.197156	-2.390327	-1.646625
H	-2.437853	-0.448263	-2.183162
C	-4.435208	-2.493650	0.418256
H	-4.648620	-0.637410	1.479768
C	-3.909096	-3.127445	-0.703607
H	-2.788921	-2.880037	-2.522743
H	-4.982443	-3.064576	1.158816
H	-4.052330	-4.192424	-0.841987
S	0.598634	0.074697	-0.401445
O	2.046934	-0.783427	-0.299353
O	0.865069	1.426862	-0.029618
O	0.086727	-0.361607	-1.655982
C	-0.258943	-0.820419	1.031084
F	0.671468	-1.224815	1.874684
F	-0.906128	-1.847502	0.544760
F	-1.065345	0.011057	1.636960
S	3.356717	-0.476240	0.628802
O	3.017934	0.434498	1.678264
O	3.975695	-1.743888	0.848866
C	4.338117	0.451427	-0.654616
F	4.716314	-0.385456	-1.592425
F	5.383987	0.978238	-0.056786
F	3.577760	1.397866	-1.161970

24

Symbol	X	Y	Z
C	-1.906419	2.788657	-0.157046
C	-3.090778	3.486553	-0.206140
C	-4.277625	2.778374	-0.337516
C	-4.242016	1.392884	-0.422353
C	-3.038098	0.707366	-0.384960
N	-1.895258	1.441311	-0.249655
H	-5.226388	3.298049	-0.374007
H	-0.944676	3.269329	-0.046356
H	-3.069146	4.564183	-0.136326
H	-5.149319	0.813417	-0.524739
C	-3.037973	-0.780713	-0.445827
C	-2.719604	-1.452496	-1.627688
C	-3.505366	-1.489648	0.662419
C	-2.827076	-2.835138	-1.680007
H	-2.386787	-0.895056	-2.493588
C	-3.600427	-2.875110	0.604460
H	-3.782724	-0.960930	1.566472
C	-3.257142	-3.547054	-0.563343
H	-2.576146	-3.356221	-2.595214
H	-3.947937	-3.424872	1.469938
H	-3.333366	-4.626534	-0.607798
S	-0.220614	0.667775	-0.200883
O	1.995027	-0.757298	0.235598
O	0.644955	1.796756	-0.064906
O	-0.224576	-0.245620	-1.295331
C	-0.389203	-0.197083	1.502773
F	0.593622	0.194806	2.260896
F	-0.395542	-1.486727	1.310587
F	-1.531175	0.190446	2.041979
S	3.360823	-0.361969	0.619476

O 3.451540 0.861306 1.409303
O 4.199128 -1.470578 1.067688
C 4.096646 0.111066 -1.002104
F 4.094718 -0.924921 -1.838275
F 5.354529 0.518910 -0.844698
F 3.404052 1.100161 -1.561944

25

Symbol	X	Y	Z
C	-0.927799	-1.543050	0.392717
C	-0.251877	-2.034963	-0.690025
C	-0.009982	-1.191901	-1.773101
C	-0.534374	0.088692	-1.766914
C	-1.285466	0.554685	-0.695615
N	-1.401497	-0.265857	0.394218
H	0.550284	-1.545107	-2.628343
H	-1.113642	-2.124364	1.284637
H	0.092992	-3.058452	-0.671170
H	-0.426570	0.741141	-2.622648
C	-1.959730	1.866839	-0.808751
C	-3.353569	1.944324	-0.783394
C	-1.193784	2.995131	-1.107536
C	-3.973372	3.160518	-1.031691
H	-3.934869	1.056144	-0.579265
C	-1.821919	4.212782	-1.335256
H	-0.113085	2.918452	-1.157034
C	-3.210819	4.294875	-1.297995
H	-5.054607	3.219491	-1.020243
H	-1.226996	5.091731	-1.549131
H	-3.700267	5.242784	-1.485543
S	-2.039835	0.269703	1.989855
O	-4.172462	-3.332652	-0.723765
O	-3.070777	1.241785	1.841773
O	-2.126986	-0.908321	2.796187
C	-0.481493	1.159146	2.525803
F	-0.655024	1.544060	3.765746
F	-0.285859	2.199749	1.740760
F	0.530324	0.323581	2.432906
S	-4.150840	-1.873080	-0.715422
O	-4.028720	-1.259970	0.615030
O	-3.304659	-1.247317	-1.734205
C	-5.860193	-1.403925	-1.226433
F	-6.123462	-1.859663	-2.447577
F	-5.997557	-0.078022	-1.236191
F	-6.756054	-1.910060	-0.384531
P	3.327311	-0.624163	-0.885157
C	5.066974	-1.065951	-1.295102
C	6.078199	-1.198661	-0.339600
C	5.379759	-1.240899	-2.645971
C	7.379368	-1.493845	-0.731915
H	5.850318	-1.073630	0.713076
C	6.684451	-1.526139	-3.038704
H	4.599169	-1.151554	-3.394484
C	7.685194	-1.654311	-2.081170
H	8.156069	-1.597678	0.016482
H	6.915624	-1.655603	-4.089221
H	8.700032	-1.883278	-2.383745
C	3.454045	1.201566	-0.669901
C	2.353700	1.861303	-0.108280
C	4.553997	1.957163	-1.076734
C	2.362540	3.238727	0.070237
H	1.497577	1.278471	0.218671
C	4.554442	3.342204	-0.917321
H	5.418309	1.469704	-1.513036
C	3.465674	3.984968	-0.340798
H	1.513893	3.731576	0.531346
H	5.415643	3.916406	-1.237856
H	3.474001	5.059937	-0.208201
C	3.185717	-1.175295	0.868033
C	2.697197	-2.467826	1.084339
C	3.537013	-0.392744	1.972017
C	2.557800	-2.968868	2.374819
H	2.430893	-3.089428	0.235462
C	3.395341	-0.892663	3.262568
H	3.918251	0.611801	1.826419
C	2.901991	-2.178091	3.466848
H	2.176468	-3.971650	2.526082
H	3.668647	-0.275197	4.110015
H	2.786881	-2.561952	4.473393

26

Symbol	X	Y	Z
C	0.511332	0.152191	-1.778732
C	-0.427954	-0.760738	-1.569070
C	-0.923210	-1.096163	-0.198460
C	-0.039933	-0.489115	0.847036
C	0.888075	0.436899	0.607193
N	1.136407	0.876667	-0.732356

H	-0.995644	-2.180935	-0.074755
H	0.870434	0.374336	-2.771964
H	-0.834663	-1.282505	-2.424954
H	-0.187311	-0.773010	1.881761
C	1.606301	1.054721	1.754095
C	2.561370	0.318444	2.451700
C	1.250528	2.331418	2.184323
C	3.174757	0.871621	3.569959
H	2.829045	-0.667319	2.089616
C	1.870249	2.882401	3.300669
H	0.483899	2.883288	1.652192
C	2.835000	2.154593	3.991647
H	3.921624	0.302826	4.110710
H	1.592357	3.874326	3.636362
H	3.316633	2.584135	4.861954
S	2.547625	1.667204	-1.187705
O	2.681918	-2.902949	-2.194519
O	3.459346	1.836993	-0.093419
O	2.969330	1.195105	-2.482497
C	1.827283	3.340933	-1.539876
F	2.769515	4.104337	-2.062664
F	1.375099	3.893661	-0.429728
F	0.832915	3.210361	-2.405809
S	2.584434	-2.556614	-0.776346
O	3.032641	-1.201866	-0.439645
O	1.356635	-3.000589	-0.113097
C	3.876299	-3.605597	0.018406
F	3.625225	-4.899023	-0.179136
F	3.912316	-3.386519	1.333280
F	5.080588	-3.334611	-0.480519
P	-2.638762	-0.400241	0.032359
C	-3.429174	-1.154624	1.465917
C	-4.733613	-0.758351	1.783012
C	-2.779750	-2.127272	2.227037
C	-5.376976	-1.329411	2.871037
H	-5.243986	-0.010633	1.184859
C	-3.435850	-2.695436	3.314423
H	-1.776853	-2.456143	1.984946
C	-4.727190	-2.295811	3.636387
H	-6.385244	-1.023632	3.119557
H	-2.933849	-3.450592	3.905556
H	-5.233386	-2.740736	4.484184
C	-2.461250	1.373728	0.289883
C	-2.060242	2.181642	-0.781802
C	-2.600608	1.913690	1.570236
C	-1.808719	3.529733	-0.563311
H	-1.952063	1.767324	-1.777745
C	-2.355574	3.267214	1.773681
H	-2.895678	1.287893	2.404072
C	-1.958874	4.071753	0.710900
H	-1.498659	4.156740	-1.389453
H	-2.468227	3.688178	2.764765
H	-1.764321	5.124834	0.874046
C	-3.640694	-0.721543	-1.430438
C	-3.583482	-1.997379	-2.001699
C	-4.499664	0.250402	-1.946628
C	-4.377870	-2.290767	-3.100907
H	-2.926855	-2.759439	-1.595088
C	-5.294530	-0.057032	-3.045825
H	-4.552564	1.236174	-1.499880
C	-5.230880	-1.320612	-3.622217
H	-4.331454	-3.274977	-3.549144
H	-5.961427	0.693904	-3.449902
H	-5.848995	-1.553126	-4.480617

27

Symbol	X	Y	Z
C	-0.953882	-0.365154	-1.757268
C	0.298679	0.011115	-1.529327
C	0.902062	0.010629	-0.158782
C	-0.086219	-0.443591	0.870021
C	-1.333254	-0.842063	0.611259
N	-1.813170	-0.848574	-0.735879
H	1.263657	1.023734	0.084924
H	-1.406974	-0.300657	-2.735227
H	0.870252	0.405166	-2.359911
H	0.183753	-0.353562	1.914152
C	-2.240000	-1.167548	1.745897
C	-3.300510	-0.317785	2.069208
C	-1.958710	-2.270372	2.548876
C	-4.073885	-0.581662	3.190225
H	-3.503198	0.536797	1.436110
C	-2.742381	-2.533898	3.669863
H	-1.129689	-2.920365	2.290943
C	-3.799601	-1.690929	3.989970
H	-4.893626	0.079571	3.444320
H	-2.523615	-3.395105	4.289383
H	-4.408816	-1.893367	4.862813

S	-3.166890	-1.675777	-1.246892
O	-3.914940	1.352892	-0.947442
O	-4.177993	-1.734391	-0.232663
O	-3.443395	-1.282246	-2.603063
C	-2.503639	-3.406297	-1.372785
F	-3.481304	-4.231979	-1.686643
F	-1.969864	-3.762877	-0.215511
F	-1.569174	-3.446067	-2.313751
S	-2.842441	2.341088	-0.805731
O	-1.899262	2.066583	0.280795
O	-2.240126	2.786357	-2.063037
C	-3.721060	3.844218	-0.196934
F	-4.641975	4.246670	-1.070517
F	-2.859672	4.843563	-0.009399
F	-4.327796	3.594377	0.962490
P	2.493082	-0.947080	-0.009041
C	3.170793	-0.482328	1.597043
C	4.112744	0.543113	1.694262
C	2.627360	-1.055441	2.753444
C	4.491588	1.012082	2.946999
H	4.556620	0.971376	0.805638
C	3.013662	-0.580205	3.999640
H	1.909492	-1.865646	2.685183
C	3.936252	0.458868	4.095892
H	5.223268	1.807060	3.021168
H	2.593083	-1.021508	4.894203
H	4.230340	0.829346	5.070166
C	2.245506	-2.725855	-0.051696
C	1.230081	-3.268820	-0.843353
C	3.120809	-3.558141	0.653042
C	1.088700	-4.648719	-0.920445
H	0.568573	-2.622792	-1.407003
C	2.971107	-4.936288	0.565590
H	3.911117	-3.136681	1.263419
C	1.956572	-5.478862	-0.217473
H	0.305435	-5.075255	-1.533515
H	3.646664	-5.584150	1.109336
H	1.842809	-6.553936	-0.282238
C	3.567647	-0.427386	-1.354955
C	3.694219	0.941460	-1.621157
C	4.235049	-1.372532	-2.135942
C	4.501890	1.357107	-2.671961
H	3.173576	1.677728	-1.010661
C	5.040804	-0.941119	-3.184006
H	4.126334	-2.431796	-1.934688
C	5.173620	0.417565	-3.450624
H	4.603699	2.415078	-2.883501
H	5.560596	-1.669232	-3.793594
H	5.800270	0.746756	-4.270352
N	2.136316	3.415530	0.215011
C	0.907913	3.666300	-0.561866
H	0.138136	2.969764	-0.225790
H	0.527700	4.680255	-0.339435
C	1.851719	3.604105	1.645412
H	2.800170	3.627659	2.183314
H	1.374305	4.588277	1.795500
C	3.199933	4.339218	-0.205038
H	3.255926	4.314907	-1.294040
H	2.931743	5.373652	0.073924
C	4.585371	3.999069	0.333894
H	5.307236	4.723409	-0.048819
H	4.641333	4.026700	1.422496
H	4.900216	3.009444	-0.003545
C	1.060673	3.512205	-2.073130
H	0.065870	3.426192	-2.512264
H	1.566792	4.359975	-2.537116
H	1.619922	2.607652	-2.324532
C	0.990071	2.511447	2.269241
H	0.755056	2.783071	3.300739
H	0.046347	2.357537	1.739881
H	1.540373	1.567711	2.297331

28

Symbol	X	Y	Z
C	-0.654778	1.044832	0.682333
C	0.583745	0.656391	1.045819
C	1.478033	0.068229	0.078095
C	0.892298	-0.376806	-1.145761
C	-0.369969	-0.005603	-1.500556
N	-0.959936	1.069980	-0.728392
H	-2.314631	-1.614164	0.183037
H	-1.454288	1.327168	1.350299
H	0.851638	0.672986	2.098802
H	1.399157	-1.139964	-1.725489
C	-1.233660	-0.684815	-2.477107
C	-2.626260	-0.508736	-2.445500
C	-0.703800	-1.555116	-3.445006
C	-3.453175	-1.210289	-3.315362

H	-3.056531	0.161727	-1.712720
C	-1.535306	-2.252312	-4.311596
H	0.369486	-1.688838	-3.526853
C	-2.918527	-2.093665	-4.248484
H	-4.526356	-1.064834	-3.257400
H	-1.098158	-2.919699	-5.045222
H	-3.565082	-2.639291	-4.924800
S	-1.086323	2.553707	-1.427789
O	-5.239795	1.402545	-0.250888
O	-1.321520	2.391264	-2.845295
O	-1.947070	3.376166	-0.605749
C	0.567289	3.420760	-1.341705
F	0.514704	4.515288	-2.089724
F	1.529177	2.634345	-1.800003
F	0.844793	3.771448	-0.092444
S	-4.820202	0.436554	0.760179
O	-5.800151	-0.597392	1.094417
O	-3.445716	-0.057694	0.604556
C	-4.696137	1.428952	2.310475
F	-3.773606	2.381188	2.187982
F	-4.352687	0.645898	3.332057
F	-5.859844	2.006176	2.597302
P	3.145974	-0.250106	0.462390
C	3.797152	-1.529155	-0.640146
C	4.002935	-2.833124	-0.189133
C	4.037043	-1.199500	-1.978803
C	4.438101	-3.808512	-1.081126
H	3.832183	-3.090157	0.849838
C	4.466644	-2.180490	-2.863048
H	3.890281	-0.182428	-2.327553
C	4.665111	-3.484049	-2.414145
H	4.601817	-4.819882	-0.730727
H	4.649469	-1.926383	-3.899561
H	5.003100	-4.246426	-3.105358
C	4.235720	1.191820	0.275656
C	3.701058	2.454290	0.534838
C	5.582891	1.053069	-0.065872
C	4.512006	3.579408	0.448968
H	2.653786	2.556194	0.792841
C	6.388546	2.183390	-0.151177
H	6.001678	0.073025	-0.265728
C	5.853937	3.443158	0.104744
H	4.094477	4.559484	0.644564
H	7.432450	2.079809	-0.419904
H	6.484663	4.320932	0.033112
C	3.315131	-0.786173	2.188492
C	2.399984	-1.711725	2.700600
C	4.352313	-0.311754	2.993535
C	2.526114	-2.162367	4.007607
H	1.590851	-2.075670	2.078170
C	4.471868	-0.766425	4.302970
H	5.059388	0.413313	2.608910
C	3.562573	-1.688691	4.808567
H	1.814684	-2.877883	4.400827
H	5.274337	-0.394601	4.927635
H	3.657554	-2.037231	5.829718
N	-2.200127	-2.589042	0.507700
C	-2.907946	-2.641614	1.838369
H	-3.917428	-2.277066	1.662713
H	-2.944283	-3.696507	2.119768
C	-2.862481	-3.472033	-0.513621
H	-2.356261	-3.278478	-1.460032
H	-2.669361	-4.502801	-0.211972
C	-0.725554	-2.879708	0.686765
H	-0.281201	-1.926087	0.975210
H	-0.652537	-3.573099	1.525939
C	-0.010012	-3.467210	-0.516894
H	1.058453	-3.476960	-0.286539
H	-0.319528	-4.493361	-0.714779
H	-0.153208	-2.875274	-1.417215
C	-2.227742	-1.789538	2.897463
H	-2.888444	-1.737001	3.763283
H	-1.274537	-2.203437	3.226536
H	-2.075607	-0.774843	2.527576
C	-4.348843	-3.193967	-0.652242
H	-4.701193	-3.683634	-1.560855
H	-4.924170	-3.577234	0.189915
H	-4.538047	-2.124310	-0.746181

29

Symbol	X	Y	Z
C	-0.557289	-0.976834	-2.471370
C	0.688316	-1.202263	-2.003999
C	1.065527	-0.738576	-0.696949
C	0.244950	0.272812	-0.110945
C	-1.010250	0.505919	-0.585739
N	-1.527314	-0.475104	-1.525624
H	-5.153037	-0.475266	0.262841

H	-0.888733	-1.114654	-3.489542
H	1.433902	-1.622680	-2.673988
H	0.680473	0.958181	0.609984
C	-1.843804	1.686671	-0.332318
C	-2.947792	1.978898	-1.147036
C	-1.554454	2.567103	0.723589
C	-3.718956	3.115641	-0.927563
H	-3.185025	1.319861	-1.973919
C	-2.333157	3.694466	0.942445
H	-0.698238	2.383366	1.361407
C	-3.422350	3.980811	0.120940
H	-4.552229	3.332169	-1.588333
H	-2.083468	4.359458	1.761489
H	-4.025730	4.863839	0.293612
S	-2.603095	-1.588315	-0.954099
O	3.458203	4.382754	0.880449
O	-3.418392	-0.955656	0.076156
O	-3.230762	-2.252829	-2.077554
C	-1.758475	-2.979946	-0.025008
F	-0.938572	-2.501523	0.893082
F	-1.091570	-3.752846	-0.866252
F	-2.698044	-3.704217	0.571211
S	2.489889	3.398801	0.390262
O	3.014023	2.459342	-0.604425
O	1.630779	2.817154	1.421834
C	1.318002	4.415236	-0.611457
F	0.613047	5.236734	0.166514
F	0.469097	3.638354	-1.279439
F	1.981937	5.157703	-1.499968
P	2.586962	-1.213441	0.007104
C	3.938376	-1.099673	-1.198186
C	4.983579	-2.026265	-1.207210
C	3.926636	-0.036485	-2.104818
C	6.020737	-1.882873	-2.122140
H	4.987848	-2.857541	-0.511666
C	4.967787	0.097510	-3.015787
H	3.120892	0.686748	-2.072995
C	6.012015	-0.823229	-3.024473
H	6.831269	-2.600988	-2.132697
H	4.963226	0.921325	-3.718902
H	6.820145	-0.716442	-3.738201
C	2.967330	-0.220668	1.464721
C	2.102715	-0.289588	2.561347
C	4.110735	0.572557	1.514063

C	2.383555	0.446949	3.703142
H	1.218499	-0.918069	2.523108
C	4.387559	1.302954	2.665129
H	4.770309	0.633298	0.657185
C	3.526225	1.241315	3.753941
H	1.713626	0.399594	4.552712
H	5.273360	1.924875	2.705198
H	3.744286	1.814540	4.647141
C	2.590015	-2.944972	0.579158
C	1.895444	-3.898684	-0.166748
C	3.301854	-3.330842	1.717056
C	1.906224	-5.230909	0.227365
H	1.334055	-3.601803	-1.044435
C	3.311726	-4.667017	2.105096
H	3.843245	-2.594887	2.300674
C	2.614373	-5.615008	1.363023
H	1.359101	-5.966464	-0.349569
H	3.861654	-4.964632	2.989374
H	2.619831	-6.653489	1.671343
N	-6.132350	-0.212817	0.462584
C	-6.900122	-0.302850	-0.827837
H	-7.195625	-1.342734	-0.955194
H	-7.801782	0.295772	-0.690308
C	-6.658441	-1.188000	1.480724
H	-6.339501	-0.836168	2.459789
H	-7.746578	-1.126232	1.431471
C	-6.109767	1.193534	0.997738
H	-6.036580	1.861052	0.140129
H	-7.074513	1.353316	1.482077
C	-4.936291	1.418819	1.932456
H	-4.955627	2.454951	2.270507
H	-4.974495	0.775858	2.812686
H	-3.992959	1.256384	1.406224
C	-6.071839	0.161041	-2.011251
H	-6.681834	0.096417	-2.912266
H	-5.741679	1.194608	-1.903053
H	-5.198105	-0.479498	-2.147149
C	-6.144338	-2.594169	1.229110
H	-6.574237	-3.262421	1.975101
H	-6.425003	-2.968810	0.243775
H	-5.058205	-2.627837	1.321254

2-Ph-Py

Symbol	X	Y	Z

C	-2.697932	-1.181834	-0.286285
C	-3.501955	-0.078661	-0.022556
C	-2.877306	1.125494	0.276475
C	-1.491096	1.177561	0.291106
C	-0.763880	0.019963	-0.004748
N	-1.366487	-1.144308	-0.280477
H	-3.459805	2.009922	0.504769
H	-3.146607	-2.143356	-0.515947
H	-4.580001	-0.168439	-0.045314
H	-0.980644	2.096203	0.548812
C	0.724274	0.017924	-0.006087
C	1.416558	-1.168123	0.256318
C	1.453352	1.180771	-0.271077
C	2.805471	-1.186901	0.269347
H	0.849938	-2.069810	0.451091
C	2.843782	1.159972	-0.262915
H	0.938226	2.103832	-0.509742
C	3.524199	-0.022308	0.011344
H	3.328805	-2.111282	0.483117
H	3.395238	2.067251	-0.478505
H	4.607463	-0.037320	0.019527

30

Symbol	X	Y	Z
C	0.143113	0.518234	-3.251499
C	0.921915	-0.427672	-2.593661
C	0.997788	-0.337205	-1.206527
C	0.315892	0.673702	-0.540096
C	-0.443571	1.579392	-1.293394
N	-0.515154	1.488720	-2.628585
H	-4.316257	0.187680	-0.148075
H	0.054854	0.490660	-4.332520
H	1.448874	-1.190605	-3.154047
H	0.394625	0.775355	0.533629
C	-1.211246	2.687901	-0.660059
C	-2.116601	3.425212	-1.430028
C	-1.020584	3.044432	0.678977
C	-2.815429	4.491911	-0.876752
H	-2.252215	3.160831	-2.470454
C	-1.715702	4.115448	1.227817
H	-0.292985	2.524516	1.288909
C	-2.615090	4.843672	0.454617
H	-3.513105	5.051936	-1.488933
H	-1.544425	4.387817	2.262523
H	-3.154971	5.678457	0.885994
S	-2.137355	-1.312288	0.286090
O	4.241989	3.015894	1.634462
O	-2.964592	-0.131695	0.773065
O	-2.748103	-1.911085	-0.949073
C	-2.593833	-2.631992	1.594617
F	-1.450162	-3.173668	2.043960
F	-3.341258	-3.616671	1.100807
F	-3.229153	-2.116345	2.649069
S	3.058991	2.597915	0.881913
O	3.332705	1.735409	-0.272898
O	1.911532	2.200835	1.697394
C	2.489534	4.159050	0.077194
F	1.993732	5.004016	0.980154
F	1.541112	3.907848	-0.823000
F	3.500629	4.762927	-0.546729
P	1.966710	-1.580769	-0.302389
C	3.439810	-1.937365	-1.281716
C	3.786699	-3.257559	-1.575709
C	4.218691	-0.870460	-1.745753
C	4.925980	-3.509986	-2.332183
H	3.175106	-4.081762	-1.229438
C	5.354514	-1.139281	-2.497848
H	3.942150	0.150190	-1.503559
C	5.706978	-2.454808	-2.790301
H	5.197402	-4.531751	-2.565589
H	5.963860	-0.319694	-2.857511
H	6.592134	-2.656926	-3.381167
C	2.381983	-1.016989	1.353273
C	1.365504	-0.990841	2.313299
C	3.679876	-0.624125	1.675257
C	1.652754	-0.547925	3.596117
H	0.363872	-1.326336	2.064088
C	3.956346	-0.189327	2.965693
H	4.465050	-0.648655	0.929904
C	2.945889	-0.147384	3.919657
H	0.868886	-0.521016	4.342463
H	4.961373	0.121401	3.221214
H	3.167091	0.196204	4.922951
C	1.009242	-3.100200	-0.118559
C	-0.046692	-3.397688	-0.978583
C	1.396062	-4.006429	0.875680
C	-0.728966	-4.600767	-0.830349
H	-0.370683	-2.690226	-1.731678

C 0.721185 -5.212344 1.000128
H 2.211777 -3.769620 1.549954
C -0.342366 -5.506566 0.150249
H -1.569042 -4.818270 -1.477368
H 1.017071 -5.915192 1.768623
H -0.877906 -6.441474 0.261924
N -5.195666 0.390843 -0.697283
C -4.962473 -0.085360 -2.100016
H -4.837164 -1.163895 -2.028582
H -5.868872 0.140381 -2.665102
C -6.306945 -0.394000 -0.059140
H -6.740847 0.226133 0.723452
H -7.064859 -0.542644 -0.829961
C -5.440199 1.865934 -0.609542
H -4.807792 2.349783 -1.351231
H -6.485075 2.034563 -0.878105
C -5.093059 2.392032 0.772639
H -5.291323 3.463510 0.806069
H -5.679844 1.913248 1.558477
H -4.031308 2.233747 0.974984
C -3.723466 0.532011 -2.725055
H -3.410238 -0.082086 -3.570020
H -3.894796 1.544099 -3.091066
H -2.903132 0.546306 -2.007013
C -5.807721 -1.706203 0.520196
H -6.662223 -2.302915 0.840377
H -5.229773 -2.281525 -0.204337
H -5.173586 -1.514239 1.385291

31
Symbol X Y Z
C 0.516632 1.411395 -3.016444
C -0.570695 0.877381 -2.333487
C -0.460803 0.757892 -0.950653
C 0.708612 1.150982 -0.315026
C 1.749464 1.667107 -1.096089
N 1.640234 1.802543 -2.423046
H 0.473008 1.530115 -4.093864
H -1.462714 0.579041 -2.869862
H 0.827526 1.047616 0.755183
C 3.028472 2.102846 -0.480101
C 3.780702 3.110631 -1.089145
C 3.500009 1.508791 0.694360
C 4.977411 3.532058 -0.523503
H 3.415434 3.558082 -2.005049
C 4.702551 1.928700 1.252671
H 2.946598 0.694487 1.149985
C 5.441200 2.942895 0.650045
H 5.548896 4.320783 -0.998033
H 5.067459 1.455643 2.156587
H 6.376416 3.269214 1.089179
O 1.816506 -3.613167 0.989151
S 1.643843 -2.273411 0.426637
O 0.564263 -2.152209 -0.559437
O 1.732891 -1.178299 1.391869
C 3.153130 -2.042393 -0.610747
F 4.241087 -1.953617 0.152910
F 3.060780 -0.927405 -1.333774
F 3.312755 -3.066062 -1.447758
P -1.900031 0.229016 0.018848
C -2.926127 -0.830927 -1.017185
C -4.287615 -0.556472 -1.165037
C -2.338751 -1.917951 -1.676020
C -5.066867 -1.382736 -1.967184
H -4.739200 0.294154 -0.669522
C -3.130978 -2.733559 -2.473359
H -1.279566 -2.120973 -1.558300
C -4.490601 -2.467955 -2.617815
H -6.122203 -1.172221 -2.085125
H -2.683904 -3.576582 -2.984960
H -5.101155 -3.107094 -3.243911
C -1.428141 -0.581733 1.553955
C -0.961283 0.205589 2.610897
C -1.517347 -1.967355 1.683933
C -0.562316 -0.404314 3.791708
H -0.919078 1.285612 2.521581
C -1.120337 -2.565973 2.872799
H -1.883720 -2.576254 0.867329
C -0.639595 -1.787635 3.920456
H -0.196663 0.201462 4.611098
H -1.185406 -3.641575 2.977582
H -0.329974 -2.260538 4.844546
C -2.828742 1.714638 0.456650
C -2.678373 2.890701 -0.279786
C -3.746359 1.648430 1.511083
C -3.449222 4.002225 0.042393
H -1.964339 2.948358 -1.092507
C -4.513139 2.763371 1.820968

H -3.859525 0.736352 2.086823
C -4.363800 3.938143 1.087822
H -3.330122 4.917595 -0.523130
H -5.223223 2.715641 2.636706
H -4.960651 4.807146 1.336064

32

Symbol	X	Y	Z
C	0.077144	0.021186	-0.163646
C	0.619849	-0.053114	-1.563053
C	-0.146698	-0.620708	-2.502856
C	-1.476268	-1.111682	-2.171143
C	-1.822262	-1.380049	-0.899873
N	-0.839138	-1.090318	0.122656
H	0.201329	-0.703748	-3.525011
H	0.905336	-0.004657	0.551544
H	1.622650	0.317269	-1.745568
H	-2.213392	-1.216908	-2.957293
C	-3.159420	-1.810777	-0.454299
C	-3.989235	-2.531847	-1.317432
C	-3.641330	-1.444942	0.807586
C	-5.286404	-2.852718	-0.937727
H	-3.614022	-2.857382	-2.280410
C	-4.938403	-1.767060	1.183462
H	-3.001898	-0.897703	1.492077
C	-5.766922	-2.467293	0.310593
H	-5.919019	-3.414378	-1.614093
H	-5.302311	-1.470439	2.159641
H	-6.777965	-2.720225	0.605600
S	-0.210867	-2.386546	0.975055
O	4.879876	1.423272	0.434383
O	0.648337	-1.857423	2.004638
O	-1.277440	-3.310234	1.254925
C	0.909400	-3.296800	-0.210255
F	1.927539	-2.539969	-0.555763
F	0.219746	-3.645838	-1.282736
F	1.346487	-4.379522	0.407716
S	4.167116	0.286579	-0.148278
O	3.029566	-0.192156	0.647874
O	3.905766	0.387103	-1.585669
C	5.370522	-1.104094	-0.015895
F	6.482807	-0.824771	-0.691833
F	4.848649	-2.223116	-0.510014
F	5.695434	-1.317968	1.256296
P	-0.816626	1.656837	0.108000
C	-2.128206	1.839725	-1.110541
C	-1.783031	2.186628	-2.422845
C	-3.443229	1.483716	-0.796802
C	-2.753000	2.157601	-3.415302
H	-0.765784	2.463577	-2.672354
C	-4.404296	1.455874	-1.798384
H	-3.720158	1.211973	0.213491
C	-4.058270	1.784182	-3.105509
H	-2.487547	2.422956	-4.430698
H	-5.420624	1.171902	-1.555262
H	-4.809590	1.756912	-3.885272
C	-1.501730	1.706281	1.773462
C	-0.980856	0.884646	2.776110
C	-2.501731	2.640327	2.067149
C	-1.489719	0.978374	4.066534
H	-0.189102	0.175270	2.570622
C	-2.998152	2.725912	3.360477
H	-2.895032	3.293893	1.296720
C	-2.498428	1.890707	4.356283
H	-1.093636	0.336770	4.843317
H	-3.775316	3.444014	3.588841
H	-2.892893	1.956889	5.362765
C	0.396378	2.970082	-0.129191
C	1.722204	2.767672	0.261143
C	-0.018072	4.212934	-0.619140
C	2.643118	3.799303	0.123541
H	2.047010	1.820374	0.674184
C	0.907903	5.241450	-0.736980
H	-1.047751	4.380128	-0.912143
C	2.235696	5.032736	-0.374630
H	3.673617	3.619287	0.403290
H	0.590837	6.203884	-1.118098
H	2.954513	5.836114	-0.480150

33

Symbol	X	Y	Z
C	-0.495357	0.185233	-0.203160
C	0.688935	-0.063803	-0.973654
C	1.036311	-1.349174	-1.304973
C	0.026393	-2.358007	-1.192094
C	-1.079908	-2.159192	-0.431446
N	-1.040352	-0.999193	0.439484
H	1.954573	-1.551575	-1.838670

H	1.096317	1.597493	1.301889
H	1.288553	0.759346	-1.345209
H	0.077652	-3.217183	-1.851400
C	-2.346583	-2.899890	-0.512157
C	-2.394515	-4.200341	-1.034685
C	-3.546542	-2.299796	-0.109744
C	-3.605132	-4.865448	-1.169447
H	-1.477622	-4.699360	-1.325486
C	-4.757253	-2.968380	-0.248330
H	-3.530010	-1.299894	0.306931
C	-4.795403	-4.252015	-0.782679
H	-3.619462	-5.871142	-1.572879
H	-5.674621	-2.479621	0.058949
H	-5.738870	-4.773206	-0.890224
S	-0.307077	-1.328098	1.935833
O	4.233609	-0.009510	-1.232865
O	0.686050	-2.382018	1.918712
O	-0.013847	-0.060449	2.586614
C	-1.716345	-2.053557	2.915798
F	-2.012968	-3.259857	2.470649
F	-2.784831	-1.276888	2.850004
F	-1.316709	-2.137398	4.174897
S	4.626339	-1.372342	-0.857485
O	4.213296	-1.790556	0.482998
O	4.443457	-2.374424	-1.907891
C	6.461092	-1.237646	-0.711477
F	6.997938	-0.849697	-1.866495
F	6.994090	-2.411071	-0.376388
F	6.795755	-0.346976	0.222009
P	-1.700180	1.321036	-0.803104
C	-2.810302	0.501471	-1.998136
C	-2.218213	-0.370878	-2.920416
C	-4.200654	0.619562	-1.949362
C	-3.012098	-1.099432	-3.795526
H	-1.142225	-0.509816	-2.931342
C	-4.989364	-0.119646	-2.825380
H	-4.678397	1.259658	-1.219040
C	-4.398493	-0.975189	-3.747597
H	-2.548590	-1.775117	-4.503737
H	-6.067699	-0.032909	-2.775177
H	-5.016939	-1.554635	-4.422372
C	-2.760396	2.086026	0.447203
C	-2.752773	1.643855	1.766393
C	-3.600794	3.139270	0.064063
C	-3.595303	2.238293	2.702252
H	-2.074618	0.861418	2.069622
C	-4.434959	3.730183	1.002151
H	-3.596800	3.502903	-0.958451
C	-4.435967	3.276044	2.320491
H	-3.584043	1.890692	3.728002
H	-5.081461	4.546787	0.706383
H	-5.089245	3.739987	3.049344
C	-0.817731	2.690990	-1.601288
C	-0.547416	3.843598	-0.855978
C	-0.370600	2.588976	-2.920385
C	0.167629	4.887296	-1.431951
H	-0.900202	3.931787	0.165763
C	0.353392	3.633428	-3.484808
H	-0.586634	1.707030	-3.511538
C	0.620133	4.780816	-2.743435
H	0.371121	5.780731	-0.854376
H	0.701510	3.551726	-4.506709
H	1.178738	5.594267	-3.190030
N	1.981819	1.916273	1.727343
C	2.915234	0.733664	1.622824
H	2.474607	-0.043385	2.245319
H	2.887716	0.394902	0.590233
C	2.479633	3.128893	0.987407
H	1.706834	3.890565	1.081785
H	3.356892	3.471020	1.536262
C	1.666960	2.263754	3.158564
H	1.581600	1.319371	3.690401
H	2.521079	2.817311	3.547855
C	0.377200	3.055429	3.273812
H	0.126079	3.162133	4.329535
H	0.456536	4.056561	2.849741
H	-0.440262	2.519255	2.786957
C	4.336990	1.045037	2.047426
H	4.885865	0.104525	2.029739
H	4.831491	1.725802	1.352788
H	4.391321	1.456603	3.057058
C	2.807917	2.879787	-0.472458
H	3.245259	3.795547	-0.873994
H	3.516374	2.063263	-0.615703
H	1.907513	2.676461	-1.052565

$TS_{23/24}$

Symbol	X	Y	Z

C	-1.721342	2.802315	0.078792
C	-2.812819	3.657639	0.108610
C	-4.080817	3.115103	-0.039485
C	-4.203587	1.744798	-0.220697
C	-3.059964	0.949245	-0.253579
N	-1.835850	1.483889	-0.096741
H	-4.960539	3.746245	-0.017304
H	-0.714374	3.178341	0.207836
H	-2.661008	4.718689	0.251865
H	-5.172213	1.278597	-0.346880
C	-3.193866	-0.522489	-0.442126
C	-2.727740	-1.143342	-1.601026
C	-3.833962	-1.283078	0.539008
C	-2.874390	-2.517019	-1.761280
H	-2.254907	-0.550510	-2.372756
C	-3.973590	-2.656496	0.378680
H	-4.204063	-0.797404	1.435247
C	-3.488743	-3.275564	-0.769870
H	-2.511005	-2.993629	-2.663638
H	-4.458758	-3.242120	1.150006
H	-3.596405	-4.346280	-0.895051
S	0.334370	0.385456	-0.184461
O	1.804180	-0.692251	-0.122099
O	0.868407	1.639063	0.250167
O	-0.036351	-0.009187	-1.504584
C	-0.343872	-0.673832	1.270511
F	0.638249	-0.798146	2.135000
F	-0.668656	-1.833922	0.759782
F	-1.357152	-0.110453	1.864512
S	3.204241	-0.469388	0.567922
O	3.143637	0.499929	1.626124
O	3.800509	-1.760711	0.765192
C	4.087569	0.330555	-0.849728
F	4.237263	-0.541941	-1.826142
F	5.268681	0.743164	-0.430224
F	3.380071	1.361521	-1.275458

$TS_{24/32}$

Symbol	X	Y	Z
C	0.235836	-0.975265	0.476193
C	-0.115826	-1.973121	-0.465542
C	-0.791915	-1.623000	-1.589435
C	-1.105616	-0.256479	-1.824600
C	-0.876399	0.698616	-0.884773
N	-0.314711	0.288207	0.337637
H	-1.096019	-2.368310	-2.311137
H	0.499489	-1.250673	1.486649
H	0.184189	-2.991115	-0.256949
H	-1.536584	0.056255	-2.766069
C	-1.148454	2.129836	-1.170562
C	-2.457266	2.554086	-1.388270
C	-0.079059	3.014415	-1.327459
C	-2.692980	3.879626	-1.746062
H	-3.270325	1.845651	-1.267709
C	-0.322251	4.329047	-1.694449
H	0.935065	2.671266	-1.157253
C	-1.631284	4.764036	-1.899177
H	-3.709166	4.217876	-1.908060
H	0.508589	5.013093	-1.820802
H	-1.820418	5.792698	-2.181887
S	-0.358234	1.251503	1.776220
O	-3.710534	-2.218344	-1.719450
O	0.529526	0.595941	2.700383
O	-0.252188	2.643953	1.462370
C	-2.110211	0.969494	2.372730
F	-2.215397	-0.253707	2.826832
F	-2.943545	1.203030	1.388703
F	-2.310549	1.834205	3.349593
S	-4.003870	-1.345128	-0.580126
O	-3.055287	-1.430602	0.529971
O	-4.423153	0.011018	-0.942321
C	-5.539575	-2.079806	0.132104
F	-6.525370	-2.075374	-0.763835
F	-5.942058	-1.388805	1.197540
F	-5.327039	-3.339458	0.510266
P	2.683856	-0.623910	-0.024330
C	3.156925	-1.531026	-1.522141
C	4.191662	-2.470656	-1.526508
C	2.400430	-1.328003	-2.683508
C	4.474773	-3.183522	-2.686218
H	4.778752	-2.640959	-0.631630
C	2.695640	-2.037130	-3.841692
H	1.593329	-0.602310	-2.689941
C	3.730742	-2.967528	-3.842266
H	5.281589	-3.906155	-2.686342
H	2.113306	-1.866914	-4.738818
H	3.956212	-3.525344	-4.742998
C	3.064801	1.133135	-0.302318

C	2.888454	2.004929	0.779963
C	3.436902	1.646856	-1.548597
C	3.071691	3.372511	0.613227
H	2.630927	1.613306	1.758875
C	3.620169	3.016645	-1.706849
H	3.593435	0.984995	-2.391295
C	3.430868	3.881017	-0.631656
H	2.929069	4.038411	1.455287
H	3.913754	3.407599	-2.673365
H	3.568943	4.947372	-0.762566
C	3.807775	-1.177541	1.293277
C	3.444863	-2.309158	2.029556
C	5.017259	-0.530722	1.562097
C	4.289312	-2.794719	3.021728
H	2.509898	-2.821060	1.826109
C	5.853942	-1.017274	2.559869
H	5.304078	0.346416	0.993421
C	5.491576	-2.147292	3.288201
H	4.004952	-3.672643	3.588324
H	6.791075	-0.514976	2.766232
H	6.146654	-2.522112	4.065203

$TS_{25/26}$

Symbol	X	Y	Z
C	-0.713217	-1.086507	1.147396
C	0.129648	-1.845131	0.413523
C	0.568991	-1.387143	-0.860567
C	-0.086180	-0.246811	-1.392113
C	-0.994642	0.474028	-0.670626
N	-1.233503	0.094802	0.649920
H	1.018652	-2.090302	-1.546336
H	-1.053757	-1.369232	2.132485
H	0.460958	-2.790926	0.817023
H	0.062532	0.030968	-2.426906
C	-1.776186	1.540104	-1.343672
C	-3.157373	1.397398	-1.497357
C	-1.108381	2.601846	-1.953440
C	-3.864053	2.326788	-2.245777
H	-3.658728	0.558413	-1.035138
C	-1.825543	3.538114	-2.690488
H	-0.033495	2.694658	-1.846743
C	-3.201790	3.400801	-2.837116
H	-4.933691	2.209657	-2.370145
H	-1.307088	4.369200	-3.152207
H	-3.758870	4.126884	-3.416840
S	-2.086917	1.067877	1.810959
O	-3.606905	-3.540805	0.459281
O	-3.148266	1.813464	1.213064
O	-2.228539	0.278919	2.999438
C	-0.720914	2.281226	2.179099
F	-1.127981	3.076081	3.141709
F	-0.450390	2.983101	1.092205
F	0.354030	1.615118	2.559261
S	-3.709094	-2.180654	-0.064693
O	-3.813128	-1.129613	0.955493
O	-2.804663	-1.861480	-1.170209
C	-5.374528	-2.141389	-0.857501
F	-5.450303	-3.041353	-1.834908
F	-5.614686	-0.938052	-1.380403
F	-6.327799	-2.406229	0.031910
P	2.968697	-0.621277	-0.389852
C	4.382995	-1.480497	-1.150107
C	5.580457	-1.701826	-0.464479
C	4.251951	-1.905136	-2.475860
C	6.636917	-2.338610	-1.105907
H	5.687173	-1.376493	0.564021
C	5.315292	-2.533461	-3.114651
H	3.323726	-1.738567	-3.012934
C	6.506118	-2.752445	-2.428811
H	7.564343	-2.508433	-0.572646
H	5.210481	-2.857242	-4.142781
H	7.332279	-3.247729	-2.924394
C	3.165625	1.130518	-0.824690
C	2.241716	2.038717	-0.295113
C	4.142648	1.585437	-1.714405
C	2.312205	3.387827	-0.621638
H	1.487440	1.690407	0.400171
C	4.199168	2.933614	-2.051341
H	4.862002	0.893461	-2.136414
C	3.289599	3.835183	-1.506011
H	1.603282	4.084382	-0.189817
H	4.962219	3.281330	-2.736943
H	3.343704	4.884912	-1.767166
C	3.221130	-0.751692	1.407159
C	3.098066	-2.030985	1.965437
C	3.472524	0.339833	2.240993
C	3.234853	-2.214466	3.335076
H	2.914569	-2.888977	1.325882

C	3.597678	0.151432	3.614363
H	3.582242	1.334279	1.826121
C	3.478298	-1.120900	4.162504
H	3.146009	-3.208381	3.755956
H	3.795161	1.002866	4.254153
H	3.576853	-1.262222	5.231881

$TS_{27/28}$

Symbol	X	Y	Z
C	0.790925	0.252269	-1.545274
C	-0.343044	-0.425154	-1.399537
C	-1.039583	-0.608925	-0.092605
C	-0.323908	0.145808	0.974240
C	0.783515	0.881290	0.821455
N	1.411126	0.984192	-0.472955
H	-0.836626	-1.993485	0.288731
H	1.361161	0.254299	-2.460583
H	-0.704786	-0.948346	-2.278224
H	-0.637703	0.000862	2.000485
C	1.438869	1.485419	2.009970
C	2.745945	1.148352	2.370857
C	0.698778	2.349809	2.816793
C	3.293211	1.665448	3.537113
H	3.314868	0.476540	1.740333
C	1.254113	2.869509	3.982980
H	-0.306130	2.627570	2.516500
C	2.551598	2.526789	4.344875
H	4.303842	1.395421	3.819794
H	0.674831	3.544596	4.601334
H	2.986811	2.929928	5.251584
S	2.134497	2.398051	-0.970001
O	4.636253	0.319873	-0.463294
O	2.828462	3.035467	0.115008
O	2.754875	2.162589	-2.248461
C	0.684853	3.516987	-1.341415
F	1.097457	4.772565	-1.383195
F	-0.232886	3.388289	-0.394111
F	0.166374	3.186649	-2.512039
S	4.100441	-1.039055	-0.547505
O	3.580197	-1.571142	0.715774
O	3.279397	-1.320249	-1.725461
C	5.600731	-2.075883	-0.838649
F	6.210051	-1.719625	-1.968655
F	5.266812	-3.363190	-0.934687
F	6.471177	-1.946188	0.161593
P	-2.767526	-0.106018	-0.179727
C	-3.634807	-0.417756	1.387023
C	-4.740791	-1.273134	1.403656
C	-3.213471	0.186181	2.579735
C	-5.396745	-1.542135	2.599613
H	-5.089984	-1.740749	0.490960
C	-3.874009	-0.088971	3.770171
H	-2.388287	0.886339	2.590950
C	-4.960987	-0.957875	3.782863
H	-6.248342	-2.210745	2.601503
H	-3.539869	0.382167	4.685880
H	-5.471719	-1.171775	4.713649
C	-2.987989	1.640019	-0.625117
C	-2.669214	2.016828	-1.934988
C	-3.423110	2.600644	0.290350
C	-2.795898	3.344166	-2.322354
H	-2.336880	1.277196	-2.655334
C	-3.533172	3.930039	-0.103642
H	-3.700576	2.322665	1.298617
C	-3.222326	4.301506	-1.406781
H	-2.553947	3.630368	-3.338171
H	-3.873926	4.671271	0.608404
H	-3.316967	5.336599	-1.711599
C	-3.602581	-1.049045	-1.476190
C	-3.080965	-2.264504	-1.918461
C	-4.809694	-0.572851	-1.998023
C	-3.763282	-3.006390	-2.875872
H	-2.141157	-2.628944	-1.523765
C	-5.489635	-1.320871	-2.950038
H	-5.216503	0.374561	-1.661746
C	-4.967711	-2.535697	-3.387831
H	-3.350943	-3.946463	-3.220560
H	-6.425304	-0.953596	-3.352223
H	-5.499797	-3.113667	-4.133409
N	-0.350244	-3.143129	0.732236
C	0.974422	-3.350600	0.083300
H	1.547851	-2.433426	0.216136
H	1.500370	-4.150198	0.618254
C	-0.226614	-2.968384	2.214351
H	-1.092997	-2.379846	2.532618
H	-0.322613	-3.957330	2.674795
C	-1.268836	-4.274686	0.447617
H	-1.357087	-4.374632	-0.633340

H	-0.797850	-5.193660	0.817730
C	-2.651218	-4.095185	1.053218
H	-3.301803	-4.896994	0.701325
H	-2.639613	-4.122772	2.143144
H	-3.090900	-3.148032	0.740532
C	0.899335	-3.689542	-1.397903
H	1.896131	-3.553708	-1.819047
H	0.579712	-4.715717	-1.581369
H	0.231761	-3.010023	-1.932592
C	1.063762	-2.324653	2.708131
H	0.948982	-2.126803	3.775660
H	1.920001	-2.985504	2.576289
H	1.296582	-1.386824	2.207330

$TS_{29/30}$

Symbol	X	Y	Z
C	-0.562821	-0.922692	-2.503266
C	0.674624	-1.214219	-2.026422
C	1.054465	-0.739043	-0.735213
C	0.266518	0.292840	-0.165603
C	-0.971785	0.568023	-0.686417
N	-1.512666	-0.353844	-1.622904
H	-5.082729	-0.443915	0.212040
H	-0.905499	-1.158051	-3.500960
H	1.391694	-1.719582	-2.666821
H	0.695797	0.939788	0.592714
C	-1.804357	1.736354	-0.353797
C	-2.893694	2.085264	-1.164641
C	-1.518502	2.550114	0.752927
C	-3.654068	3.217342	-0.892992
H	-3.123158	1.471724	-2.027340
C	-2.282505	3.678316	1.020977
H	-0.675628	2.319940	1.392743
C	-3.355400	4.023300	0.202025
H	-4.477835	3.479746	-1.549137
H	-2.035082	4.294847	1.877758
H	-3.947497	4.905751	0.413117
S	-2.579615	-1.613751	-0.841005
O	3.526560	4.288307	0.878773
O	-3.366119	-0.916236	0.188004
O	-3.278168	-2.297348	-1.924922
C	-1.816730	-3.042731	0.140284
F	-0.932991	-2.587742	1.013325
F	-1.225687	-3.885912	-0.693651
F	-2.775397	-3.686390	0.794904
S	2.485964	3.380484	0.391180
O	2.919006	2.449249	-0.654615
O	1.632006	2.807437	1.431558
C	1.342752	4.504085	-0.527520
F	0.703078	5.319646	0.310806
F	0.436093	3.809906	-1.209773
F	2.023442	5.255589	-1.395255
P	2.580564	-1.241473	-0.018408
C	3.914013	-1.165529	-1.241313
C	4.893033	-2.159299	-1.305874
C	3.952319	-0.071409	-2.110910
C	5.915683	-2.053506	-2.242314
H	4.857418	-3.012712	-0.638884
C	4.980155	0.024370	-3.040991
H	3.199175	0.704384	-2.036443
C	5.958271	-0.964625	-3.107447
H	6.674420	-2.824092	-2.297980
H	5.016212	0.871252	-3.715082
H	6.755010	-0.887368	-3.837528
C	2.982955	-0.235167	1.421574
C	2.134300	-0.296776	2.531297
C	4.126358	0.559448	1.445932
C	2.432492	0.448588	3.662567
H	1.250514	-0.926739	2.512046
C	4.419516	1.299030	2.586819
H	4.774569	0.612148	0.579839
C	3.575393	1.243678	3.689374
H	1.776007	0.407207	4.522755
H	5.305288	1.921543	2.608673
H	3.807005	1.823319	4.574912
C	2.522515	-2.960614	0.571505
C	1.766313	-3.897570	-0.133551
C	3.265720	-3.354896	1.686876
C	1.745063	-5.223506	0.281124
H	1.180998	-3.593847	-0.992674
C	3.244221	-4.684750	2.093216
H	3.854476	-2.630622	2.238278
C	2.484187	-5.616785	1.393080
H	1.148197	-5.945973	-0.261792
H	3.817985	-4.989963	2.959421
H	2.465017	-6.650394	1.716685
N	-6.077421	-0.182477	0.339398
C	-6.755650	-0.288350	-0.997819

H	-6.995293	-1.339158	-1.151936
H	-7.690693	0.267908	-0.912371
C	-6.671361	-1.151672	1.325509
H	-6.437894	-0.782975	2.322374
H	-7.752784	-1.105380	1.188235
C	-6.096291	1.225876	0.866998
H	-5.973193	1.891181	0.013886
H	-7.089515	1.383584	1.290730
C	-4.982369	1.459288	1.870633
H	-5.032764	2.492893	2.213070
H	-5.065598	0.809776	2.743161
H	-4.008575	1.307718	1.399514
C	-5.880054	0.222836	-2.126799
H	-6.422427	0.108548	-3.065596
H	-5.629057	1.277427	-2.011903
H	-4.958757	-0.358940	-2.195755
C	-6.122035	-2.554518	1.137590
H	-6.614895	-3.221176	1.845207
H	-6.303481	-2.939958	0.133158
H	-5.049460	-2.574961	1.331654

$TS_{32/33}$

Symbol	X	Y	Z
C	-1.707075	-0.264359	-0.471752
C	-2.046100	-0.370450	-1.928795
C	-1.891844	0.686660	-2.743837
C	-1.299930	1.906200	-2.233348
C	-1.278047	2.164001	-0.912656
N	-1.858977	1.152437	-0.045037
H	-2.170138	0.629442	-3.788854
H	-2.473058	-1.355606	0.120607
H	-2.464576	-1.294451	-2.306095
H	-0.808042	2.584151	-2.919323
C	-0.613590	3.328309	-0.296173
C	-0.448798	4.507844	-1.029322
C	-0.081805	3.255895	0.996117
C	0.265852	5.573904	-0.497557
H	-0.894736	4.600155	-2.012390
C	0.632959	4.322030	1.524612
H	-0.218689	2.356899	1.585565
C	0.816110	5.482715	0.777604
H	0.384230	6.481105	-1.077496
H	1.048774	4.244616	2.522046
H	1.373531	6.314407	1.190939
S	-3.222119	1.632879	0.766636
O	6.838259	0.043120	1.174797
O	-3.676302	0.540486	1.595571
O	-3.021405	2.951433	1.311202
C	-4.561942	1.883271	-0.511503
F	-4.733453	0.788882	-1.232704
F	-4.242943	2.890328	-1.303358
F	-5.683127	2.161690	0.131260
S	5.652997	0.416605	0.401795
O	4.539011	0.936204	1.198985
O	5.924245	1.135708	-0.841653
C	5.014697	-1.215751	-0.176405
F	5.933043	-1.864398	-0.888452
F	3.927953	-1.068223	-0.934888
F	4.679358	-1.981189	0.866374
P	0.078533	-0.679223	-0.189512
C	1.253361	0.242775	-1.229288
C	1.411837	-0.113125	-2.573356
C	1.974646	1.322433	-0.711854
C	2.268927	0.614764	-3.387796
H	0.883071	-0.953747	-2.998631
C	2.843900	2.032112	-1.528828
H	1.891030	1.608030	0.326278
C	2.985480	1.687904	-2.867462
H	2.384106	0.332339	-4.426858
H	3.422350	2.843403	-1.106504
H	3.667728	2.242142	-3.500495
C	0.493758	-0.360912	1.537980
C	-0.504246	-0.171708	2.497824
C	1.840463	-0.322773	1.910876
C	-0.150420	0.068491	3.819706
H	-1.550894	-0.211427	2.226288
C	2.186128	-0.078482	3.233298
H	2.626229	-0.448409	1.178052
C	1.191842	0.119317	4.186539
H	-0.926040	0.218687	4.560284
H	3.234045	-0.029599	3.500422
H	1.461689	0.315617	5.217151
C	0.332243	-2.444521	-0.537258
C	-0.038690	-3.001079	-1.766758
C	0.932049	-3.254907	0.432757
C	0.169410	-4.352780	-2.010709
H	-0.502564	-2.399266	-2.536750
C	1.129809	-4.607825	0.183116

H	1.236852	-2.842897	1.386616
C	0.746022	-5.158912	-1.034947
H	-0.128360	-4.773751	-2.962986
H	1.585967	-5.228285	0.944093
H	0.900546	-6.213686	-1.225730
N	-3.269850	-2.420144	0.464964
C	-4.622054	-1.986807	0.007197
H	-4.821836	-1.037783	0.498455
H	-4.547180	-1.789394	-1.063668
C	-2.891136	-3.746711	-0.111924
H	-1.825717	-3.888815	0.068796
H	-3.416007	-4.517403	0.463537
C	-3.244193	-2.519897	1.955113
H	-3.413649	-1.515696	2.339325
H	-4.082972	-3.144264	2.273277
C	-1.956260	-3.107661	2.515667
H	-1.925816	-2.922966	3.590689
H	-1.891281	-4.184338	2.356420
H	-1.073135	-2.645289	2.077531
C	-5.772438	-2.950123	0.289630
H	-6.663260	-2.567028	-0.210591
H	-5.588506	-3.956678	-0.087286
H	-6.000526	-3.016868	1.353188
C	-3.193001	-3.949605	-1.588821
H	-2.813693	-4.933048	-1.872256
H	-4.259899	-3.927808	-1.809025
H	-2.695322	-3.222788	-2.227765

CF_3-PyH

Symbol	X	Y	Z
C	0.177811	3.464834	-1.150715
C	-0.664563	2.503462	-0.601417
C	-0.171253	1.763821	0.465978
C	1.113174	2.004190	0.943301
C	1.867490	3.010008	0.334113
N	1.400296	3.719758	-0.700169
H	-0.156105	4.054625	-1.997568
H	-1.651075	2.331936	-1.012600
H	1.546058	1.411095	1.739170
C	3.238237	3.319249	0.806703
C	4.217310	3.692715	-0.117162
C	3.563954	3.241223	2.162015
C	5.505280	3.981152	0.313838
H	3.959519	3.720019	-1.168244
C	4.853129	3.537865	2.590998
H	2.804490	2.973033	2.888482
C	5.825897	3.906951	1.667597
H	6.264376	4.257139	-0.408295
H	5.095048	3.485812	3.645693
H	6.831350	4.134964	2.000956
O	3.577560	1.791953	-2.805605
S	2.863258	0.713127	-2.126653
O	3.404341	0.319339	-0.823554
O	1.401195	0.798315	-2.179531
C	3.226565	-0.763363	-3.174697
F	2.775902	-0.591514	-4.416245
F	2.639788	-1.853311	-2.670371
F	4.536720	-0.994016	-3.236324
P	-1.168580	0.442837	1.180642
C	-2.825184	0.403842	0.567071
C	-3.257912	-0.696461	-0.190529
C	-3.707920	1.481090	0.772822
C	-4.530645	-0.734033	-0.713407
H	-2.588511	-1.530297	-0.369053
C	-4.980738	1.452310	0.254446
H	-3.396124	2.352494	1.337407
C	-5.433994	0.339331	-0.504311
H	-4.840718	-1.600257	-1.280939
H	-5.634116	2.298055	0.417907
C	-0.334826	-1.108563	1.116806
C	0.860891	-1.237609	0.392367
C	-0.871092	-2.233075	1.773048
C	1.513393	-2.447380	0.334304
H	1.287737	-0.394229	-0.135044
C	-0.227376	-3.446427	1.711980
H	-1.800451	-2.158709	2.327528
C	0.987728	-3.590184	0.986719
H	2.441676	-2.505384	-0.216673
H	-0.663488	-4.299796	2.212761
C	-1.295720	0.916175	3.006087
F	-0.095947	0.883295	3.583630
F	-1.793117	2.145081	3.152870
F	-2.092632	0.064266	3.649310
N	1.617923	-4.777903	0.917495
N	-6.680524	0.307275	-1.010235
C	2.806405	-5.016948	0.093539
C	1.187699	-5.977511	1.639040
C	2.370017	-6.931087	1.465264

C	2.930318	-6.540466	0.095249
H	2.667517	-4.607905	-0.909757
H	3.685078	-4.538734	0.541557
H	0.988657	-5.746587	2.687766
H	0.272080	-6.385454	1.194432
H	3.114204	-6.737340	2.241349
H	2.069570	-7.976064	1.527510
H	3.956155	-6.870674	-0.061527
H	2.306148	-6.960510	-0.697053
C	-7.696466	1.330431	-0.747409
C	-7.174491	-0.756264	-1.889305
C	-8.488930	-0.186444	-2.422447
C	-8.982119	0.685981	-1.265590
H	-7.745186	1.559831	0.319096
H	-7.460567	2.252998	-1.290464
H	-6.455805	-0.962470	-2.685214
H	-7.335199	-1.679207	-1.319827
H	-8.290829	0.434018	-3.299577
H	-9.192587	-0.968382	-2.704116
H	-9.721042	1.426614	-1.567875
H	-9.418754	0.058940	-0.484692

CF_3-PyH-OH

Symbol	X	Y	Z
C	-0.391585	-4.317071	-0.899245
C	0.273995	-3.120836	-0.802782
C	-0.449816	-1.972961	-0.460749
C	-1.819157	-2.061588	-0.240725
C	-2.464309	-3.286960	-0.350856
N	-1.717106	-4.363965	-0.672552
H	0.088375	-5.254469	-1.140328
H	1.342099	-3.095299	-0.978481
H	-2.405408	-1.185539	0.005650
C	-3.907446	-3.473395	-0.133928
C	-4.636440	-4.369856	-0.921298
C	-4.548637	-2.740227	0.868875
C	-5.997684	-4.531442	-0.701542
H	-4.157265	-4.913774	-1.727986
C	-5.908689	-2.911514	1.085123
H	-3.979389	-2.062358	1.494255
C	-6.633234	-3.805275	0.301247
H	-6.562772	-5.217183	-1.319799
H	-6.401409	-2.351816	1.869886
H	-7.695027	-3.934436	0.470731
P	0.409068	-0.335021	-0.345521
C	2.214910	-0.278132	-0.228429
C	2.896303	0.627421	-1.057818
C	2.977328	-1.077517	0.634602
C	4.270044	0.748614	-1.017932
H	2.334181	1.254030	-1.739696
C	4.355821	-0.994069	0.659464
H	2.504304	-1.795920	1.290971
C	5.044454	-0.069167	-0.160233
H	4.753009	1.481443	-1.650279
H	4.909006	-1.653038	1.314934
C	-0.600592	1.163406	-0.263462
C	-1.656062	1.357746	-1.163449
C	-0.330611	2.173032	0.667290
C	-2.435773	2.497782	-1.118474
H	-1.867771	0.607417	-1.914900
C	-1.078786	3.333844	0.702306
H	0.490967	2.070553	1.365515
C	-2.165335	3.524043	-0.183395
H	-3.259790	2.599588	-1.811918
H	-0.817685	4.106354	1.413060
C	0.214007	-0.605088	1.669640
F	-1.050424	-0.354869	2.089258
F	0.450968	-1.890963	2.056876
F	1.017306	0.150614	2.449850
N	-2.915360	4.652192	-0.141828
N	6.393712	0.027672	-0.128278
C	-3.976679	4.956127	-1.100428
C	-2.753785	5.697010	0.868134
C	-4.000911	6.561205	0.677524
C	-4.288759	6.426560	-0.820070
H	-3.633008	4.792773	-2.124843
H	-4.852325	4.318063	-0.927296
H	-2.698227	5.263099	1.869560
H	-1.835278	6.270727	0.692202
H	-4.828720	6.142363	1.254819
H	-3.843684	7.590817	0.996240
H	-5.310530	6.691370	-1.088971
H	-3.605094	7.061264	-1.389029
C	7.238821	-0.715368	0.806309
C	7.168479	0.881724	-1.028504
C	8.604430	0.402264	-0.813375
C	8.603788	-0.043962	0.651035
H	6.850794	-0.638310	1.824791

H	7.278409	-1.777244	0.533680
H	6.838384	0.755064	-2.062358
H	7.051405	1.938292	-0.758039
H	8.806677	-0.452751	-1.463002
H	9.336383	1.179821	-1.027714
H	9.425192	-0.715137	0.898261
H	8.659733	0.828604	1.306276
H	-2.176471	-5.270391	-0.714025
O	0.390269	-0.245198	-2.075282
H	1.004843	-0.859520	-2.491472

CF_3-PyH+TfO-TS

Symbol	X	Y	Z
C	0.326209	-3.770738	-1.012431
C	-0.495648	-2.835087	-0.455622
C	-0.069361	-1.481380	-0.400775
C	1.269935	-1.200800	-0.760606
C	2.049552	-2.171918	-1.326801
N	1.551357	-3.429161	-1.459401
H	0.053141	-4.811884	-1.109253
H	-1.459210	-3.142461	-0.073606
H	1.691463	-0.215812	-0.621758
C	3.435373	-1.927347	-1.762372
C	4.449906	-2.825850	-1.427462
C	3.732527	-0.767881	-2.481169
C	5.758541	-2.560571	-1.812240
H	4.221862	-3.694148	-0.819800
C	5.042034	-0.510346	-2.864703
H	2.933586	-0.084159	-2.746209
C	6.055036	-1.405542	-2.530128
H	6.548942	-3.248275	-1.538148
H	5.272030	0.385908	-3.427386
H	7.076699	-1.200743	-2.826269
P	-1.038901	-0.294633	0.512976
C	-2.803145	-0.457141	0.343732
C	-3.580679	0.207109	1.304466
C	-3.454774	-1.221514	-0.630802
C	-4.957357	0.121780	1.293080
H	-3.100373	0.803354	2.072973
C	-4.829930	-1.331811	-0.643355
H	-2.890309	-1.742112	-1.394487
C	-5.623892	-0.657685	0.316386
H	-5.525814	0.664653	2.035815
H	-5.299839	-1.947072	-1.398402
C	-0.428359	1.368483	0.338077
C	0.789293	1.719125	0.941789
C	-1.155595	2.355512	-0.337865
C	1.284111	3.003413	0.842724
H	1.365202	0.977991	1.489030
C	-0.678981	3.646603	-0.432065
H	-2.106940	2.106672	-0.792668
C	0.562620	4.005953	0.148196
H	2.236221	3.237595	1.299328
H	-1.271912	4.392252	-0.944174
C	-1.015477	-0.292658	-2.138135
F	0.061823	0.401535	-2.606841
F	-1.095031	-1.389691	-2.953517
F	-2.090181	0.468578	-2.511604
N	1.038448	5.267990	0.050745
N	-6.972590	-0.754325	0.300119
C	2.270879	5.723920	0.693053
C	0.413085	6.309022	-0.766022
C	1.481340	7.402508	-0.821486
C	2.215590	7.240617	0.512105
H	2.290929	5.430515	1.745136
H	3.149321	5.290960	0.198520
H	0.162575	5.923353	-1.757272
H	-0.509587	6.668738	-0.295092
H	2.165719	7.205999	-1.650154
H	1.050018	8.393274	-0.958013
H	3.206133	7.693477	0.517232
H	1.626953	7.684793	1.318370
C	-7.724170	-1.457693	-0.739594
C	-7.841086	-0.173873	1.324738
C	-9.198743	-0.815099	1.035430
C	-9.167027	-1.022976	-0.480904
H	-7.374284	-1.168058	-1.733224
H	-7.605162	-2.543201	-0.636343
H	-7.473161	-0.413575	2.325146
H	-7.881114	0.917752	1.225821
H	-9.263893	-1.780609	1.542777
H	-10.028454	-0.193155	1.368662
H	-9.889395	-1.759129	-0.830983
H	-9.361642	-0.076425	-0.990820
H	2.137126	-4.139119	-1.883352
O	-0.851526	-0.672067	2.073363
H	0.076906	-0.880762	2.330364
O	1.714526	-1.153349	2.375014

S	2.848347	-2.099542	2.354320
O	3.258873	-2.572216	3.670313
O	2.774662	-3.111527	1.306329
C	4.223771	-0.986497	1.832584
F	3.910061	-0.338827	0.711566
F	5.334977	-1.683752	1.620659
F	4.465016	-0.082346	2.777866

CF_3-PyH-TS

Symbol	X	Y	Z
C	-0.454264	-4.285962	-0.435750
C	0.234893	-3.141596	-0.699824
C	-0.443865	-1.891900	-0.625370
C	-1.860518	-1.923878	-0.532507
C	-2.503738	-3.097735	-0.242699
N	-1.780256	-4.249051	-0.176459
H	0.006307	-5.263414	-0.433329
H	1.286722	-3.203734	-0.938588
H	-2.446698	-1.019977	-0.621071
C	-3.957533	-3.180332	-0.007084
C	-4.709198	-4.243024	-0.515660
C	-4.583041	-2.178163	0.739344
C	-6.077219	-4.297567	-0.280582
H	-4.237710	-5.009439	-1.120887
C	-5.950692	-2.239697	0.971837
H	-3.990475	-1.371905	1.156705
C	-6.698265	-3.297812	0.462385
H	-6.658886	-5.116445	-0.684909
H	-6.431072	-1.466316	1.557988
H	-7.764888	-3.343773	0.644702
P	0.395678	-0.391193	-1.067866
C	2.120654	-0.352146	-0.633197
C	2.908424	0.606939	-1.289626
C	2.747141	-1.216508	0.275456
C	4.263003	0.706343	-1.055195
H	2.458163	1.308475	-1.985290
C	4.103352	-1.140907	0.508259
H	2.176195	-1.958509	0.819860
C	4.904674	-0.174614	-0.149737
H	4.831069	1.473597	-1.562840
H	4.554832	-1.834812	1.203823
C	-0.474299	1.112133	-0.710191
C	-1.623216	1.441813	-1.449033
C	-0.016131	2.014167	0.261569
C	-2.311002	2.610887	-1.207377
H	-1.981752	0.778396	-2.229281
C	-0.687719	3.191301	0.505641
H	0.881534	1.790389	0.824811
C	-1.864974	3.520597	-0.214578
H	-3.200620	2.827994	-1.782301
H	-0.297961	3.876771	1.245628
C	-0.058888	-1.001141	1.421063
F	-1.301133	-0.572581	1.765849
F	0.077704	-2.216237	2.021710
F	0.791664	-0.189593	2.110484
N	-2.532197	4.666906	0.028732
N	6.231793	-0.093290	0.081908
C	-3.687690	5.117701	-0.750212
C	-2.188375	5.596010	1.107595
C	-3.400016	6.526424	1.165042
C	-3.876452	6.562906	-0.289150
H	-3.483858	5.045355	-1.820812
H	-4.567353	4.503462	-0.523937
H	-2.031216	5.057284	2.044579
H	-1.269953	6.144041	0.865889
H	-4.172054	6.084386	1.799308
H	-3.147231	7.508762	1.561460
H	-4.906575	6.899202	-0.397833
H	-3.232506	7.222192	-0.876024
C	6.932970	-0.893778	1.087750
C	7.127722	0.817693	-0.632708
C	8.517808	0.319403	-0.237328
C	8.301376	-0.217793	1.179581
H	6.395830	-0.876386	2.038766
H	7.018681	-1.936496	0.759388
H	6.955931	0.762545	-1.710028
H	6.963423	1.852770	-0.309711
H	8.818753	-0.493514	-0.902446
H	9.268862	1.106261	-0.290930
H	9.080436	-0.906112	1.504183
H	8.253646	0.611592	1.889215
H	-2.255586	-5.109761	0.068740
O	0.332595	-0.506055	-2.696789
H	0.571057	0.288609	-3.190491

P1

Symbol	X	Y	Z
P	0.121556	0.212013	-1.035619

C	0.713540	-1.428112	-0.459210
C	0.429222	-1.945680	0.810076
C	1.496500	-2.170891	-1.344022
C	0.931263	-3.184513	1.184248
H	-0.188321	-1.381722	1.499370
C	1.997685	-3.414295	-0.966426
H	1.715037	-1.776887	-2.330383
C	1.715831	-3.919451	0.296687
H	0.710368	-3.579596	2.168458
H	2.603642	-3.984728	-1.659692
H	2.103375	-4.887158	0.592050
C	-1.581382	0.281643	-0.340741
C	-2.391793	-0.855504	-0.404232
C	-2.127257	1.481079	0.126243
C	-3.717335	-0.798819	0.012427
H	-1.987031	-1.792492	-0.770410
C	-3.451050	1.530562	0.549190
H	-1.526661	2.381856	0.168271
C	-4.249401	0.392768	0.493758
H	-4.332522	-1.689160	-0.038038
H	-3.857923	2.463359	0.920685
H	-5.280760	0.435206	0.822159
C	1.003389	1.244374	0.280226
F	2.323778	1.021751	0.224060
F	0.819361	2.560599	0.058374
F	0.621943	1.017270	1.546255

P1-TS

Symbol	X	Y	Z
C	-0.583229	-1.559192	0.482926
C	0.133038	-1.961690	-0.578821
C	0.633422	-0.993035	-1.521145
C	0.031862	0.311083	-1.442787
C	-0.752360	0.687530	-0.400358
N	-0.950172	-0.227011	0.644213
H	0.887093	-1.348811	-2.512946
H	-0.959054	-2.236810	1.235000
H	0.316227	-3.018032	-0.716246
H	0.123927	0.994405	-2.276048
C	-1.482039	1.976315	-0.458059
C	-2.878702	1.990338	-0.424861
C	-0.776064	3.155821	-0.694698
C	-3.557775	3.184125	-0.616076
H	-3.417137	1.068024	-0.259043
C	-1.462810	4.351804	-0.871389
H	0.306883	3.134572	-0.735403
C	-2.853019	4.366251	-0.832758
H	-4.640803	3.188987	-0.599097
H	-0.911296	5.268122	-1.042120
H	-3.388676	5.297008	-0.975991
S	-1.515113	0.194322	2.210560
O	-3.747784	-3.213419	-0.857544
O	-2.516172	1.213352	2.177712
O	-1.635997	-1.022516	2.959866
C	0.032979	1.014481	2.842365
F	-0.143096	1.308668	4.111236
F	0.265366	2.117520	2.148514
F	1.059038	0.188077	2.707298
S	-3.719322	-1.764675	-0.673043
O	-3.597381	-1.319414	0.721428
O	-2.882120	-1.024891	-1.619190
C	-5.428390	-1.231121	-1.121068
F	-5.701301	-1.540564	-2.386533
F	-5.558948	0.089921	-0.980175
F	-6.330085	-1.822214	-0.341761
P	2.866610	-0.648408	-0.992664
C	3.213623	1.117644	-0.984507
C	2.684042	1.846285	0.087290
C	3.859852	1.784536	-2.030445
C	2.834887	3.226035	0.135415
H	2.179731	1.332224	0.896528
C	3.997412	3.166109	-1.976888
H	4.263079	1.245464	-2.877867
C	3.490758	3.886099	-0.898846
H	2.432760	3.780053	0.975034
H	4.507050	3.680703	-2.781609
H	3.607858	4.962151	-0.865192
C	3.415150	-1.371810	0.569692
C	3.047376	-2.697878	0.823992
C	4.156607	-0.654937	1.514962
C	3.418871	-3.301650	2.018278
H	2.494528	-3.265223	0.086478
C	4.514486	-1.265549	2.709609
H	4.461493	0.365342	1.319502
C	4.142760	-2.582789	2.963636
H	3.138255	-4.329498	2.209612
H	5.089892	-0.711940	3.440830
H	4.423028	-3.051572	3.898892

C	4.072270	-1.377432	-2.243117
F	3.729809	-1.009114	-3.483823
F	4.022501	-2.708376	-2.182500
F	5.333799	-0.999854	-2.038074

P1-TS-P

Symbol	X	Y	Z
C	-0.642879	-1.582278	0.131259
C	0.188853	-1.761251	-0.888407
C	0.909801	-0.601304	-1.502442
C	0.214781	0.692571	-1.202023
C	-0.650130	0.837632	-0.192058
N	-0.939876	-0.290828	0.626379
H	1.001340	-0.723437	-2.587191
H	-1.195731	-2.383458	0.599817
H	0.307176	-2.749864	-1.311590
H	0.332748	1.510045	-1.900341
C	-1.403797	2.103201	-0.015637
C	-2.800387	2.094735	0.012302
C	-0.715886	3.314872	0.023924
C	-3.495393	3.292766	0.067624
H	-3.324555	1.149851	-0.015673
C	-1.417661	4.514818	0.091023
H	0.367997	3.319559	0.007230
C	-2.807303	4.504528	0.110489
H	-4.578623	3.280246	0.078021
H	-0.877775	5.452991	0.129907
H	-3.355418	5.437767	0.160521
S	-1.372923	-0.184225	2.235431
O	-3.893036	-2.845713	-1.451159
O	-2.242919	0.926794	2.488229
O	-1.636452	-1.512889	2.715347
C	0.258527	0.300976	2.989114
F	0.068373	0.683067	4.236072
F	0.793122	1.303674	2.298478
F	1.077318	-0.734139	2.957119
S	-3.808690	-1.461818	-0.987359
O	-3.681994	-1.301920	0.465436
O	-2.942202	-0.586814	-1.778368
C	-5.493478	-0.786391	-1.321396
F	-5.770986	-0.826881	-2.623365
F	-5.575344	0.484535	-0.920757
F	-6.424788	-1.484208	-0.675252
P	2.718012	-0.523459	-0.988470
C	3.392876	1.109215	-1.282209
C	2.960013	2.136400	-0.431770
C	4.279632	1.376594	-2.328675
C	3.418850	3.429485	-0.634878
H	2.281224	1.926901	0.387565
C	4.730131	2.677489	-2.517924
H	4.625650	0.596478	-2.993470
C	4.300758	3.698688	-1.678162
H	3.089768	4.223927	0.022980
H	5.418912	2.888540	-3.325547
H	4.657023	4.709314	-1.834113
C	3.120975	-1.134238	0.647561
C	2.716983	-2.423988	1.021951
C	3.901777	-0.354803	1.509214
C	3.090916	-2.918570	2.262671
H	2.128424	-3.042824	0.359212
C	4.260092	-0.863200	2.749926
H	4.242410	0.629658	1.216326
C	3.854981	-2.139383	3.126007
H	2.781886	-3.914157	2.553434
H	4.862336	-0.261572	3.418259
H	4.139473	-2.531281	4.094702
C	3.554709	-1.714581	-2.184063
F	3.312683	-1.353043	-3.442777
F	3.055246	-2.935131	-1.998428
F	4.866251	-1.754974	-1.995891

P1-TS-R

Symbol	X	Y	Z
C	-0.283337	-1.114403	-0.466389
C	0.218132	-0.869304	-1.715508
C	0.085673	0.409570	-2.255647
C	-0.602508	1.378303	-1.550140
C	-1.177397	1.095784	-0.315806
N	-0.938323	-0.138014	0.220402
H	0.485373	0.632866	-3.236193
H	-0.202660	-2.073024	0.026743
H	0.704496	-1.670019	-2.254799
H	-0.779471	2.359001	-1.970399
C	-2.072024	2.095371	0.310223
C	-3.427754	1.806180	0.478481
C	-1.583607	3.375014	0.577474
C	-4.283838	2.797166	0.936359
H	-3.798423	0.817066	0.248571

C	-2.444555	4.355373	1.054110
H	-0.534082	3.593732	0.420860
C	-3.794143	4.066578	1.231530
H	-5.336572	2.575360	1.059497
H	-2.062752	5.343205	1.279035
H	-4.466916	4.834222	1.594145
S	-1.336397	-0.588837	1.921822
O	-3.315649	-2.887642	-2.014097
O	-2.504402	0.084293	2.380848
O	-1.090313	-1.993122	2.027717
C	0.145516	0.252270	2.693505
F	0.039424	0.122064	3.994125
F	0.161673	1.526242	2.357695
F	1.232389	-0.346230	2.255184
S	-3.548307	-1.635306	-1.303456
O	-3.281050	-1.680646	0.142246
O	-3.035505	-0.428536	-1.957487
C	-5.378762	-1.415589	-1.374579
F	-5.792056	-1.345927	-2.636523
F	-5.738280	-0.289732	-0.755779
F	-5.997945	-2.432221	-0.783174
P	3.751680	-0.727098	-1.379360
C	3.124336	0.995102	-1.199158
C	2.682416	1.547377	0.003255
C	3.034725	1.751147	-2.373338
C	2.166245	2.840112	0.030586
H	2.742962	0.976850	0.920676
C	2.525557	3.044879	-2.341834
H	3.356911	1.324711	-3.318301
C	2.085119	3.589972	-1.139145
H	1.834137	3.262187	0.972072
H	2.466890	3.622438	-3.256363
H	1.684772	4.596645	-1.113458
C	3.601153	-1.469204	0.290257
C	2.752770	-2.578186	0.378283
C	4.263306	-1.027824	1.442736
C	2.543997	-3.219334	1.595435
H	2.267234	-2.956126	-0.515641
C	4.068382	-1.682536	2.653125
H	4.930115	-0.175958	1.402234
C	3.204538	-2.771644	2.733476
H	1.877868	-4.071790	1.648816
H	4.590457	-1.337316	3.537225
H	3.051583	-3.273018	3.681438
C	5.587359	-0.281195	-1.383176
F	5.921877	0.216009	-2.586231
F	6.343151	-1.367170	-1.173793
F	5.963539	0.638871	-0.479388

P2

Symbol	X	Y	Z
P	-0.052833	-0.067249	-1.285682
C	0.493758	-1.622243	-0.484662
C	0.029161	-2.078867	0.756153
C	1.435392	-2.390979	-1.166594
C	0.501895	-3.260282	1.293322
H	-0.714501	-1.510492	1.302471
C	1.930141	-3.578045	-0.632065
H	1.796659	-2.064959	-2.136364
C	1.459853	-4.016673	0.605622
H	0.147605	-3.625484	2.249428
H	2.662924	-4.145819	-1.188380
C	-1.620896	0.360731	-0.447833
C	-2.778623	0.147531	-1.196312
C	-1.759646	0.875173	0.852232
C	-4.045065	0.407018	-0.679543
H	-2.700085	-0.228848	-2.210750
C	-3.009377	1.152381	1.371623
H	-0.890245	1.065500	1.468262
C	-4.162825	0.914631	0.613630
H	-4.915769	0.221739	-1.292882
H	-3.123950	1.554705	2.370573
C	1.132624	1.085357	-0.375117
F	2.355638	0.992286	-0.925559
F	0.745575	2.365953	-0.481585
F	1.296836	0.839426	0.938190
O	-5.334916	1.212029	1.214693
O	1.859893	-5.154311	1.215242
C	-6.530568	0.991034	0.481264
H	-6.638711	-0.064369	0.217820
H	-7.343293	1.287400	1.138767
H	-6.551871	1.601412	-0.425331
C	2.829622	-5.953967	0.555306
H	3.760330	-5.399528	0.408931
H	3.010950	-6.803560	1.208099
H	2.455423	-6.306822	-0.409331

P2-TS

Symbol	X	Y	Z
C	1.251844	-1.606829	0.637665
C	0.637609	-1.374659	1.809770
C	0.024364	-0.096988	2.056851
C	0.433102	0.965821	1.180014
C	1.121508	0.731605	0.032025
N	1.408608	-0.597259	-0.305522
H	-0.174820	0.173904	3.086732
H	1.697566	-2.555127	0.376109
H	0.604862	-2.159292	2.552535
H	0.276429	1.994449	1.474962
C	1.666857	1.866349	-0.748901
C	3.041872	1.963873	-0.976958
C	0.820131	2.907717	-1.129379
C	3.557751	3.096999	-1.587487
H	3.691283	1.157594	-0.667881
C	1.341852	4.034588	-1.754077
H	-0.244038	2.831495	-0.939999
C	2.710664	4.130489	-1.981447
H	4.625549	3.172911	-1.752634
H	0.679432	4.835539	-2.058436
H	3.119030	5.011280	-2.462283
S	1.783557	-1.128853	-1.895751
O	4.698526	-1.889263	2.216566
O	2.611765	-0.203230	-2.602568
O	2.042354	-2.537786	-1.823651
C	0.076453	-0.969028	-2.623948
F	0.099438	-1.445027	-3.849650
F	-0.281106	0.305429	-2.641126
F	-0.776081	-1.655146	-1.884424
S	4.442846	-0.832369	1.241174
O	4.174262	-1.300580	-0.125087
O	3.560937	0.244830	1.694371
C	6.077110	0.006929	1.066429
F	6.468184	0.523667	2.229054
F	6.002106	0.998727	0.176293
F	7.007828	-0.849798	0.654637
P	-2.266217	-0.345333	1.642831
C	-2.905464	1.180006	0.943113
C	-2.573665	1.470194	-0.391623
C	-3.599528	2.134226	1.689909
C	-2.964136	2.661124	-0.968464
H	-2.030812	0.748834	-0.992595
C	-3.988391	3.338260	1.117221
H	-3.856758	1.955653	2.726143
C	-3.674883	3.607175	-0.217931
H	-2.729791	2.887135	-2.001481
H	-4.533478	4.051990	1.718576
C	-2.738885	-1.726920	0.572281
C	-2.131431	-2.968388	0.805328
C	-3.649696	-1.566061	-0.466478
C	-2.448912	-4.034591	-0.022138
H	-1.437848	-3.107576	1.623641
C	-3.953049	-2.647470	-1.297348
H	-4.145712	-0.622439	-0.655966
C	-3.344466	-3.884646	-1.077055
H	-1.989102	-5.000070	0.147590
H	-3.562146	-4.732477	-1.711863
C	-3.322807	-0.650773	3.171430
F	-3.018106	0.231978	4.132062
F	-3.076152	-1.871692	3.646634
F	-4.630823	-0.556554	2.933642
O	-4.843657	-2.396264	-2.279492
O	-4.010467	4.736062	-0.863196
C	-5.194947	-3.466883	-3.145404
H	-5.647106	-4.289640	-2.585710
H	-5.920191	-3.058674	-3.843661
H	-4.321232	-3.828002	-3.693922
C	-4.743290	5.724042	-0.147840
H	-4.908720	6.536017	-0.849955
H	-5.703942	5.326713	0.188433
H	-4.170663	6.088782	0.708119

P2-TS-P

Symbol	X	Y	Z
C	0.321808	-0.271575	-1.484292
C	-0.623033	-1.027372	-0.938401
C	-0.790144	-1.122234	0.545582
C	0.420150	-0.597237	1.250954
C	1.365552	0.142183	0.670170
N	1.270828	0.456931	-0.724796
H	-1.014901	-2.142722	0.868761
H	0.449342	-0.216111	-2.554446
H	-1.273743	-1.591336	-1.592731
H	0.549442	-0.837825	2.297270
C	2.508029	0.621532	1.498068
C	3.491484	-0.287884	1.884039
C	2.547546	1.935350	1.958869

C	4.523711	0.127120	2.716175
H	3.446019	-1.299968	1.500064
C	3.585482	2.346438	2.788483
H	1.768451	2.630797	1.671574
C	4.575442	1.444249	3.164703
H	5.291525	-0.577792	3.011397
H	3.615900	3.368508	3.146382
H	5.383494	1.765713	3.810931
S	2.583178	1.027373	-1.617576
O	1.660508	-3.685459	-2.120610
O	3.791976	1.070458	-0.849375
O	2.524148	0.482054	-2.948423
C	2.035457	2.788005	-1.829504
F	2.959621	3.439196	-2.510840
F	1.860741	3.369133	-0.655525
F	0.893921	2.808808	-2.502569
S	2.086001	-3.189321	-0.812881
O	2.789901	-1.902425	-0.835932
O	1.102047	-3.349061	0.261166
C	3.422223	-4.357494	-0.311016
F	2.954143	-5.601124	-0.212641
F	3.924466	-4.009844	0.875304
F	4.414751	-4.355577	-1.197668
P	-2.245794	-0.054125	0.984097
C	-1.887959	1.650285	0.631069
C	-2.153539	2.120519	-0.658745
C	-1.187090	2.462577	1.538993
C	-1.738197	3.384145	-1.047978
H	-2.683640	1.500382	-1.372662
C	-0.783782	3.722943	1.156678
H	-0.952060	2.117031	2.538118
C	-1.036864	4.186857	-0.143042
H	-1.948547	3.721148	-2.052842
H	-0.247269	4.370382	1.838792
C	-3.750065	-0.600984	0.210404
C	-3.872340	-1.921479	-0.228968
C	-4.842547	0.277350	0.099667
C	-5.060125	-2.370418	-0.784869
H	-3.045716	-2.617523	-0.144231
C	-6.024105	-0.166184	-0.448928
H	-4.762641	1.303825	0.438760
C	-6.143725	-1.491978	-0.896635
H	-5.129384	-3.394037	-1.123697
H	-6.878882	0.490216	-0.547120
C	-2.540626	-0.230747	2.839184
F	-2.873867	-1.491200	3.100175
F	-3.533348	0.563430	3.225872
F	-1.457130	0.071331	3.554933
O	-0.561154	5.405251	-0.427609
O	-7.330497	-1.823101	-1.416989
C	-0.755310	5.916672	-1.743144
H	-1.819554	6.024039	-1.964105
H	-0.278594	6.892297	-1.753281
H	-0.282418	5.266818	-2.482721
C	-7.517230	-3.155455	-1.887057
H	-8.537322	-3.196399	-2.256973
H	-6.821226	-3.380017	-2.698109
H	-7.390338	-3.873117	-1.073685

P2-TS-R

Symbol	X	Y	Z
C	0.532095	-0.773382	1.119966
C	0.179233	0.077765	2.131636
C	0.415167	1.441770	1.976294
C	1.052642	1.895399	0.837700
C	1.474797	1.015652	-0.152319
N	1.140457	-0.301268	-0.003066
H	0.135009	2.138614	2.755255
H	0.357827	-1.839259	1.155898
H	-0.272797	-0.325034	3.027097
H	1.310748	2.938889	0.716337
C	2.310077	1.531412	-1.258252
C	3.619012	1.070867	-1.417545
C	1.835812	2.600522	-2.021570
C	4.439272	1.665050	-2.365327
H	3.981436	0.263985	-0.795625
C	2.660066	3.176412	-2.980238
H	0.832693	2.982468	-1.863942
C	3.959570	2.708976	-3.152159
H	5.456360	1.313090	-2.484927
H	2.289184	3.994380	-3.585018
H	4.603611	3.165681	-3.893861
S	1.319395	-1.540766	-1.300532
O	3.688472	-1.796575	3.049018
O	2.442385	-1.274396	-2.134764
O	1.019532	-2.798493	-0.688927
C	-0.221679	-1.045860	-2.235536
F	-0.327404	-1.838253	-3.275997

F	-0.129469	0.210147	-2.619671
F	-1.250564	-1.202243	-1.430001
S	3.869355	-1.075399	1.794134
O	3.430267	-1.798124	0.590684
O	3.473380	0.335173	1.810885
C	5.700032	-1.004357	1.579044
F	6.264762	-0.349505	2.589373
F	6.011856	-0.372395	0.446391
F	6.215153	-2.228549	1.529066
P	-3.772276	0.204498	1.958640
C	-2.886125	1.516555	1.031011
C	-2.523104	1.419354	-0.318820
C	-2.570663	2.685194	1.722461
C	-1.848202	2.450149	-0.943656
H	-2.773667	0.531931	-0.885920
C	-1.906576	3.741957	1.101143
H	-2.840966	2.782214	2.769029
C	-1.531602	3.617946	-0.236015
H	-1.571702	2.386286	-1.989452
H	-1.680403	4.634315	1.668540
C	-3.440072	-1.324902	0.995150
C	-2.517012	-2.204445	1.579519
C	-4.016952	-1.655474	-0.227307
C	-2.161326	-3.373511	0.921269
H	-2.099949	-1.983222	2.555371
C	-3.660747	-2.839791	-0.880673
H	-4.739933	-1.016941	-0.718184
C	-2.724424	-3.701414	-0.308838
H	-1.446881	-4.051803	1.372893
H	-2.432344	-4.618603	-0.801137
C	-5.505495	0.624203	1.339594
F	-5.963872	1.695952	2.007234
F	-6.359060	-0.384392	1.566650
F	-5.600839	0.928571	0.033067
O	-4.266540	-3.051770	-2.071381
O	-0.845233	4.552879	-0.929150
C	-3.868564	-4.189794	-2.821612
H	-4.096994	-5.114558	-2.285656
H	-4.441073	-4.154383	-3.744457
H	-2.799869	-4.149507	-3.050458
C	-0.503028	5.760616	-0.261283
H	0.041969	6.358448	-0.986428
H	-1.401380	6.295546	0.056138
H	0.133687	5.562758	0.604977

P3

Symbol	X	Y	Z
P	-0.089502	-0.164581	-1.478325
C	0.730511	-1.539256	-0.592760
C	0.214530	-2.190143	0.530411
C	1.954293	-1.991843	-1.098809
C	0.894523	-3.232470	1.138493
H	-0.745018	-1.891792	0.936657
C	2.656099	-3.022208	-0.497163
H	2.371832	-1.530321	-1.988469
C	2.147214	-3.668300	0.651610
H	0.446206	-3.710804	1.998118
H	3.597170	-3.332321	-0.929202
C	-1.735696	0.004279	-0.719764
C	-2.833580	-0.220991	-1.558143
C	-2.005562	0.347846	0.613148
C	-4.137692	-0.132649	-1.101628
H	-2.665854	-0.469053	-2.601265
C	-3.300800	0.452408	1.085837
H	-1.192714	0.543353	1.301806
C	-4.409628	0.202583	0.242855
H	-4.945550	-0.315551	-1.795996
H	-3.452624	0.732216	2.118944
C	0.814317	1.242592	-0.600727
F	2.067732	1.345954	-1.083698
F	0.211198	2.422640	-0.819695
F	0.944000	1.119389	0.734010
N	2.837893	-4.680416	1.264515
N	-5.690830	0.285167	0.711543
C	4.038109	-5.208142	0.642011
H	4.438210	-6.004725	1.264597
H	3.838670	-5.614425	-0.356918
H	4.805809	-4.434818	0.550766
C	2.216339	-5.419734	2.347737
H	2.914718	-6.170576	2.709304
H	1.971720	-4.758715	3.183522
H	1.296709	-5.924689	2.027986
C	-5.937162	0.751442	2.064148
H	-7.007984	0.741169	2.251774
H	-5.460312	0.097164	2.799104
H	-5.567302	1.771556	2.219372
C	-6.803667	0.142649	-0.209676
H	-7.734891	0.237292	0.343193

H	-6.785662	0.908473	-0.993747
H	-6.793913	-0.840232	-0.689280

P3-TS

Symbol	X	Y	Z
C	1.400475	-2.003986	0.409166
C	1.223031	-1.422791	1.609976
C	0.961647	-0.021389	1.690644
C	1.178057	0.740103	0.504265
C	1.408492	0.147562	-0.699932
N	1.404209	-1.251437	-0.761174
H	1.104887	0.475512	2.639231
H	1.586252	-3.060811	0.284176
H	1.293085	-2.027144	2.502823
H	1.275432	1.815638	0.562502
C	1.841952	0.953166	-1.860008
C	3.086427	0.697832	-2.442690
C	1.073403	2.034810	-2.284794
C	3.539742	1.513295	-3.467402
H	3.695867	-0.110990	-2.056853
C	1.530434	2.841874	-3.321132
H	0.123049	2.242705	-1.807400
C	2.759943	2.578794	-3.914404
H	4.508011	1.324903	-3.914204
H	0.927685	3.675899	-3.659348
H	3.118555	3.210626	-4.718082
S	1.131311	-2.151479	-2.197747
O	3.803194	0.259257	2.404382
O	1.356569	-1.350143	-3.359801
O	1.716691	-3.445625	-1.996242
C	-0.716950	-2.363101	-2.059650
F	-1.142104	-3.011110	-3.122309
F	-1.279495	-1.167293	-2.009370
F	-0.994786	-3.041686	-0.968396
S	4.405398	0.338030	1.070270
O	4.142939	-0.818950	0.207810
O	4.287860	1.640786	0.416720
C	6.213089	0.192739	1.413473
F	6.624055	1.180971	2.206483
F	6.913042	0.249640	0.281524
F	6.488824	-0.964038	2.014284
P	-1.486928	0.136698	2.014324
C	-2.188109	1.547692	1.149866
C	-2.036114	1.593213	-0.244277
C	-2.793036	2.647307	1.776381
C	-2.493260	2.662653	-0.990470
H	-1.573560	0.762326	-0.765476
C	-3.242444	3.728477	1.045019
H	-2.926746	2.674333	2.849903
C	-3.108649	3.772472	-0.363863
H	-2.370955	2.635491	-2.064489
H	-3.704803	4.548965	1.574717
C	-2.335662	-1.373774	1.464117
C	-1.922893	-2.584052	2.028718
C	-3.316484	-1.336069	0.476360
C	-2.547268	-3.752881	1.606545
H	-1.156979	-2.616382	2.793026
C	-3.924791	-2.523206	0.020887
H	-3.618344	-0.383282	0.067829
C	-3.520848	-3.735538	0.621159
H	-2.257352	-4.701332	2.042857
H	-3.960712	-4.671950	0.308148
C	-2.162143	0.309458	3.763842
F	-1.643829	1.390886	4.363222
F	-1.816701	-0.755896	4.493175
F	-3.492622	0.420058	3.818783
N	-4.876328	-2.492374	-0.970015
N	-3.553613	4.835950	-1.084437
C	-5.288729	-3.742464	-1.585177
H	-5.764197	-4.399630	-0.853701
H	-6.019131	-3.528760	-2.361726
H	-4.443586	-4.278651	-2.035888
C	-5.077912	-1.258122	-1.709651
H	-5.832971	-1.424383	-2.474074
H	-5.439201	-0.462687	-1.053236
H	-4.155843	-0.912941	-2.195610
C	-3.365906	4.866627	-2.524593
H	-3.773173	5.795532	-2.914537
H	-2.304733	4.817875	-2.790532
H	-3.883929	4.034720	-3.011605
C	-4.164273	5.968444	-0.408685
H	-3.468353	6.439012	0.293125
H	-4.453995	6.707611	-1.150612
H	-5.060360	5.666312	0.141542

P3-TS-P

Symbol	X	Y	Z
C	0.414654	-0.369343	-1.432913

C	-0.598992	-1.015442	-0.871318
C	-0.748816	-1.103305	0.615180
C	0.530583	-0.734029	1.296957
C	1.548388	-0.110980	0.702356
N	1.464085	0.230547	-0.688345
H	-1.079091	-2.096278	0.932269
H	0.523750	-0.312736	-2.504777
H	-1.329803	-1.486100	-1.515202
H	0.651016	-0.996956	2.339220
C	2.757821	0.217617	1.509486
C	3.642973	-0.803498	1.852880
C	2.954838	1.507814	1.996036
C	4.734226	-0.524715	2.666300
H	3.477571	-1.795392	1.449565
C	4.051376	1.782572	2.807007
H	2.249783	2.290681	1.743734
C	4.943233	0.768165	3.139507
H	5.425301	-1.317015	2.927647
H	4.203547	2.786429	3.185142
H	5.796884	0.983048	3.771209
S	2.817246	0.640535	-1.603199
O	1.275478	-3.873437	-2.178653
O	4.041888	0.505619	-0.870255
O	2.654254	0.139524	-2.943365
C	2.518436	2.464260	-1.771777
F	3.487201	2.983976	-2.504072
F	2.506692	3.046192	-0.584159
F	1.356313	2.663153	-2.373579
S	1.799700	-3.490369	-0.868561
O	2.671439	-2.310872	-0.868537
O	0.834447	-3.559410	0.231498
C	2.977446	-4.848860	-0.456442
F	2.349142	-6.023100	-0.406117
F	3.544745	-4.632565	0.731638
F	3.944548	-4.934248	-1.367367
P	-2.071606	0.118789	1.075515
C	-1.565141	1.760719	0.663378
C	-1.863363	2.245050	-0.622194
C	-0.676364	2.490816	1.469557
C	-1.296241	3.407892	-1.090002
H	-2.541507	1.697945	-1.267572
C	-0.111332	3.660631	1.012126
H	-0.416100	2.147421	2.463572
C	-0.370138	4.135187	-0.299420
H	-1.546739	3.741641	-2.086414
H	0.557508	4.201860	1.666104
C	-3.636614	-0.314483	0.378131
C	-3.902063	-1.629550	-0.036842
C	-4.662631	0.642183	0.293909
C	-5.138598	-1.977442	-0.530988
H	-3.138231	-2.396818	0.021745
C	-5.903314	0.304395	-0.195301
H	-4.483610	1.663518	0.612055
C	-6.182597	-1.020070	-0.626474
H	-5.300533	-2.997519	-0.846570
H	-6.665129	1.068444	-0.246613
C	-2.284932	0.003250	2.946645
F	-2.662478	-1.235400	3.258945
F	-3.225297	0.849321	3.358350
F	-1.161481	0.272144	3.616105
N	-7.398932	-1.356834	-1.111926
N	0.256522	5.232702	-0.784053
C	-8.455813	-0.359482	-1.194415
H	-8.166352	0.471078	-1.844449
H	-9.346221	-0.823691	-1.608760
H	-8.702760	0.038626	-0.206043
C	-7.660369	-2.720898	-1.548390
H	-6.993564	-3.009215	-2.365861
H	-7.533906	-3.431070	-0.726280
H	-8.685028	-2.786471	-1.902979
C	-0.033760	5.704872	-2.130022
H	0.584207	6.573609	-2.339133
H	0.194612	4.934460	-2.871089
H	-1.083868	5.993878	-2.234892
C	1.177284	5.980838	0.059002
H	2.012717	5.352830	0.379966
H	1.577186	6.815036	-0.510558
H	0.674017	6.377733	0.945717

P3-TS-R

Symbol	X	Y	Z
C	-0.725367	-1.270722	0.571218
C	-0.003278	-2.104216	-0.245073
C	0.029296	-1.835490	-1.609327
C	-0.653459	-0.735562	-2.109227
C	-1.385230	0.093781	-1.273206
N	-1.378596	-0.197110	0.060304
H	0.576491	-2.486340	-2.279793

H	-0.823387	-1.436915	1.634383
H	0.513216	-2.952239	0.185442
H	-0.655871	-0.508368	-3.166541
C	-2.139529	1.234773	-1.859084
C	-3.482808	1.066222	-2.196215
C	-1.464460	2.409672	-2.183244
C	-4.157327	2.100366	-2.829066
H	-3.989446	0.142507	-1.946614
C	-2.151901	3.441756	-2.814017
H	-0.410456	2.519985	-1.952845
C	-3.496440	3.288461	-3.132532
H	-5.202555	1.975680	-3.083536
H	-1.631936	4.358875	-3.060750
H	-4.028997	4.092551	-3.625635
S	-2.367066	0.709103	1.267749
O	-5.322181	-3.151867	0.512933
O	-3.442092	1.388089	0.628715
O	-2.481381	-0.158761	2.397966
C	-1.066555	1.974794	1.717869
F	-1.491304	2.612007	2.782843
F	-0.901214	2.812966	0.716267
F	0.062488	1.346515	1.977844
S	-4.989259	-1.977494	-0.283575
O	-3.629441	-1.453742	-0.067362
O	-5.393359	-2.016723	-1.687516
C	-6.055925	-0.647457	0.423231
F	-7.327183	-1.040842	0.461781
F	-5.986734	0.456877	-0.316653
F	-5.676168	-0.347280	1.664181
P	3.342499	-0.857591	-1.895178
C	3.549582	0.692322	-0.965182
C	2.373166	1.390229	-0.666239
C	4.751254	1.218613	-0.474427
C	2.381046	2.547787	0.091312
H	1.418724	1.013995	-1.027976
C	4.781756	2.388477	0.264247
H	5.691825	0.719058	-0.667507
C	3.593003	3.084615	0.581194
H	1.442962	3.024401	0.329488
H	5.738524	2.757808	0.605948
C	3.206238	-2.140575	-0.579563
C	3.024263	-3.465382	-0.986438
C	3.169398	-1.798705	0.767199
C	2.827728	-4.439507	-0.011979
H	3.039568	-3.733090	-2.037086
C	2.933412	-2.775366	1.760260
H	3.314451	-0.765622	1.049848
C	2.774916	-4.113603	1.336050
H	2.700333	-5.475039	-0.305786
H	2.607040	-4.900271	2.058364
C	5.124397	-1.229208	-2.384577
F	5.639642	-0.235642	-3.126335
F	5.163483	-2.343716	-3.134497
F	5.973988	-1.433057	-1.364072
N	2.851936	-2.429285	3.083785
N	3.611477	4.224944	1.330398
C	2.764271	-3.468898	4.093679
H	3.618301	-4.154299	4.047998
H	2.747248	-3.005881	5.077164
H	1.847211	-4.054513	3.980585
C	3.111407	-1.058758	3.484753
H	2.981303	-0.974677	4.560849
H	4.129554	-0.739981	3.231112
H	2.409420	-0.369296	3.005179
C	2.374697	4.936371	1.595963
H	2.594599	5.821346	2.187240
H	1.883580	5.253993	0.669618
H	1.672476	4.317153	2.164016
C	4.877976	4.783453	1.769364
H	5.519828	5.043645	0.920926
H	4.686403	5.686117	2.343824
H	5.421077	4.081813	2.409784

P4

Symbol	X	Y	Z
P	-0.012958	2.019570	0.940675
C	-1.440646	0.923864	0.620657
C	-1.370952	-0.312433	-0.028915
C	-2.694511	1.351125	1.074665
C	-2.500605	-1.085694	-0.232878
H	-0.415568	-0.686672	-0.378670
C	-3.838220	0.599130	0.870389
H	-2.779680	2.294585	1.605323
C	-3.769662	-0.645441	0.206014
H	-2.406104	-2.033783	-0.746124
H	-4.786118	0.962234	1.245329
C	1.469205	1.038950	0.545723
C	2.321288	0.745150	1.617964

C	1.838145	0.559433	-0.720721
C	3.474332	-0.003007	1.456144
H	2.078844	1.117390	2.608253
C	2.991514	-0.179932	-0.905984
H	1.216193	0.761892	-1.584099
C	3.841567	-0.487469	0.182057
H	4.106502	-0.195292	2.312896
H	3.235006	-0.535987	-1.898495
C	-0.150152	3.048827	-0.636848
F	-1.162740	3.929127	-0.515256
F	0.966006	3.763637	-0.855648
F	-0.394518	2.362897	-1.770078
C	5.830228	-1.658430	1.102338
C	5.470561	-1.639968	-1.302312
C	6.885127	-2.133845	-0.994422
C	6.759927	-2.671917	0.433570
H	6.391299	-0.814204	1.523475
H	5.236757	-2.105197	1.904309
H	4.846712	-2.440574	-1.719987
H	5.467962	-0.804591	-2.007277
H	7.228333	-2.880844	-1.709189
H	7.579991	-1.290738	-1.014522
H	6.282072	-3.654588	0.420511
H	7.716373	-2.760088	0.947488
C	-4.860994	-2.744556	-0.561186
C	-6.234144	-0.949525	0.334315
C	-7.123995	-1.986493	-0.352686
C	-6.276132	-3.260045	-0.294210
H	-4.639038	-2.715267	-1.635832
H	-4.098290	-3.358250	-0.074416
H	-6.391185	-0.941670	1.420830
H	-6.410432	0.062014	-0.040963
H	-8.093191	-2.089804	0.133908
H	-7.288220	-1.696274	-1.393342
H	-6.324984	-3.693259	0.707864
H	-6.584516	-4.018201	-1.013146
N	4.972756	-1.219176	0.004886
N	-4.884807	-1.398493	0.004051

P4-TS

Symbol	X	Y	Z
C	1.781239	-2.208878	0.028392
C	1.227120	-2.408767	1.235534
C	0.732994	-1.284941	1.989469
C	1.227976	-0.004335	1.577228
C	1.793665	0.186904	0.357377
N	1.951530	-0.923103	-0.478460
H	0.559767	-1.431905	3.048897
H	2.150671	-3.004062	-0.601975
H	1.152449	-3.414512	1.624893
H	1.174934	0.842108	2.247853
C	2.281727	1.532461	-0.020646
C	3.623269	1.760593	-0.330832
C	1.386113	2.598619	0.056898
C	4.056621	3.058070	-0.564137
H	4.308228	0.925254	-0.377438
C	1.822399	3.894033	-0.193164
H	0.348961	2.400514	0.298086
C	3.159908	4.123625	-0.501038
H	5.099467	3.238068	-0.795068
H	1.116554	4.715949	-0.147209
H	3.506925	5.132350	-0.690662
S	2.139793	-0.823176	-2.180385
O	5.404976	-3.126028	0.952514
O	2.960896	0.279245	-2.572533
O	2.304434	-2.160107	-2.673671
C	0.386304	-0.356427	-2.609651
F	0.251483	-0.421127	-3.917878
F	0.130548	0.867748	-2.188727
F	-0.444455	-1.207549	-2.029342
S	5.110687	-1.784950	0.453627
O	4.661737	-1.725818	-0.942685
O	4.358281	-0.924173	1.368312
C	6.766505	-0.973475	0.381265
F	7.318366	-0.919307	1.591483
F	6.657872	0.274565	-0.080023
F	7.588329	-1.641783	-0.423805
P	-1.586299	-1.039954	1.619953
C	-1.891301	0.682582	1.232111
C	-2.141945	1.077528	-0.090366
C	-1.720072	1.683781	2.203249
C	-2.228970	2.411902	-0.435408
H	-2.273346	0.329439	-0.863681
C	-1.813123	3.019958	1.874618
H	-1.521490	1.422036	3.236812
C	-2.047398	3.425368	0.536386
H	-2.404457	2.676254	-1.469464
H	-1.697509	3.761974	2.652840

C	-2.583061	-2.065020	0.509292
C	-2.022240	-3.240043	0.010590
C	-3.889035	-1.683245	0.200927
C	-2.806704	-4.036956	-0.822159
H	-1.008862	-3.527516	0.254983
C	-4.680030	-2.488720	-0.642199
H	-4.296405	-0.771165	0.617963
C	-4.101345	-3.676973	-1.148562
H	-2.394759	-4.951994	-1.230327
H	-4.676224	-4.310773	-1.811135
C	-2.467202	-1.276971	3.269726
F	-1.739920	-0.735195	4.262451
F	-2.601045	-2.576911	3.536496
F	-3.679047	-0.720860	3.314094
C	-6.867913	-2.981740	-1.718546
C	-6.553424	-0.860472	-0.568667
C	-7.837018	-0.813544	-1.398798
C	-8.218983	-2.290711	-1.527287
H	-6.580956	-3.019089	-2.777174
H	-6.866069	-4.002976	-1.328742
H	-6.768224	-0.842155	0.507602
H	-5.881161	-0.027684	-0.794904
H	-8.611241	-0.206684	-0.931100
H	-7.620214	-0.397947	-2.385842
H	-8.679366	-2.637905	-0.599188
H	-8.906010	-2.489127	-2.348914
N	-5.952716	-2.133082	-0.958939
C	-1.756429	5.819606	1.129083
C	-2.369458	5.218954	-1.149551
C	-2.542988	6.724238	-0.947620
C	-1.588319	7.030135	0.209493
H	-2.572599	5.966621	1.846252
H	-0.845129	5.593351	1.688837
H	-1.537464	4.994906	-1.829035
H	-3.271564	4.744788	-1.542849
H	-2.320757	7.290529	-1.850958
H	-3.572044	6.937915	-0.649054
H	-0.560058	7.073949	-0.158057
H	-1.811570	7.967536	0.716915
N	-2.074348	4.734366	0.199315

P4-TS-P

Symbol	X	Y	Z
C	0.509217	-0.570375	-1.369859
C	-0.585595	-1.048365	-0.792423
C	-0.692583	-1.193752	0.693180
C	0.658707	-1.108877	1.329140
C	1.752476	-0.653393	0.718000
N	1.680929	-0.220741	-0.648110
H	-1.189876	-2.125710	0.975664
H	0.585543	-0.470877	-2.441473
H	-1.416814	-1.335104	-1.422727
H	0.767709	-1.451609	2.349254
C	3.032639	-0.609069	1.480567
C	3.721776	-1.797885	1.716530
C	3.486477	0.585002	2.035586
C	4.874187	-1.783993	2.492062
H	3.357302	-2.711053	1.261457
C	4.643519	0.594389	2.808156
H	2.932907	1.500751	1.867010
C	5.339545	-0.588511	3.034062
H	5.412284	-2.707145	2.670616
H	4.995064	1.523792	3.239990
H	6.239989	-0.580470	3.636664
S	3.051616	-0.017110	-1.604483
O	0.648392	-4.128665	-2.295420
O	4.253752	-0.435069	-0.945001
O	2.741512	-0.382051	-2.962640
C	3.109139	1.837468	-1.649491
F	4.134664	2.211517	-2.394405
F	3.251602	2.330372	-0.431008
F	1.988139	2.295617	-2.184475
S	1.320236	-3.925076	-1.012742
O	2.397751	-2.929694	-1.025777
O	0.433297	-3.877553	0.152282
C	2.244903	-5.499779	-0.751016
F	1.409635	-6.536383	-0.686971
F	2.933826	-5.453877	0.390638
F	3.103680	-5.719814	-1.744322
P	-1.744070	0.221455	1.282888
C	-0.960692	1.763899	0.929428
C	-1.226348	2.375590	-0.309646
C	0.093446	2.260119	1.716130
C	-0.470033	3.435601	-0.751250
H	-2.032973	2.010673	-0.935711
C	0.850833	3.325688	1.285048
H	0.333955	1.809022	2.671156
C	0.621321	3.916403	0.016085

H	-0.703373	3.888510	-1.704784
H	1.655415	3.686080	1.911705
C	-3.390545	0.125546	0.650684
C	-3.912977	-1.090074	0.176988
C	-4.222786	1.259336	0.674260
C	-5.210642	-1.172080	-0.272498
H	-3.304121	-1.986982	0.159553
C	-5.522375	1.188184	0.230822
H	-3.840609	2.207562	1.036666
C	-6.057246	-0.032737	-0.260105
H	-5.585714	-2.122625	-0.625246
H	-6.133677	2.079766	0.245227
C	-1.891539	0.029493	3.154245
F	-2.460828	-1.145331	3.421117
F	-2.656154	0.994659	3.658215
F	-0.712135	0.065345	3.779257
N	-7.326249	-0.105979	-0.701299
N	1.427205	4.888962	-0.450367
C	1.342941	5.445813	-1.803386
C	2.534313	5.462533	0.320264
C	3.011674	6.621023	-0.556385
C	2.695625	6.135720	-1.973097
H	1.185423	4.652524	-2.535623
H	0.513403	6.158769	-1.876776
H	2.190834	5.796060	1.302179
H	3.322021	4.714240	0.464261
H	2.430083	7.518216	-0.331820
H	4.065926	6.845442	-0.401467
H	2.662555	6.938066	-2.708676
H	3.440790	5.402093	-2.291919
C	-8.283514	1.000778	-0.618632
C	-7.907013	-1.300851	-1.320501
C	-9.216005	-0.783195	-1.916549
C	-9.618185	0.333213	-0.949905
H	-8.275261	1.443150	0.379776
H	-8.030131	1.781064	-1.345834
H	-7.234463	-1.706271	-2.079198
H	-8.084959	-2.075242	-0.565369
H	-9.028274	-0.368068	-2.909532
H	-9.966029	-1.567602	-2.006311
H	-10.335023	1.034455	-1.374597
H	-10.049149	-0.095901	-0.042325

P4-TS-R

Symbol	X	Y	Z
C	1.882072	-2.161571	0.761972
C	1.700918	-2.117228	2.118446
C	1.498059	-0.882198	2.730069
C	1.582592	0.272725	1.974069
C	1.858452	0.220069	0.613195
N	1.925087	-1.014854	0.033755
H	1.307644	-0.822251	3.793818
H	1.998396	-3.082779	0.209415
H	1.693059	-3.041881	2.677785
H	1.495591	1.250527	2.428379
C	2.100435	1.479698	-0.123033
C	3.346348	1.748320	-0.693866
C	1.103895	2.454097	-0.100639
C	3.572824	2.991275	-1.268564
H	4.117066	0.990695	-0.671749
C	1.331916	3.687736	-0.696589
H	0.150023	2.232242	0.359936
C	2.567516	3.955710	-1.278965
H	4.539016	3.206521	-1.707580
H	0.542809	4.431758	-0.700997
H	2.751780	4.920462	-1.736275
S	1.944336	-1.271472	-1.748233
O	5.656745	-2.662215	1.413101
O	2.764832	-0.308432	-2.403530
O	2.033473	-2.687705	-1.930317
C	0.144761	-0.826970	-2.058404
F	-0.169639	-1.323970	-3.232830
F	0.001601	0.477778	-2.056872
F	-0.593747	-1.372995	-1.116485
S	5.192240	-1.517997	0.636534
O	4.515391	-1.853545	-0.623675
O	4.522402	-0.462104	1.402282
C	6.751619	-0.712374	0.065956
F	7.493558	-0.341749	1.105739
F	6.480515	0.373432	-0.660244
F	7.463095	-1.547986	-0.684275
P	-2.095388	-0.582181	2.433496
C	-2.093093	1.000674	1.510829
C	-2.338061	1.157785	0.142221
C	-1.823549	2.157302	2.258164
C	-2.328202	2.407406	-0.456195
H	-2.526713	0.291113	-0.478562
C	-1.825229	3.415331	1.681883

H	-1.615206	2.075500	3.320699
C	-2.070493	3.572575	0.298915
H	-2.492538	2.479994	-1.523481
H	-1.628346	4.281035	2.300308
C	-2.516342	-1.887218	1.215965
C	-1.676014	-3.002692	1.215784
C	-3.593267	-1.816742	0.332202
C	-1.909706	-4.028667	0.302270
H	-0.860152	-3.074831	1.922879
C	-3.835642	-2.850192	-0.595412
H	-4.260324	-0.966605	0.363223
C	-2.959297	-3.959192	-0.596637
H	-1.259429	-4.895931	0.288811
H	-3.108343	-4.762537	-1.306482
C	-3.793789	-0.373542	3.237096
F	-3.716896	0.525886	4.236924
F	-4.209853	-1.528536	3.778964
F	-4.779089	0.053156	2.428751
C	-5.277115	-3.867844	-2.351712
C	-5.746829	-1.608152	-1.586355
C	-6.554973	-1.902909	-2.850912
C	-6.657463	-3.430714	-2.845541
H	-4.568955	-3.978257	-3.183277
H	-5.313256	-4.816472	-1.809277
H	-6.396647	-1.507066	-0.707109
H	-5.155497	-0.691923	-1.675813
H	-7.523915	-1.405120	-2.849978
H	-5.996020	-1.570697	-3.729228
H	-7.419628	-3.749237	-2.130219
H	-6.902150	-3.850687	-3.820458
N	-4.880706	-2.776487	-1.466731
C	-1.613587	6.016955	0.404489
C	-2.336371	5.028817	-1.698572
C	-2.396931	6.553782	-1.796240
C	-1.387301	7.008021	-0.738573
H	-2.398412	6.361366	1.089151
H	-0.704864	5.844657	0.989132
H	-1.552074	4.613552	-2.346125
H	-3.284319	4.556408	-1.968300
H	-2.164880	6.914653	-2.797404
H	-3.398099	6.898925	-1.527302
H	-0.369801	6.898979	-1.124109
H	-1.525246	8.041747	-0.424463
N	-2.021883	4.798024	-0.290516

P5

Symbol	X	Y	Z
P	-0.362321	-0.455670	-1.716672
C	0.333163	-1.868167	-0.768173
C	-0.522956	-2.827421	-0.211108
C	1.707721	-2.082267	-0.684880
C	-0.018279	-3.950610	0.419002
H	-1.597856	-2.690467	-0.261389
C	2.228826	-3.206850	-0.048601
H	2.399596	-1.364925	-1.110017
C	1.363285	-4.148576	0.506695
H	-0.675500	-4.692660	0.855625
H	3.301701	-3.331014	0.004469
C	-1.783683	0.042718	-0.672735
C	-2.974122	0.392388	-1.304383
C	-1.729532	0.097860	0.730104
C	-4.093244	0.798858	-0.578500
H	-3.040585	0.348109	-2.386078
C	-2.828893	0.497368	1.462197
H	-0.817421	-0.181326	1.244782
C	-4.019994	0.852388	0.812121
H	-5.000901	1.061121	-1.104072
H	-2.800022	0.544763	2.543915
C	0.825744	0.894107	-1.167703
F	1.978820	0.816936	-1.902946
F	1.204363	0.797567	0.138070
O	-5.038319	1.226922	1.617236
O	1.762357	-5.275295	1.144519
C	-6.266294	1.592065	1.005652
H	-6.686421	0.755434	0.441165
H	-6.937833	1.856928	1.817868
H	-6.133687	2.452665	0.344672
C	3.156591	-5.512763	1.251990
H	3.648658	-4.715840	1.816324
H	3.260246	-6.453481	1.786361
H	3.617512	-5.601039	0.264345
H	0.388798	1.882104	-1.322177

P5-TS

Symbol	X	Y	Z
C	1.048924	-1.934959	0.167061
C	0.442271	-2.021227	1.362721
C	-0.026610	-0.825056	2.011496

C	0.535200	0.400003	1.527269
C	1.155962	0.475942	0.321012
N	1.296533	-0.699161	-0.423129
H	-0.253528	-0.884439	3.069422
H	1.402175	-2.790435	-0.389662
H	0.308291	-2.990541	1.821787
H	0.493671	1.295077	2.132508
C	1.723261	1.765646	-0.132357
C	3.087747	1.902020	-0.393270
C	0.882834	2.877888	-0.179455
C	3.601080	3.153511	-0.701068
H	3.727022	1.031535	-0.346607
C	1.400035	4.125818	-0.505816
H	-0.174680	2.752990	0.024170
C	2.760397	4.263554	-0.763058
H	4.661410	3.261931	-0.893858
H	0.740238	4.983585	-0.560982
H	3.168017	5.236064	-1.011857
S	1.556916	-0.733867	-2.119974
O	4.577550	-2.938041	1.280126
O	2.437075	0.303567	-2.557056
O	1.684538	-2.109770	-2.505133
C	-0.160053	-0.233497	-2.649988
F	-0.259240	-0.411717	-3.950076
F	-0.372097	1.033036	-2.348053
F	-1.046553	-0.992670	-2.023716
S	4.370502	-1.610562	0.706849
O	3.998559	-1.597994	-0.713346
O	3.609193	-0.676437	1.538370
C	6.062636	-0.874555	0.689413
F	6.553397	-0.792144	1.924061
F	6.034100	0.355739	0.172405
F	6.893401	-1.612011	-0.042731
P	-2.330730	-0.536625	1.552380
C	-2.561175	1.158560	0.981145
C	-2.738890	1.426489	-0.385776
C	-2.421049	2.232213	1.865550
C	-2.808725	2.727738	-0.841746
H	-2.836199	0.613025	-1.094778
C	-2.496232	3.544997	1.414755
H	-2.255759	2.060018	2.922672
C	-2.691358	3.798946	0.053943
H	-2.947415	2.945576	-1.893198
H	-2.394318	4.351377	2.127344
C	-3.328734	-1.620634	0.494959
C	-2.822921	-2.883096	0.167563
C	-4.587865	-1.220824	0.055150
C	-3.598818	-3.733576	-0.608223
H	-1.844216	-3.196858	0.507288
C	-5.358436	-2.087065	-0.723736
H	-4.995578	-0.244706	0.292132
C	-4.857863	-3.348357	-1.057166
H	-3.220854	-4.713085	-0.873275
H	-5.437123	-4.032383	-1.661973
C	-3.238441	-0.609171	3.190963
F	-2.490870	0.058323	4.114180
F	-3.289038	-1.905273	3.574333
O	-6.564950	-1.617653	-1.103603
O	-2.763970	5.028139	-0.486486
C	-7.386829	-2.462589	-1.897682
H	-7.629485	-3.385131	-1.364218
H	-8.297046	-1.900090	-2.085669
H	-6.900342	-2.700635	-2.847039
C	-2.648164	6.149148	0.381681
H	-2.740224	7.025979	-0.252747
H	-3.447519	6.148348	1.126536
H	-1.675306	6.156562	0.879521
H	-4.245681	-0.190596	3.180064

P5-TS-p

Symbol	X	Y	Z
C	0.237140	-0.355185	-1.403608
C	-0.676920	-1.104236	-0.799560
C	-0.803384	-1.141617	0.691118
C	0.413001	-0.559692	1.340073
C	1.326252	0.173471	0.703759
N	1.190407	0.424269	-0.701146
H	-0.982585	-2.157132	1.055421
H	0.336293	-0.342623	-2.478167
H	-1.333368	-1.707449	-1.412066
H	0.571832	-0.746756	2.392925
C	2.476119	0.715678	1.481185
C	3.506707	-0.145079	1.855735
C	2.476736	2.040403	1.911244
C	4.546508	0.329448	2.645561
H	3.489834	-1.167213	1.496512
C	3.522987	2.511587	2.697788
H	1.659660	2.696442	1.635654

C	4.559263	1.658115	3.062307
H	5.350616	-0.337391	2.932540
H	3.523424	3.542150	3.031905
H	5.373302	2.026186	3.675331
S	2.455997	1.002898	-1.647593
O	1.576596	-3.775447	-1.955307
O	3.686638	1.102547	-0.919348
O	2.373299	0.416979	-2.959665
C	1.859270	2.742007	-1.897606
F	2.762995	3.403353	-2.596923
F	1.670570	3.346090	-0.737338
F	0.716262	2.715263	-2.568141
S	2.054561	-3.221029	-0.689562
O	2.738279	-1.928106	-0.797002
O	1.123242	-3.350729	0.434448
C	3.428347	-4.352474	-0.204994
F	2.984466	-5.599185	-0.047574
F	3.972827	-3.959496	0.947995
F	4.384008	-4.365525	-1.131232
P	-2.277516	-0.103475	1.144075
C	-1.972702	1.591566	0.693289
C	-2.233997	1.993672	-0.618923
C	-1.325931	2.471466	1.576876
C	-1.860888	3.255444	-1.056913
H	-2.727380	1.321859	-1.312379
C	-0.960390	3.728116	1.145342
H	-1.105023	2.177331	2.595395
C	-1.206116	4.123078	-0.177829
H	-2.067142	3.540524	-2.078542
H	-0.461502	4.424945	1.807049
C	-3.778362	-0.722502	0.406497
C	-3.877281	-2.063060	0.027647
C	-4.891498	0.124426	0.269278
C	-5.061563	-2.563057	-0.493573
H	-3.034163	-2.736505	0.131829
C	-6.069497	-0.368393	-0.246075
H	-4.829690	1.166436	0.561861
C	-6.164864	-1.714468	-0.633107
H	-5.111360	-3.601972	-0.786273
H	-6.939178	0.264833	-0.364278
C	-2.544390	-0.181421	2.998937
F	-3.465108	0.752035	3.320730
F	-1.399391	0.113683	3.656544
O	-0.769562	5.345972	-0.510389
O	-7.350568	-2.094542	-1.126014
C	-0.943746	5.781846	-1.854833
H	-2.004197	5.842001	-2.109839
H	-0.498780	6.771265	-1.904471
H	-0.430380	5.110993	-2.547450
C	-7.510431	-3.449726	-1.534855
H	-8.531877	-3.530236	-1.894608
H	-6.815314	-3.695711	-2.340650
H	-7.361849	-4.127975	-0.691702
H	-2.895465	-1.167493	3.306495

P5-TS-R

Symbol	X	Y	Z
C	1.281415	-1.934326	0.739798
C	0.945431	-1.843419	2.063271
C	0.699591	-0.584960	2.609675
C	0.886037	0.542547	1.831825
C	1.299438	0.441692	0.509650
N	1.418377	-0.812822	-0.017637
H	0.392116	-0.487459	3.642655
H	1.450504	-2.874488	0.234852
H	0.855656	-2.749210	2.645672
H	0.764650	1.535912	2.242120
C	1.605886	1.675133	-0.247111
C	2.902743	1.946896	-0.684967
C	0.592337	2.621080	-0.392404
C	3.166964	3.164644	-1.296170
H	3.682496	1.212106	-0.538227
C	0.858159	3.825541	-1.030131
H	-0.400616	2.394339	-0.025788
C	2.147516	4.096635	-1.479341
H	4.172430	3.384326	-1.632766
H	0.057566	4.540937	-1.180197
H	2.361417	5.038582	-1.969763
S	1.627691	-1.134445	-1.776334
O	4.977176	-2.411847	1.796897
O	2.481431	-0.170392	-2.385030
O	1.764857	-2.553132	-1.897374
C	-0.141504	-0.754620	-2.280424
F	-0.318309	-1.284099	-3.468384
F	-0.327116	0.544039	-2.320961
F	-0.960142	-1.306456	-1.406608
S	4.599240	-1.281396	0.955968
O	4.071074	-1.638323	-0.368468

O	3.842858	-0.219085	1.625678
C	6.206067	-0.469730	0.554590
F	6.816775	-0.066786	1.664995
F	6.008763	0.596740	-0.222326
F	7.010512	-1.310015	-0.088322
P	-2.741656	-0.297484	1.890641
C	-2.747313	1.270192	0.924144
C	-2.846419	1.346855	-0.472055
C	-2.577415	2.460710	1.637240
C	-2.799211	2.568836	-1.122417
H	-2.957436	0.445386	-1.061490
C	-2.542598	3.696574	0.997249
H	-2.473572	2.436088	2.716604
C	-2.654027	3.753291	-0.393182
H	-2.867773	2.631001	-2.201659
H	-2.416339	4.593117	1.588319
C	-3.336274	-1.565756	0.703410
C	-2.621458	-2.766730	0.641825
C	-4.469667	-1.398194	-0.087347
C	-3.038980	-3.772201	-0.219725
H	-1.746509	-2.909716	1.265130
C	-4.887764	-2.413328	-0.951197
H	-5.048883	-0.481221	-0.074051
C	-4.165001	-3.606651	-1.020634
H	-2.485547	-4.701887	-0.273855
H	-4.468734	-4.404688	-1.684138
C	-4.370786	-0.040276	2.794354
F	-4.208981	0.944132	3.725823
F	-4.666472	-1.182869	3.472189
O	-5.996902	-2.146839	-1.679373
O	-2.599541	4.895562	-1.117355
C	-6.466128	-3.153445	-2.563660
H	-6.729931	-4.062973	-2.017525
H	-7.353803	-2.743883	-3.038099
H	-5.718244	-3.385674	-3.326507
C	-2.493943	6.122830	-0.409152
H	-2.495919	6.904327	-1.164067
H	-3.344258	6.259741	0.263614
H	-1.561820	6.167481	0.161257
H	-5.223633	0.224678	2.167448
